# Signaling pathways involved in colorectal cancer: pathogenesis and targeted therapy

**DOI:** 10.1038/s41392-024-01953-7

**Published:** 2024-10-07

**Authors:** Qing Li, Shan Geng, Hao Luo, Wei Wang, Ya-Qi Mo, Qing Luo, Lu Wang, Guan-Bin Song, Jian-Peng Sheng, Bo Xu

**Affiliations:** 1 The Shapingba Hospital, Chongqing University, Chongqing, China; 2 Chongqing Key Laboratory of Intelligent Oncology for Breast Cancer, Chongqing University Cancer Hospital and School of Medicine, Chongqing University, Chongqing, China; 3 Key Laboratory of Biorheological Science and Technology, Ministry of Education, College of Bioengineering, Chongqing University, Chongqing, China; 4 Central Laboratory, The Affiliated Dazu Hospital of Chongqing Medical University, Chongqing, China; 5 Cancer Center, Daping Hospital, Army Medical University, Chongqing, China; 6 Chongqing Municipal Health and Health Committee, Chongqing, China; 7 College of Artificial Intelligence, Nanjing University of Aeronautics and Astronautics, Nanjing, China

**Keywords:** Gastrointestinal cancer, Gastrointestinal cancer

## Abstract

Colorectal cancer (CRC) remains one of the leading causes of cancer-related mortality worldwide. Its complexity is influenced by various signal transduction networks that govern cellular proliferation, survival, differentiation, and apoptosis. The pathogenesis of CRC is a testament to the dysregulation of these signaling cascades, which culminates in the malignant transformation of colonic epithelium. This review aims to dissect the foundational signaling mechanisms implicated in CRC, to elucidate the generalized principles underpinning neoplastic evolution and progression. We discuss the molecular hallmarks of CRC, including the genomic, epigenomic and microbial features of CRC to highlight the role of signal transduction in the orchestration of the tumorigenic process. Concurrently, we review the advent of targeted and immune therapies in CRC, assessing their impact on the current clinical landscape. The development of these therapies has been informed by a deepening understanding of oncogenic signaling, leading to the identification of key nodes within these networks that can be exploited pharmacologically. Furthermore, we explore the potential of integrating AI to enhance the precision of therapeutic targeting and patient stratification, emphasizing their role in personalized medicine. In summary, our review captures the dynamic interplay between aberrant signaling in CRC pathogenesis and the concerted efforts to counteract these changes through targeted therapeutic strategies, ultimately aiming to pave the way for improved prognosis and personalized treatment modalities in colorectal cancer.

## Introduction

Colorectal cancer (CRC) is a formidable global health adversary, consistently ranking as the third most prevalent and second most lethal malignancy worldwide.^1^ The disease claims more than 900,000 lives annually, with its incidence demonstrating a worrying ascendancy in populations traditionally considered at lower risk.^2^ This epidemiological burden underscores an urgent need for a nuanced understanding of CRC’s pathophysiology and the development of innovative therapeutic strategies.

At the core of CRC’s pathogenesis lie aberrant signaling pathways that drive tumorigenesis, sustain cancer cell proliferation, and enable metastatic dissemination.^3^ These pathways, which include the Wnt/β-catenin, RAS/RAF/MEK/ERK, phosphoinositide 3-kinase (PI3K)/AKT, and transforming growth factor-beta (TGF-β) circuits, among others, are often dysregulated by a confluence of genetic mutations (means somatic variants or germline variants), such as adenomatous polyposis coli (APC), kirsten rat sarcoma viral oncogene homolog (KRAS), and PIK3CA.^4^ The intricate network of signaling cascades they form dictates not only the malignant phenotype but also the immune response and the tumor microenvironment (TME), influencing the efficacy of therapeutic interventions.

The landscape of targeted therapy has evolved apace, as our molecular insight into CRC has deepened. Inhibitors of epidermal growth factor receptor (EGFR), such as cetuximab and panitumumab, have become stalwarts in the management of metastatic CRC, albeit their efficacy is often thwarted by intrinsic or acquired resistance mechanisms.^5^ Concurrently, the dawn of precision medicine has signaled a shift from the one-size-fits-all approach toward a more bespoke treatment paradigm. This paradigm leverages the burgeoning field of multi-omics, which integrates genomic, transcriptomic, proteomic, and metabolomic data to tailor therapies to the individual molecular profile of a patient’s tumor.^6^

In summary, as we navigate the complex oncogenic signaling networks and the shifting sands of therapeutic landscapes, our collective endeavor is to transition from the blunt tools of traditional chemotherapy to the scalpel of precision oncology. This transition promises not only to enhance the precision of CRC management but also to improve the prognosis for patients worldwide, heralding a new epoch in cancer care.

## Global epidemiology of CRC

The global landscape of CRC presents a complex mosaic of incidence and mortality, shaped by a web of influences that span from socioeconomic progress to lifestyle changes and beyond. The International Agency for Research on Cancer (IARC) reported in 2018 that CRC stands as the third most frequently diagnosed cancer and the second leading cause of cancer-related death worldwide, with about 1.8 million new cases and 900,000 deaths annually.^7^ The disparity in CRC incidences is stark, with a more than 45-fold difference observed between countries with the highest and lowest rates, exemplified by Hungary and the Gambia, respectively.^8^ This discrepancy underscores the multifaceted nature of CRC etiology, which includes lifestyle choices, genetic predispositions, and the varying effectiveness of healthcare systems, particularly cancer registry databases.

Temporal shifts in the incidence of CRC have been noted globally. Regions such as South America, Eastern Europe, and Asia, which are in the midst of economic transition, have reported rising CRC incidence rate.^9^ The transition towards a Western lifestyle, typified by diets rich in processed foods, reduced physical activity, and a rise in obesity, has been implicated as a significant driver for the increased incidence of CRC in countries experiencing economic growth.^10^ Conversely, in affluent nations across North America, Europe, and Oceania, incidence rates depict a complex pattern, ranging from declining to stable, and even increasing in some instances.^11^ An exploration into the demographics of CRC reveals that men are disproportionately affected compared to women, with a higher rate of incidence and mortality beyond 50 ages.^12^ Moreover, within the United States, African Americans bear the highest burden of CRC, whereas Asian or Pacific Islanders have the lowest rates of both incidence and mortality.^13^ Genetic variations have been proposed to contribute to these racial and gender disparities, with genome-wide association studies (GWAS) uncovering distinct single-nucleotide polymorphisms (SNPs) linked to CRC risk in diverse populations.^14^ It is evident that differences in exposure to modifiable risk factors, coupled with access to healthcare services, are pivotal in shaping the CRC landscape. As such, tailored strategies that address these disparities are imperative for improving CRC outcomes on a global scale.

## Initiation and development of CRC

**Fig. 1:**
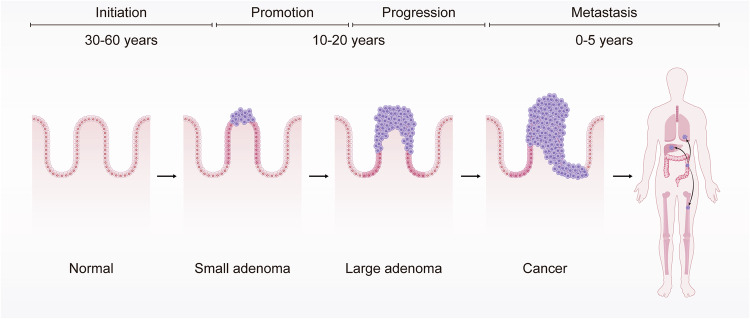
Initiation and development of colorectal carcinogenesis. This Figure illustrates the initiation and development of colorectal carcinogenesis over various timeframes. It begins with a normal colonic epithelium, which can transform into a small adenoma over 30–60 years (initiation phase). This can progress to a large adenoma (promotion phase) and further develop into cancer (progression phase) within 10–20 years. Finally, the cancer can metastasize to other parts of the body within 0–5 years (metastasis phase)

The etiological trajectory of CRC is a multi-stage process delineated by four critical junctures: initiation, promotion, progression, and metastasis (Fig. 1). The initiation phase is characterized by irreversible genetic alterations, such as DNA adduct formation during the chemical carcinogenesis. DNA adducts are prevalently observed in the domain of chemical carcinogenesis. This process commences with the covalent attachment of carcinogens or their active metabolites to DNA, culminating in the creation of DNA adducts. Studies have evidenced such formations in the human colonic mucosa.^15^ These adducts can either be excised through DNA repair mechanisms or eliminated via cellular apoptosis. Nonetheless, they maintain equilibrium levels within tissues targeted by carcinogens, indicative of a balance among carcinogen exposure, adduct formation, and their removal. The persistence of DNA adducts can lead to mutations, which may initiate cancerous developments. Such mutations, particularly in genes governing cell proliferation, can lead to the emergence of small benign neoplasms (adenomas), potentially progressing to malignant states (carcinomas).^16^ During the promotion phase, these genetically altered cells undergo proliferation, forming neoplasms. Progression follows, wherein additional genetic and epigenetic changes enhance the neoplasm’s malignancy, endowing cells with invasive and metastatic capabilities. In the final metastasis stage, cancerous cells disseminate from the primary tumor to distant sites via hematogenous or lymphatic routes. The temporal span of these stages varies widely, with the entire process often unfolding over several decades^17^ (Fig. 1). Hereditary forms of CRC may accelerate through these stages more swiftly, as discussed in subsequent sections.

Three primary genetic and epigenetic disruptions are implicated in CRC carcinogenesis: chromosomal instability (CIN), the CpG island methylator phenotype (CIMP), and microsatellite instability (MSI). CIN involves an array of chromosomal copy number and structural anomalies, potentially stemming from mitotic errors, including those related to mitotic checkpoint proteins and centrosome duplication.^18^ CIMP is an epigenetic phenomenon involving extensive methylation at CpG islands within promoter regions of tumor suppressor genes, leading to transcriptional silencing. The genesis of CIMP is not entirely understood, and there is a lack of uniformity in the markers and criteria delineating CIMP subtypes.^19^ MSI is due to the accumulation of errors in microsatellite regions within the genome, typically owing to the loss of function in DNA mismatch repair genes, such as MLH1, often through promoter hypermethylation. While these molecular phenotypes are distinct, they frequently coexist within CRC pathogenesis; for instance, CIMP and MSI are often interlinked, as CpG island hypermethylation can inactivate mismatch repair genes, leading to MSI.^20^ Within sporadic CRC cases, these aberrations present with varying frequency: CIN in about 85%, CIMP positivity in roughly 20%, and MSI in approximately 15%.^19^ The CIMP has been associated with specific clinical and pathological features of CRC, including response to chemotherapy. Notably, CIMP status has been correlated with a favorable response to certain chemotherapeutic agents, underlining the relevance of epigenetic modifications in treatment stratification.^21^

A salient aspect of CRC development is the emergence of benign precursor lesions known as polyps, which manifest as protrusions in the large intestine’s lining. These visible intermediate lesions are amenable to removal during screening endoscopies. The transition from such lesions to CRC is generally protracted, spanning at least a decade,^22^ offering a critical interval for secondary prevention.

Adenomatous and serrated polyps represent the two principal precursors to CRC. Adenomatous polyps are traditional forerunners, with an estimated 85–90% of sporadic CRCs originating from them.^23^ However, the probability of an adenoma progressing to CRC is less than 10%. Advanced adenomas—characterized by size (≥1 cm), villous histology, or high-grade dysplasia—carry a markedly higher risk of malignant transformation, especially when multiple adenomas are present. The likelihood of cancer development from advanced adenomas escalates with the patient’s age at detection.^24^

Serrated polyps, encompassing hyperplastic polyps, traditional serrated adenomas, sessile serrated adenomas, and mixed polyps, account for 10–15% of sporadic CRCs.^25^ Hyperplastic polyps, the most common among them, were once deemed non-premalignant but are now recognized to harbor malignant potential, particularly when large or located in the proximal colon. A Danish nationwide study revealed odds ratios for CRC of 1.79 for traditional serrated adenomas, 3.40 for sessile serrated adenomas, and 2.50 for conventional adenomas, as compared to individuals with no polyp history.^26^

## Signaling mechanisms underpinning colorectal cancer pathogenesis

**Fig. 2:**
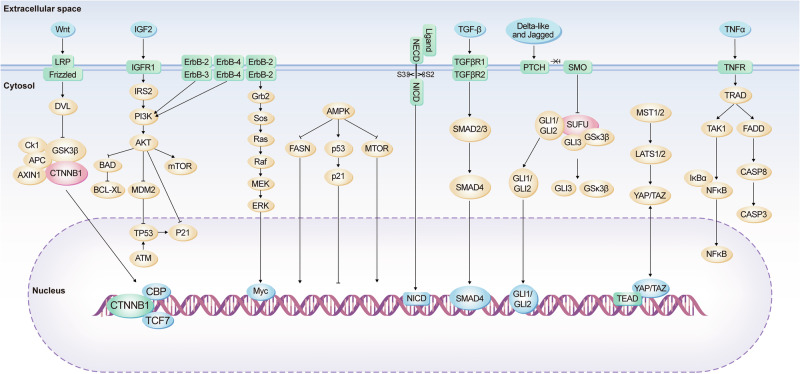
Schematic overview of the diverse signaling cascades implicated in colorectal carcinogenesis. This panel showcases various pathways, including the Wnt, IGF2, ErbB, TGF-β, Notch, Hedgehog, and TNFα pathways. It details how extracellular signals are transmitted through receptors and intracellular molecules to the nucleus, emphasizing the complexity and interconnectedness of these signaling networks in the development and progression of colorectal cancer

CRC is driven by the dysregulation of several key signaling pathways that collectively contribute to the hallmark capabilities acquired during tumorigenesis. In addition to the frequently implicated Wnt/β-catenin signaling axis, the MAPK/ERK pathway emerges as a pivotal route for signal transduction that influences cellular proliferation and differentiation.^27^ Mutations in key components of this pathway, such as KRAS and BRAF, are well-documented in CRC and represent important biomarkers for diagnosis and targeted treatment^28^ (Fig. 2).

The PI3K/AKT/mTOR cascade is another central signaling network that, when aberrant, leads to enhanced cellular growth, survival, and metabolism, thus providing a proliferative advantage to cancer cells. The TGF-β pathway, with its multifaceted roles in cell growth and differentiation, exhibits a context-dependent function in CRC. It serves as a tumor suppressor in early neoplastic events but can pivotally switch to promote epithelial-to-mesenchymal transition (EMT) and metastasis in later stages of the disease.^29^

The JAK/STAT signaling pathway, which is often activated in response to cytokines and growth factors, has a significant role in inflammation-associated CRC, influencing the TME, angiogenesis, and immune escape mechanisms. Notch signaling, which intricately regulates cell fate decisions, is another contributor when deregulated, affecting cell proliferation, stem cell maintenance, and apoptosis.^30^

These pathways do not operate in isolation but are part of a complex and interwoven network of signaling events. Crosstalk between pathways can further complicate the cellular response and the development of effective therapeutic strategies. Understanding these interrelationships is crucial for the development of multi-targeted approaches in the treatment of CRC, which may improve the efficacy of existing therapies and contribute to the discovery of novel therapeutic agents.^3^

### Wnt pathway dysregulation in colorectal cancer

Enhanced Wnt signaling is a key driver of CRC development and progression. The Wnt pathway bifurcates into canonical and non-canonical branches, each with distinct cellular mechanisms and roles. In the canonical branch, ligand-receptor interactions between Wnt proteins and the receptor complex, composed of LRP-5/6 and frizzled, lead to Disheveled (DVL) activation. Subsequent recruitment and inhibition of the destruction complex components, which includes Axin, GSK-3β, CK1, and APC, results in the stabilization of β-catenin by preventing its phosphorylation.^31^ The stabilized β-catenin accumulates in the cytoplasm and subsequently translocates to the nucleus, where it forms a transcriptional complex with TCF/LEF and auxiliary coactivators such as Pygo and Bcl-9. This complex drives the expression of Wnt-responsive genes, including c-Myc and cyclin D1, which are pivotal in cell proliferation and CRC progression^32^ (Fig. 2).

In the realm of non-canonical signaling, the planar cell polarity (PCP) pathway is initiated by Wnt-Frizzled engagement, which orchestrates cytoskeletal dynamics via small GTPases like RhoA. Activated RhoA in turn stimulates downstream effectors including ROCK and myosin, leading to actin reorganization.^33^ Frizzled-10, which is up-regulated in primary colorectal cancer, acts as a positive regulator of the WNT-β-catenin-TCF signaling pathway.^34^ Similarly, the Wnt/Ca^2+^ pathway, often triggered by Wnt5a, involves G-protein activation and the resultant flux of Ca^2+^ ions into the cytoplasm, which has implications for cellular differentiation and can also negatively regulate canonical Wnt signaling through the phosphorylation of TCF/LEF by molecules such as CaMKII.^35^ The secretion of Wnt5a, stimulated by the extracellular calcium-sensing receptor, inhibits defective Wnt signaling in colon cancer cells. Wnt5a suppresses colon cancer by inhibiting cell proliferation and epithelial–mesenchymal transition^36^ (Fig. 2).

Cross-talk between Wnt signaling and other pathways is also a critical aspect of CRC pathogenesis. For instance, the Hippo pathway effector YAP is transcriptionally regulated by the β-catenin/TCF4 complex in CRC cells, highlighting a synergistic interaction that can influence cellular growth and apoptosis.^37^ Moreover, an intricate relationship exists between the Notch and Wnt pathways, as evidenced by the ability of Notch to modulate Wnt signaling, a finding initially observed in Drosophila models. The APC mutation not only perturbs Wnt signaling but also activates the Notch pathway, which is essential for early tumorigenesis in colonic lesion models. Additionally, there is an interplay between the Wnt and Ras pathways, with APC mutations contributing to the stabilization of Ras, thereby enhancing its oncogenic potential through altered proteasomal degradation.^38^

### Elevated PI3K/Akt signaling in colorectal cancer: therapeutic horizons and metabolic intersections

The PI3K/Akt signaling axis exhibits pronounced activation in CRC, presenting a strategic target for interventions aimed at achieving clinical remission. The relationship between this pathway and glucose metabolism in CRC is particularly noteworthy. The enzyme class I PI3K catalyzes the conversion of phosphatidylinositol-4,5-bisphosphate (PIP2) to phosphatidylinositol-3,4,5-trisphosphate (PIP3), subsequently activating Akt kinase through phosphorylation, which then triggers downstream signaling events^39^ (Fig. 2).

Distinct isoforms of Akt exhibit both overlapping and unique roles in cancer progression. The termination of the PIP3 signal is mediated by lipid phosphatases such as PTEN, PIPP (INPP5J), and INPP4B, which are often found to be altered in various cancers, including CRC.^40^

Moreover, this signaling cascade can also activate nuclear factor kappa-light-chain-enhancer of activated B cells (NF-κB), which in turn promotes cellular survival by inducing the phosphorylation and subsequent degradation of IκB, an NF-κB inhibitor. Consequently, NF-κB translocates to the nucleus, where it fosters survival and angiogenesis, thereby contributing to CRC progression.^41^ The PI3K/Akt/eNOS pathway is implicated in these processes.

The oncogenic potential of PI3K/Akt extends to the phosphorylation of MDM2 at Ser186, which in turn mediates the ubiquitination and degradation of the tumor suppressor p53, a pivotal factor in the cellular response to genotoxic stress, thus promoting cell survival over apoptosis. Previous studies have shown that the AKT-MDM2-p53 signaling pathway significantly affects cell apoptosis and is associated with the development and progression of various cancers, including colorectal cancer.^42^ Thus, targeting AKT with costunolide suppresses the growth of colorectal cancer cells and induces apoptosis both in vitro and in vivo.

The interaction between Fas and its ligand activates the caspase cascade through the Fas-associated death domain, leading to apoptosis. Simultaneously, PI3K/Akt enhances cell survival by inactivating Bad, a proapoptotic Bcl-2 family member, and by upregulating the antiapoptotic proteins Bcl-xl and Bcl2.^43^ Inhibition of the PI3K-Akt signaling pathway enhances the sensitivity of Fas-mediated apoptosis. And the synergistic effect of PI3K/Akt inhibition combined with Fas activation markedly enhances cell death in colon cancer, particularly in cells that have developed resistance to Fas-mediated apoptosis.^44^

Cell cycle regulation within CRC is also under the influence of PI3K/Akt, as evidenced by its suppression of p27Kip1 and p130, key inhibitors of the G1/S cell cycle transition, via the inhibition of forkhead box proteins.^45^ Additionally, the inactivation of glycogen synthase kinase 3 (GSK3) by PI3K/Akt leads to increased levels of cyclin D1 and Myc, which are pivotal in cell cycle progression and proliferation (Fig. 2) and this PI3K/Akt-GSK3 pathway could be targeted by Toosendanin and PP9, a steroidal saponin in CRC.^46^

Emerging evidence illustrates the ability of PI3K/Akt to modulate the Hippo pathway, promoting the phosphorylation of YAP to foster colon cancer cell proliferation.^47^ Additionally, the activation of the mechanistic target of rapamycin (mTOR) by PI3K/Akt propels protein synthesis, influencing cell metabolism and growth. Interactions between the PI3K/Akt pathway and the Bone Morphogenetic Protein (BMP) pathway have also been observed in CRC.^48^

### Elucidating the oncogenic synergy of the erbb receptor tyrosine kinase family in colorectal carcinogenesis

The ErbB family of receptor tyrosine kinases significantly marks its presence on the cellular facade of CRC and breast cancer tissues, impacting a myriad of cellular mechanisms. This cadre includes ErbB1 (EGFR), ErbB2 (HER2), ErbB3 (HER3), and ErbB4 (HER4) (Fig. 2).

Studies have revealed EGFR to be upregulated in 60–80% of colorectal malignancies.^49^ Clinical evaluations frequently utilize immunohistochemistry (IHC) assays to identify patients with CRC expressing EGFR in at least 1% of the tumor cells.^50^ The activation of EGFR sets off a cascade of intracellular signaling, prominently involving the MAPK and PI3K/Akt pathways, which are known to augment cellular proliferation, inhibit apoptosis, and promote angiogenesis.^51^

The overexpression of HER2, found in approximately 47.4% of CRC patients, has been associated with a negative prognosis.^52^ HER2’s activation primarily impacts cellular differentiation, proliferation, and the apoptotic process within CRC cells. Amplification of HER2, which can lead to chemoresistance via the ERK1/2 signaling pathway activation. HER2 also preferentially forms complexes with other ErbB family members, which are integral to oncogenic processes.^53^

HER3, characterized by its impaired kinase activity within the receptor tyrosine kinase family, necessitates dimerization with another ErbB receptor to achieve phosphorylation, typically with the oncogenic HER2.^54^ The HER2/HER3 axis, often co-expressed in tumors, is believed to play a substantial role in CRC cell growth. HER3 expression is linked with distal and lower-grade colon cancers. It has been observed that higher levels of HER3 are inversely associated with various clinical features such as histologic grade, tumor size and depth, TNM stage, lymphatic invasion, lymph node metastasis, and distant metastasis.^55^ Furthermore, there is a notable positive association between HER3 and both HER2 overexpression and gene amplification.^55^ Although higher HER3 levels correlate with improved survival outcomes, the significant expression of both HER2 and HER3 in a considerable number of patients highlights the potential of targeting these receptors as a promising treatment strategy for colorectal cancer.^56^

HER4, which can be activated by ligands like heparin-binding EGF-like growth factor, neuregulins, and betacellulin, has been implicated in promoting cell proliferation and metastasis while inhibiting differentiation through PI3K/Akt and Shc pathway activation.^57^ The inhibition of HER3 or the absence of HER4 leads to increased apoptosis in CRC cells, potentially through a HER3–HER4 heterodimer-dependent Akt pathway.

In CRC cells exhibiting both WNT and Ras mutations, HER4 ectopic expression was found to augment unanchored growth and tumor xenograft formation. The interruption of HER4 expression hindered the WNT-driven growth of CRC cells, suggesting a cooperative oncogenic effect with activated WNT signaling, both in murine and human colon cells. Furthermore, the coexistence of HER4 with active EGFR signaling in human CRC has been correlated with the activation of the Ras-Raf-MEK-ERK pathway, thereby accelerating cancer progression^58^ (Fig. 2).

### Delineating the oncogenic role of notch signaling in colorectal carcinogenesis

The Notch signaling pathway, a pivotal regulator of cellular physiology, is frequently usurped during CRC pathogenesis. Aberrant activation of Notch signaling in CRC results from a myriad of genetic and epigenetic events, including point mutations, amplifications of Notch pathway components, chromosomal translocations, and histone modifications, all contributing to the oncogenic phenotype.^59^ This pathway involves a cadre of four transmembrane receptors—Notch 1, 2, 3, and 4—that interface with numerous ligands, culminating in the modulation of key cellular processes such as proliferation, differentiation, apoptosis, and the sustenance of stem cell niches (Fig. 2).

In the CRC landscape, Notch signaling has emerged as a critical player. It modulates tumor behavior through a series of well-orchestrated steps, starting with the proteolytic cleavage of the Notch intracellular domain (NICD) from its parent receptor by the γ-secretase complex. The free NICD subsequently translocates to the nucleus, where it converges on the DNA-binding protein RBPJ. This liaison activates transcriptional programs that drive the expression of a set of genes, including those encoding the Hairy Enhancer of Split (HES) family of transcriptional repressors, CDKN1A (p21), HES-related proteins (HEY), Notch-regulated ankyrin repeat protein, and key cell cycle regulators such as cyclins D1/3, c-myc, and HER2^30^ (Fig. 2).

The pathological amplification of Notch signaling in CRC has been implicated in fostering tumorigenic attributes, specifically enhancing cell proliferation, survival, EMT, and angiogenesis.^60^ The upregulation of Notch ligands observed in CRC further amplifies this signaling, reinforcing its role in tumor progression. Moreover, a complex crosstalk exists between Notch and the Ras signaling pathways in CRC; activating mutations within the Ras pathway have been found to elevate Notch signaling, facilitating the pro-oncogenic effects of Ras-driven transformation.^61^

### SMAD signaling dynamics in BMP4 mediated regulation of colorectal cancer progression

BMP4 is a critical signaling molecule within the TGF-β family, known to exert pivotal influence during the embryogenic processes by modulating cellular apoptosis, proliferation, and differentiation.^62^ BMP4’s biological implications extend to oncogenesis, where it has been observed to play a dichotomous role by promoting differentiation in cancer stem cell populations and potentially suppressing the oncogenic progression in colorectal carcinoma^63^ (Fig. 2).

As a secreted glycoprotein, BMP exerts its function through interaction with specific receptors, including BMPR1A (also known as ALK3), BMPR1B (ALK6), and BMPRII. The signaling cascade initiated by BMP involves the phosphorylation of intracellular SMAD proteins—specifically, SMAD1, SMAD5, and SMAD8—which are then translocated into the nucleus in conjunction with SMAD4, a common mediator SMAD, to regulate the transcription of various target genes, among them the inhibitor of DNA binding (ID) proteins and the chemokine CCL15.^64^ Notably, the disruption or downregulation of the BMP pathway has been recognized in a substantial fraction of colorectal cancer specimens, suggesting its potential as a biomarker or therapeutic target^65^ (Fig. 2).

Furthermore, there is accumulating evidence that BMP signaling can exert an antagonistic effect on the Wnt/β-catenin pathway. Specifically, BMP-mediated activation of SMAD4 has been implicated in the repression of Wnt target genes, such as c-myc and Axin2, within microdissected intestinal adenomas derived from tamoxifen-induced murine models. Additionally, the crosstalk between BMP and Wnt signaling pathways has been postulated to involve the PI3K/Akt pathway, indicating a complex network of interactions that may influence cellular fate and tumorigenic potential.^66^

### Hedgehog signaling in colorectal tumor progression

The Hedgehog (Hh) signaling cascade has been increasingly recognized for its oncogenic influences within CRC pathogenesis. Originally identified in the fruit fly Drosophila melanogaster, the Hh pathway is now known to be a central regulator of cell proliferation, differentiation, and embryonic patterning. In mammals, the Hh protein family, including Sonic Hedgehog (SHH), Indian Hedgehog (IHH), and Desert Hedgehog (DHH), is critical for a myriad of cellular processes such as survival, proliferation, apoptosis, differentiation, migration, and invasion^67^ (Fig. 2).

Upon binding of a Hh ligand to its receptor Patched-1 (PTCH1), an inhibitory effect on the transmembrane protein Smoothened (SMO) is lifted. This relief of inhibition permits the activation of the intracellular signaling cascade, culminating in the nuclear translocation of the GLI family of zinc-finger transcription factors, notably GLI1 and GLI2 (Fig. 2). Within the nucleus, these transcription factors orchestrate the expression of various genes that govern cell fate, including the regulators of angiogenesis such as platelet-derived growth factor (PDGF) and the EMT modulator SNAIL.^68^

The perturbation of Hh signaling has been implicated in several solid tumor types, with particular prominence in basal cell carcinoma of the skin and medulloblastoma, underscoring its oncogenic potential. Within the colonic epithelium, Hh signaling not only contributes to tissue homeostasis but also to repair mechanisms, and its aberrant activation has been documented in the milieu of CRC.^69^ Indeed, various research efforts have denoted the overexpression of Hh pathway constituents including the SHH ligand, the PTCH1 receptor, and the SMO receptor in a spectrum ranging from hyperplastic polyps to adenocarcinomas of the colon.^70^ Experimental manipulation through the administration of exogenous SHH has been shown to augment the proliferation of colonic cells in murine primary culture models, thereby suggesting that Hh-mediated signaling may foster CRC pathogenesis. Furthermore, investigations have revealed a marked elevation of Shh mRNA in CRC tissues compared to their normal colonic counterparts, reinforcing the notion of its contributory role in colorectal tumorigenesis.^71^

### Delineating the role of the hippo signaling pathway in colorectal cancer pathogenesis and progression

The Hippo signaling cascade plays an instrumental role in a myriad of cellular processes, including the regulation of stem cell characteristics such as proliferation, morphology, and survival, as well as their migratory behavior, self-renewal capacity, and overall maintenance of tissue equilibrium and determination of organ size. The pertinence of this pathway has been underscored by a growing body of literature. Furthermore, the Hippo pathway has emerged as a critical player in the context of oncogenesis, credited with tumor-suppressive functions that are mediated through various components like the fat storage-inducing transmembrane protein, the serine/threonine-protein kinases LATS1/2, the MST1/2 kinases, the transcriptional co-activators TAZ and YAP1, and the TEAD family of transcription factors^72^ (Fig. 2).

To elucidate the molecular mechanics, the Hippo pathway is activated via the MST kinases that are themselves stimulated by the FAT transmembrane proteins. Activated MST kinases then phosphorylate and activate LATS kinases, which in turn phosphorylate YAP and TAZ, restraining their translocation into the nucleus. This phosphorylation event reduces the interaction potential between YAP/TAZ and TEAD transcription factors, thereby diminishing the transcriptional activation of pro-oncogenic genes such as those encoding β-catenin, k-ras, and components of the Akt/mTOR signaling axis that are implicated in the incipience and progression of colorectal tumorigenesis.^73^

In the setting of CRC, the suppressive regulation through the Hippo pathway is often compromised, leading to elevated levels of YAP, which in turn propel the migratory and invasive behaviors, as well as the proliferative and EMT phenotypes of colon cancer cells. TAZ has been identified as a molecular check against the phosphorylation of Dvl by Wnt3a, impeding the interaction between Ck1δ/ε and Dvl, and consequently, the Wnt/β-catenin signaling cascade is downregulated.^74^ Additionally, perturbations in MST and LATS kinases further impede the recruitment of TAZ to the cell membrane, curtailing the pathophysiological influence of Wnt3a.

At the transcriptional level, the YAP1/KLF5 complex has been shown to engage the promoter region of Ascl2, a key Wnt signaling target gene, thereby amplifying the expression of Ascl2 and enhancing the self-renewal potential of CRC progenitor cells.^75^ Conversely, inhibition of YAP in colon-derived cell lines markedly dampens the signaling outputs of both Notch and Wnt pathways, subsequently diminishing cell proliferation and viability. Cells deficient in Mst1/2 exhibit pronounced upregulation of key Notch pathway genes, including Hes1 and Hey1.^76^ Overexpression of YAP has also been linked to aberrant activation of the Notch pathway, contributing to the inhibition of cellular differentiation.^77^

### Regulatory dynamics of AMP-activated protein kinase in colorectal carcinogenesis: energy sensing and beyond

AMP-activated protein kinase (AMPK) is recognized as a pivotal enzyme in cellular energy homeostasis, governed by the AMP/ATP ratio, and plays a critical role in moderating a spectrum of cellular functions, including survival, proliferation, differentiation, migration, and metabolic modulation in CRC cells.^78^

At the biochemical level, AMPK’s influence is primarily mediated by its interaction with key metabolic pathways, including oxidative phosphorylation and various other signal transduction cascades. Activation of AMPK has been shown to engage with tumor suppressor p53, triggering both autophagic and apoptotic pathways, while concurrently modulating cell cycle progression. Through phosphorylation, activated AMPK has been found to attenuate mTOR activity (Fig. 2), thereby suppressing cellular growth and protein synthesis via the TSC1/TSC2 complex,^79^ the inhibition of Rag GTPases,^80^ and the induction of REDD1 expression.^81^ Moreover, AMPK has been implicated in the downregulation of oncogenic receptor tyrosine kinase pathways, including ErbB2 and EGFR, with subsequent repercussions on mTOR and ERK signaling pathways.^82^ In its inactivated state, AMPK can exert an inhibitory effect on IRS1, a key component of the IGF1/insulin axis, and thereby attenuate the PI3K/Akt/mTOR signaling cascade, which is integral to oncogenic processes.^83^ The pharmacological activation of AMPK, such as through metformin treatment, has been associated with the recruitment of anti-angiogenic and anti-inflammatory mediators like IL-1β, TNFα, IL-6, NF-κB, and HIF-1α, which collectively attenuate the angiogenic influence of VEGF.^84^ As an upstream effector of the Hippo signaling pathway, AMPK is also known to induce phosphorylation of the transcriptional coactivator YAP, thereby inhibiting its oncogenic functions in colon cancer cells, which include proliferation and evasion of apoptosis, as well as glucose uptake and glycolytic activity.^85^

Conversely, AMPK exhibits metabolic effects that facilitate oncogenic transformation, such as promoting a lipogenic phenotype. The enzymatic activity of AMPK leads to the phosphorylation of key lipogenic enzymes such as ACC-1, culminating in the suppression of FASN, SREBP-1c, and SCD-1 levels, which are instrumental in the biosynthesis of lipids crucial for the proliferative demands of cancer cells. Additionally, the Warburg effect, a characteristic alteration of cancer metabolism, predominantly affects glucose metabolism but also extends its influence to amino acid and lipid metabolism.^86^ In addition, resveratrol can mitigate the Warburg effect by activating AMPK in CRC models.^87^ This observation underscores that while AMPK is a central regulator of metabolic pathways, the precise contribution of the Warburg effect and its modulation by other signaling pathways, independent of AMPK, warrants further investigation to delineate their roles in cancer metabolism.^87^

### Interplay of MAPK, PI3K/AKT, and JNK signaling pathways in colorectal cancer progression and therapeutic resistance

These pathways do not operate in isolation but are part of a complex and interwoven network of signaling events. Crosstalk between pathways can further complicate the cellular response and the development of effective therapeutic strategies. Understanding these interrelationships is crucial for the development of multi-targeted approaches in the treatment of CRC, which may improve the efficacy of existing therapies and contribute to the discovery of novel therapeutic agents.^3^

Deregulation of the Ras/Raf/MEK/MAPK/ERK signaling pathway is a critical factor that drives the progression of CRC. This pathway serves as a pivotal conduit for signals that regulate cell proliferation and the cell cycle. Within the spectrum of tumors, somatic mutations in the RAS gene are present in approximately 30% of cases, leading to the activation of a cascade that includes RAF, MEK, and MAPK/ERK. These mutations instigate a domino effect starting with RAS activation, which then activates RAF, followed by the phosphorylation of MEK, culminating in the activation of MAPK/ERK. This sequential activation propels cell cycle progression and proliferation, hallmark features of cancerous transformation.^28^

In the context of CRC, somatic mutations within the MAPK pathway typically serve as activators for carcinogenesis. For instance, certain compounds, such as Ganoderma lucidum polysaccharide, have been shown to trigger apoptosis in CRC cells by upregulating JNK through the MAPK pathway, implicating a role for mitochondrial pathways and MAPK in cell death. Conversely, somatic mutations in PIK3CA can dampen the sensitivity of CRC cells to MEK inhibitors, while somatic mutations in PTEN might confer total resistance.^88^ Interestingly, a synergy in inhibiting both the PI3K/AKT and RAF/MEK/ERK pathways has been suggested to thwart the downstream mTOR pathway effectively^58^. Furthermore, the Wnt signaling pathway has been identified as a potential mediator of resistance to MEK inhibitors in cancers harboring somatic BRAF mutations, potentially driven by factors such as CEMIP. Additionally, it has been observed that Ras signaling can interact with the AKT and Wnt pathways in CRC, with certain inhibitors showing an ability to slightly attenuate these pathways in CRC cells containing mutant k-Ras.^89^

Apart from the Ras pathway, the JNK signaling pathway displays a dual role in CRC progression. The proto-oncoprotein c-Jun, part of the AP-1 transcription factor, is overactivated in various cancers and is commonly activated via phosphorylation by JNKs. This phosphorylation facilitates the formation of a complex with TCF4 and β-catenin, which may enhance transcription by recruiting β-catenin to the transcription initiation site on AP-1 elements. In certain mouse models of CRC, inhibiting phosphorylated c-Jun or colon-specific c-Jun inactivation has shown to reduce tumor burden and extend lifespan.^90^ In addition, JNK1 has been identified as an upstream regulator of Stat3 and has been implicated in other anticancer mechanisms, including the inhibition of centrosomal amplification.^91^

Interestingly, there is evidence suggesting that the Hippo pathway may be influenced by JNK signaling. For instance, JNK activation can lead to the nuclear translocation of Yki, a component of the Hippo pathway, thereby promoting cell proliferation.^92^ JNK has also been implicated in the activation of YAP1 in response to DNA damage and may facilitate the inhibition of Hippo pathway kinases through interactions with molecules like Ajuba.^93^ While the connections between JNK and the Hippo pathway in mammalian colon cancer remain to be fully elucidated, their interplay in CRC tumorigenesis is a subject of ongoing research.

### Inflammation and signaling in CRC pathogenesis

The association between persistent inflammation and the emergence of neoplastic conditions is a well-established paradigm in cancer biology. Chronic inflammatory states, whether elicited by infectious agents, immune dysregulation, or environmental insults such as tobacco smoke, airborne pollutants, or dietary components, are known to significantly elevate the risk of neoplastic transformation.^94^ In CRC specifically, conditions such as entrenched inflammatory bowel disease (IBD) and sustained gastrointestinal inflammation, often exacerbated by dietary patterns emblematic of a western diet, stand out as primary risk factors. While a minority of CRC cases, approximately 5%, arise against a backdrop of explicit chronic inflammation, animal models, such as the azoxymethane/dextran sulfate sodium (AOM/DSS) model, have yielded valuable insights into the diverse mechanistic underpinnings of tumorigenesis that also appear pertinent to sporadic CRC cases.^95^

Tumorigenesis necessitates two pivotal events: an initiating event characterized by an accumulation of genetic or epigenetic alterations leading to the suppression of tumor suppressor genes or the activation of oncogenes, and a promotion event involving the clonal expansion of cells harboring such mutations, culminating in the development of an overt tumor. Inflammation significantly contributes to both these foundational events.^96^

Inflammatory processes can instigate tumorigenesis through DNA damage even in the absence of external carcinogens. This phenomenon can be partially ascribed to amplified oxidative stress, induced by resident or recruited innate immune cells, such as macrophages and neutrophils, which discharge elevated levels of reactive oxygen and nitrogen species (RONS) into the inflamed tissue milieu. These reactive species can inflict various forms of DNA damage on intestinal epithelial cells (IECs), ranging from single and double-strand breaks to nucleotide alterations and the creation of abasic sites. Indeed, the augmented output of reactive oxygen species by myeloid lineage cells can precipitate genome-wide DNA mutations and directly transform IECs, thus initiating tumorigenesis in the context of chronic intestinal inflammation, even in the absence of carcinogenic interventions. Moreover, inflammatory conditions within the intestine can compromise epithelial barrier integrity, potentially exposing the intestinal stem cell niche to environmental mutagens or positioning stem cells proximal to inflammatory cells that secrete genotoxic substances.^97^

Furthermore, the disintegration of the intestinal barrier can facilitate the incursion of commensal and pathogenic microorganisms, leading to interactions between IECs and microbial entities with potential pro-tumorigenic attributes. Chronic inflammation in the intestine prompts excessive tissue regeneration, stimulating the proliferation and clonal expansion of cells carrying tumorigenic initiations (promoting tumor development), and can also induce the dedifferentiation of mature cells into stem-like cells to accommodate the regeneration of the damaged tissue. Intestinal stem cells are inherently more resilient to replication stress and DNA damage, and dedifferentiating cells have been shown to gain the capacity to initiate tumors. Thus, inflammation can heighten the mutational load and increase the pool of cells with potential to initiate tumors. Inflammatory processes also modulate critical cytokine receptor-mediated signaling pathways that govern key tumor-initiating and promoting functions in CRC, such as NF-κB activation via TNF receptor and IL-1 receptor signaling, as well as STAT3 activation through IL-6 and IL-11 induced signaling^98^ (Fig. 2). Contrastingly, IL-22, another cytokine that activates STAT3, can induce the transcription of DNA damage response genes, thereby mitigating the genotoxic effects induced by inflammation. It’s noteworthy that in inflammation-driven CRC, TP53 mutations, which augment TNF, NF-κB, and STAT3 signaling, appear early in the carcinogenic process. Other somatic mutations identified under inflammatory conditions, such as those in NFKBIZ, ZC3H12A, TRAF3IP2, and HNRNPF, are less common in CRC.^99^

In addition to provoking mutations via DNA damage, inflammation can also influence cancer-related genes through epigenetic mechanisms that lead to the silencing of crucial tumor suppressor genes. Cytokines such as IL-1β, IL-6, and TNF regulate the expression of DNA methyltransferases DNMT1 and DNMT3B, leading to alterations in gene methylation and expression patterns in pathways implicated in CRC, including those involving NOTCH or p53 signaling.^100^ Furthermore, NOTCH signaling has been implicated in driving metastasis in KRAS-driven CRC via TGF-β-mediated neutrophil recruitment and in promoting invasion and metastasis in a CMS4 tumor model.^61^ Inflammation-related RONS and cytokines are parallel mechanisms that mutually reinforce each other. It has been observed that RONS can escalate the release of inflammatory cytokines, which in turn may promote further production of RONS.^101^ These interactions are instrumental not only in tumor initiation, as discussed earlier, but also adversely affect treatment outcomes, particularly in the context of immunotherapy. Recent studies, including our own, have demonstrated that inflammation negatively impacts treatment efficacy in CRC and other malignancies, such as intrahepatic cholangiocarcinoma (ICC).^102^ This underscores the importance of addressing inflammatory pathways to enhance therapeutic responses. For example, Prostanoids, lipid mediators synthesized during inflammation via COX1 and COX2 pathways, significantly influence all stages of colorectal tumorigenesis. Prostaglandin E2 (PGE2) emerges as a predominant prostanoid in CRC, acting as a critical inflammatory mediator.^103^ Given its significant role, targeting PGE2 presents a promising therapeutic strategy for colon cancer treatment. For example, the aqueous extract of Salvia miltiorrhiza Bunge demonstrates potential in attenuating tumor-associated macrophage infiltration and enhancing the efficacy of anti-PD-L1 immunotherapy in CRC.^104^ This effect is mediated through the modulation of the Cox2/PGE2 cascade. The role of inflammation in tumorigenesis is a topic of substantial significance in the field of cancer immunology. The crosstalk between inflammation and cancer is mediated by a complex network of cytokines, chemokines, and cellular interactions that collectively contribute to the TME and tumor progression.^105^ The TME is not merely a passive recipient of transformed cells but an active participant in the process of carcinogenesis.

## Genetic foundations of CRC signaling pathways

### Subtypes of colorectal cancer: sporadic and familial classifications

Colorectal cancer can be stratified into two principal types: sporadic and familial. The demarcation between these two forms is a topic of considerable discussion within the oncological community. It is widely accepted that about 35% of CRC cases is highly relevant to the increased family burden. For instance, in the Sweden populations, studies have found that between 11–13% of individuals diagnosed with CRC have a first-degree relative with the same condition.^106^ Data from Australia suggest this familial connection exists in 16% of cases.^107^ The proportion attributed to familial CRC increases when considering more distant relatives, such as second- or third-degree kin.

**Fig. 3:**
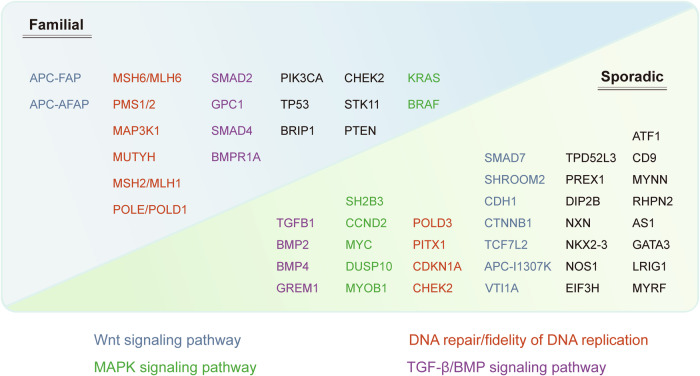
Comprehensive schematic depicting the genetic architecture and heritability of colorectal cancer. This schematic illustrates the genetic factors involved in familial and sporadic colorectal cancer. It categorizes genes based on their association with specific signaling pathways: Wnt signaling pathway (blue), MAPK signaling pathway (green), DNA repair/fidelity of DNA replication (red), and TGF-β/BMP signaling pathway (purple). Familial cases include genes like APC-FAP, MSH6/MLH6, and SMAD2, while sporadic cases involve genes such as ATF1, SMAD7, and TPD52L3. This visual aids in understanding the complex genetic landscape contributing to colorectal cancer

CRC demonstrated moderate heritability, with odds ratios comparable to those observed in lung and kidney cancers. Meanwhile, the absence of a cancer history in a so-called ‘sporadic’ case does not necessarily rule out a genetic component. Various factors, including reduced family size, adoptions, and incomplete family health records, could obscure a true hereditary risk.^108^ This blurring of lines between sporadic and familial CRC necessitates a reevaluation of how these cases are classified^109^ (Fig. 3).

CRC cases stemming from high-penetrance alleles form only a small fraction of the total number of cases. Syndromes such as Lynch syndrome and familial adenomatous polyposis (FAP), which involve high-penetrance mutations, likely account for less than 5% of all CRC cases. Conversely, the majority of familial CRC may be ascribed to the presence of lower-penetrance alleles.^110^ This background sets the stage for an in-depth analysis of CRCs associated with high-penetrance mutations characteristic of syndromes like FAP and Lynch syndrome, as well as the hamartomatous polyposis syndromes. The following sections will also highlight the emerging significance of low-penetrance mutations and modifier genes in the etiology of CRC. These genetic variations, while individually contributing a modest risk, may collectively exert a substantial influence on cancer susceptibility and present a complex landscape of genetic interactions that modulate CRC risk (Fig. 3).

### The APC gene and its role in the etiology of familial adenomatous polyposis

FAP is a hereditary disorder characterized by the proliferation of numerous adenomatous polyps in the colorectal region. These polyps typically emerge during the late childhood or adolescent years. Without appropriate medical intervention, there is a near certainty that at least one of these adenomas will progress to adenocarcinoma by the time the individual reaches young adulthood. The likelihood that an individual with FAP will develop colorectal cancer is estimated at approximately 100%. The disorder also frequently presents with various extracolonic symptoms including multiple polyps and ligamentoid fibromatosis. The prevalence of FAP in the population is reported to be between 1 in 10,000 and 1 in 8000.^111^ Although it is often cited that FAP accounts for about 1% of all CRC cases, a more precise calculation based on a general lifetime CRC risk of 1 in 20 suggests that FAP may actually represent only about 0.2% of CRC occurrences.^112^

Central to the pathogenesis of FAP are mutations in the APC gene, a tumor suppressor gene of considerable significance. The presence of a germline mutation in the APC gene sets the stage for the development of FAP, aligning with Knudson’s two-hit hypothesis. The rapid formation of thousands of adenomas within a span of 15 to 40 years indicates that two genetic ‘hits’ are sufficient for the initiation of tumorigenesis. However, the observation that only a minority of these adenomas progress to full-blown cancer suggests the necessity of additional genetic alterations. The sequence of events leading to tumorigenesis has been extensively documented in the literature.^113^

The APC gene encodes a protein of 2843 amino acids with multiple functional domains. Over 95% of mutations in the germline APC gene are truncating mutations, such as nonsense mutations, insertions, or deletions, which typically lead to a frame shift in the reading sequence.^114^ These germline mutations are cataloged in comprehensive databases such as the Human Genetic Disease Database and The APC database, both of which provide valuable resources for researchers and clinicians.

Notably, certain germline mutations within the APC gene correlate with distinct clinical manifestations. For instance, the classic presentation of FAP, characterized by the development of thousands of adenomas, is often associated with mutations occurring between codons 169 and 1600.^115^ Mutations between codons 463 and 1,387 are frequently observed in patients who exhibit congenital hypertrophy of the retinal pigment epithelium, a condition sometimes seen in FAP.^116^ Additionally, Gardner’s syndrome—a variant of FAP marked by osteomas, skin fibromas, and epidermoid cysts along with polyposis—is typically linked to mutations in a relatively narrow region of the APC gene, between codons 1403 and 1578.^117^

An attenuated form of FAP is also recognized, characterized by a significantly reduced number of adenomas, ranging from a few dozen to several hundred. Some instances of this attenuated form are attributed to splice-site mutations leading to in-frame deletions that result in the synthesis of nearly full-length, albeit hypomorphic, APC proteins. This can account for the milder phenotype observed in these cases. Most instances of attenuated FAP, however, are caused by mutations in the gene’s 5’ region, upstream of codon 157. These mutations, although truncating, paradoxically lead to a less severe phenotype due to an alternative translation initiation site at codon 184, facilitated by an internal ribosome entry site, resulting in a truncated protein lacking key functional domains.^118^

Furthermore, in about 20% of FAP cases, no mutations in the APC gene are detected using conventional diagnostic methods. Combination of denaturing gradient gel electrophoresis (DGGE) and protein truncation test (PTT) is a common method to detect the presence of somatic cell Mosaic.^119^ Research into these cases has revealed a phenomenon of allele-specific loss of expression. In certain families, a reduction of approximately 50% in transcript levels from one APC allele has been observed, with no detectable genomic sequence alterations to explain this reduction.^120^ Three different germinal mutation primarily consisting of deep intronic variants lead to the formation of pseudoexons, subsequently activating cryptic splice sites, resulting in reduced APC expression.^121^ The hereditary nature of this loss of expression and its association with the FAP phenotype has been demonstrated, suggesting that a 50% reduction in expression constitutes a first ‘hit’, followed by a complete loss from the second allele, leading to a significant reduction in functional APC protein and the onset of disease.

After loss-of-function mutation of APC, Wnt/β-catenin signaling is activated and accompanied by the disruption of axis inhibition protein (Axin). For instance, mutations in the AXIN2 gene, which plays a role in the WNT signaling pathway, have been identified in families with dominantly inherited tooth agenesis and CRC.^122^ Based on this, AlkB homolog 5 (ALKBH5), an RNA N6-methyladenosine eraser was found to be the target of Axin, and could be combined with programmed cell death protein 1 (PD-1) treatment after being coated with vesicle to slow down CRC progression.^123^

From a diagnostic standpoint, these findings have enabled the molecular diagnosis of nearly all FAP cases. Despite this, not all clinicians routinely determine the specific APC mutation in patients with FAP, as the diagnosis can be clearly established through sigmoidoscopy and the risk of cancer development is not influenced by knowledge of the mutation. However, genetic testing holds particular importance for adolescents and young adults with a family history of FAP, allowing for targeted surveillance measures for those truly at risk.^124^

### Lynch syndrome: intersecting MMR deficiency and signaling pathway dysregulation

The term “hereditary non-polyposis colorectal cancer” (HNPCC) historically referred to a familial predisposition primarily to CRC, without the formation of numerous polyps. However, this terminology fails to encompass the broad spectrum of malignancies associated with the condition, including, but not limited to, malignancies of the endometrium, stomach, ovaries, small intestine, hepatobiliary tract, uroepithelium, and central nervous system. Consequently, a revised nomenclature, Lynch syndrome, has been adopted, honoring Dr. Henry Lynch for his pioneering contributions to the elucidation of the disease’s hereditary nature.^125^

Lynch syndrome is caused by germline mutations in the DNA mismatch repair (MMR) genes, including MLH1, MSH2, MSH6, and PMS2. The risk of developing colorectal cancer in carriers of these mutations is estimated to be approximately 50% despite invasive treatment or regular screening, with a considerably lower risk for other associated malignancies.^126^

The prevalence of Lynch syndrome among all CRC cases hinges on the criteria employed for defining the syndrome. Traditional definitions, which rely on family history and age at disease onset, suggest that Lynch syndrome may account for about 5% of CRC cases.^127^ However, more rigorous methodologies, such as molecular, histological, and immunohistochemical analyses, suggest that when Lynch syndrome is strictly defined by the presence of a germline MMR mutation, the percentage drops to approximately 2.5%.^128^ The variability in the estimated prevalence across studies may be attributable to differences in genetic or environmental factors or the methodologies employed. The classification of familial CRC cases without identifiable MMR gene mutations as Lynch syndrome remains contentious.

With respect to the molecular consequences of MMR heterozygosity mutations, DNA repair capacity has not been fully compromised. However, the development of cancer in individuals with Lynch syndrome typically follows the two-hit hypothesis, with the second hit being a somatic event such as loss of heterozygosity, mutation, or methylation of promoter CpG islands.^129^ The promoter methylation of systemic Mlh1, a core MMR protein, exhibits jejune-specific MMR haploidy, which affects tissue-specific microsatellite stability to induce gastrointestinal cancer.^130^ Mice with systemic deletion of Msh2 also showed a higher frequency of gene mutations in the cecum and colon.^131^ About one-fifth of cases with MSH2 mutations but no germline mutations are detected have the EPCAM 3’-deletion.^132^ MMR deficiency leads to the single nucleotide variations and MSI, which is a hallmark of Lynch syndrome tumors.^133^

The distribution of mutations within the MMR genes associated with Lynch syndrome is widespread, with no discernible hotspots, necessitating comprehensive screening strategies for mutation detection.^134^ Large deletions, particularly in MSH2, and diverse mutation types, including missense and nonsense mutations, are observed across these genes. The penetrance and expressivity of these mutations do not appear to be influenced by their location within the gene, suggesting that most mutations lead to loss of function.^135^ Interestingly, with the uptake of multigene panel testing, these mutations may be reclassified as benign variants rather than pathogenic mutations.^136^

### MYH mutations and their impact on colorectal cancer predisposition

In the realm of high-penetrance mutations, biallelic mutations in the MutY human homolog (MYH) gene have recently been recognized to predispose individuals to CRC.^137^ MYH, the human homolog of the bacterial metY repair gene, is involved in the base excision repair pathway. The pathogenicity of somatic MYH mutations is thought to primarily arise from the accumulation of G:C → T:A transversions in APC, which results in carriers of monoallelic disease-causing MUTYH strain variants having a higher risk of developing tumors.^138^ A diverse array of somatic MYH mutations, including both nonsense and missense variants, have been identified in CRC patients.^139^ It is noteworthy that some patients exhibit only a single detectable somatic MYH mutation, suggesting the presence of other elusive mutations.^140^ When both alleles of MYH are mutated, the penetrance is very high. However, individuals heterozygous for a somatic MYH mutation may display a polyposis phenotype, implying a potential low penetrance of some somatic MYH mutations, modulated by unidentified genetic or environmental factors.^141^

Two prevalent missense mutations in MYH, Y165C, and G382D, have been identified in diverse populations and represent over half of all MYH mutations in CRC patients.^142^ The prevalence of somatic MYH mutations in the general population appears low, although large-scale control studies are necessary to establish accurate frequencies. The role of somatic MYH mutations in CRC seems to be confined to cases with moderate adenoma counts (10–1000), rather than extensive polyposis (>1000 adenomas).^143^ In situations where an APC mutation is absent, MYH somatic mutations may account for approximately 8–20% of polyposis cases.^144^ It has been suggested that the cancer phenotype associated with MYH mutations may be as prevalent as FAP. Nevertheless, the overall contribution of MYH mutations to CRC is estimated to be less than 1%.^145^

## Epigenetic regulation and signaling pathway dynamics in CRC

### Introduction to epigenetic regulation in CRC

**Fig. 4:**
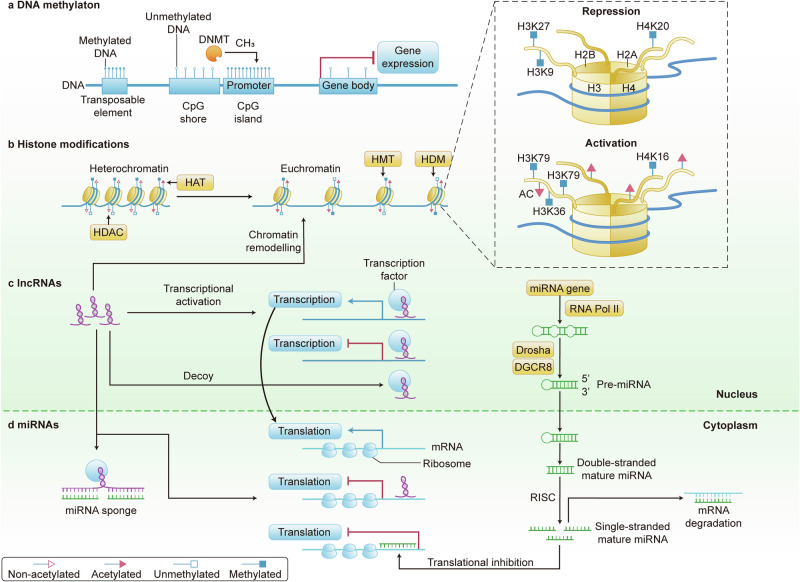
The landscape of epigenetic modulation in colorectal cancer signaling cascades. This figure illustrates the epigenetic mechanisms regulating gene expression in colorectal cancer, including DNA methylation, histone modifications, lncRNAs, and miRNAs. **a** DNA methylation affects gene expression through DNA methyltransferases (DNMT); **b** histone acetyltransferases (HAT) and histone deacetylases (HDAC) regulate chromatin states via specific histone marks (e.g., H3K27, H3K4, H3K9); **c** lncRNAs are involved in transcriptional activation and decoy mechanisms; **d** miRNAs, transcribed by RNA polymerase II and processed by Drosha and DGCR8, inhibit mRNA translation and promote degradation. These mechanisms together form the complex landscape of epigenetic regulation in colorectal cancer

Epigenetics refers to a set of molecular modifications that affect gene expression without altering the underlying DNA sequence. In the context of CRC, epigenetic regulation plays a fundamental role in the initiation, progression, and metastasis of the disease.^146^ It encompasses mechanisms such as DNA methylation, histone modifications, and the activity of non-coding RNAs (ncRNAs), which together orchestrate a complex regulatory network that can either promote or suppress tumorigenesis (Fig. 4).

Colorectal cancer is a multifaceted disease where both genetic and epigenetic aberrations contribute to its heterogeneous nature. Epigenetic alterations in CRC are varied and widespread, affecting a multitude of genes and pathways involved in cell cycle control, apoptosis, DNA repair, and cell adhesion.^147^ These modifications can lead to the inactivation of tumor suppressor genes and the activation of oncogenes, thereby disrupting the delicate balance of cellular homeostasis.

DNA methylation, particularly at CpG islands within promoter regions, is one of the most extensively studied epigenetic mechanisms in CRC. Aberrant hypermethylation can lead to gene silencing and is commonly observed in key tumor suppressor genes such as MLH1, leading to MSI and a distinct molecular subtype of CRC. Conversely, genome-wide hypomethylation in CRC contributes to CIN and an increased mutation rate, which are characteristics of tumor progression.^148^

Histone modifications, including acetylation, methylation, phosphorylation, and ubiquitination, are dynamic processes that modulate chromatin structure and accessibility. In CRC, dysregulated patterns of histone modifications can promote carcinogenesis by altering the expression of genes involved in crucial cellular functions. For instance, the overexpression of histone deacetylases (HDACs) has been observed in CRC and is associated with poor prognosis, highlighting the potential of HDAC inhibitors as therapeutic agents.^149^

NcRNAs, such as microRNAs (miRNAs), long non-coding RNAs (lncRNAs), and circular RNAs (circRNAs), are increasingly recognized for their role in post-transcriptional gene regulation in CRC. These ncRNAs can act as oncogenes or tumor suppressors and have been implicated in various aspects of CRC pathogenesis, including EMT, angiogenesis, and immune evasion.^150^

Understanding the epigenetic landscape of CRC has significant clinical implications. Epigenetic biomarkers may serve as diagnostic, prognostic, and predictive tools, offering insights into patient stratification and the potential response to therapy. Furthermore, the reversible nature of epigenetic alterations presents an attractive target for therapeutic intervention, with several epigenetic drugs already in clinical use or under investigation for the treatment of CRC.^151^

In conclusion, epigenetic regulation is an integral component of colorectal cancer biology, influencing the disease course from its early stages through to advanced malignancy. A detailed elucidation of these regulatory mechanisms is critical for the development of novel diagnostic and therapeutic strategies aimed at improving patient outcomes.

### Importance of epigenetic modifications in CRC progression and therapy

The importance of epigenetic modifications in the progression of CRC cannot be overstated. These modifications contribute to the neoplastic process by influencing gene expression patterns that govern cellular proliferation, differentiation, apoptosis, and migration, ultimately leading to the malignant phenotype. Moreover, epigenetic changes are often early events in carcinogenesis, preceding genetic alterations, and thus represent a critical juncture at which intervention could potentially halt disease progression.^152^

DNA methylation changes are among the earliest detectable molecular alterations in CRC and are considered a driving force in the adenoma-carcinoma sequence.^153^ For example, the CIMP is characterized by widespread promoter hypermethylation and has been associated with specific clinical and pathological features of CRC. Importantly, CIMP status has been associated with differential responses to specific chemotherapeutic agents. Shen et al. conducted a study on 188 advanced CRC patients who underwent 5-fluorouracil (5-FU) based chemotherapy. Their findings revealed significant disparities in survival outcomes based on CIMP status: the median survival was 6 months for the CIMP-positive group compared to 17 months for the CIMP-negative group (P < 0.001). Furthermore, the two-year survival rate was markedly lower in the CIMP-positive group at 8%, versus 28% in the CIMP-negative group.^154^ These results underscore the importance of epigenetic modifications in guiding treatment stratifimrication in oncology.

Histone modification patterns in CRC also have prognostic and therapeutic implications. Global decreases in histone acetylation and specific alterations in histone methylation marks are associated with tumor progression and poor prognosis. As such, histone deacetylase inhibitors (HDACi) and histone methyltransferase inhibitors are being explored as potential therapeutic agents in CRC. These compounds have shown promise in preclinical models, and several are currently under clinical evaluation.^155^

The role of ncRNAs in CRC is an expanding area of interest. MiRNAs, in particular, have been implicated in CRC progression by modulating the expression of target genes involved in cell cycle regulation, apoptosis, and metastasis. Alterations in miRNA expression have been associated with chemotherapy resistance, suggesting that modulation of miRNA function could enhance therapeutic efficacy.^156^

Epigenetic therapy, aimed at reversing aberrant epigenetic modifications, has emerged as a novel strategy in CRC management. Agents such as DNA methyltransferase inhibitors (DNMTi) and HDACi are at the forefront of this approach. These drugs have the potential to reactivate silenced tumor suppressor genes and induce growth arrest, differentiation, or apoptosis in cancer cells.^157^ Furthermore, there is growing evidence that epigenetic drugs can modulate the immune response, thereby enhancing the efficacy of immunotherapeutic approaches such as checkpoint inhibitors.^158^

Combination therapies that include epigenetic drugs alongside conventional chemotherapies or targeted agents hold particular promise. By resetting the epigenetic landscape of cancer cells, these combinations may overcome drug resistance and lead to more durable responses. Additionally, epigenetic biomarkers may guide therapy selection and provide a means to monitor treatment response, allowing for personalized treatment regimens.^151^

In summary, the role of epigenetic modifications in CRC underscores their potential as biomarkers and therapeutic targets. Continued research into the epigenetic underpinnings of CRC progression and therapy is expected to yield novel insights that will enhance the precision and effectiveness of cancer treatment.

### Main types of epigenetic changes: DNA methylation, histone modifications, and ncRNAs

#### DNA methylation dynamics in CRC

##### Overview of DNA methylation and its role in gene expression

DNA methylation, specifically the addition of a methyl group to the fifth carbon of the cytosine ring within CpG dinucleotides, is a well-characterized epigenetic modification that plays a crucial role in the regulation of gene expression. In mammalian genomes, CpG dinucleotides tend to cluster into regions referred to as CpG islands, which are often located in gene promoter regions. The methylation status of these CpG islands is critical for the control of gene expression.^159^

In a general context, the methylation of CpG islands within promoter regions is associated with a repressive chromatin state, leading to the transcriptional silencing of the associated gene. This process is mediated by methyl-CpG-binding domain proteins (MBDs), which recognize methylated DNA and recruit additional chromatin remodeling factors that alter chromatin structure, thereby preventing the binding of transcription factors and the transcriptional machinery.^160^

During normal development and cell differentiation, DNA methylation patterns are established and maintained by the coordinated action of DNA methyltransferases (DNMTs), including DNMT1, DNMT3A, and DNMT3B. DNMT1 is primarily responsible for the maintenance of methylation patterns during DNA replication, whereas DNMT3A and DNMT3B are involved in de novo methylation processes.^161^

CRC exemplifies how aberrant DNA methylation patterns significantly contribute to disease pathogenesis. A common feature in CRC is global hypomethylation, which leads to genomic instability and the activation of oncogenic pathways. In contrast, hypermethylation frequently occurs at the promoters of tumor suppressor genes, leading to their silencing and the subsequent disruption of essential growth-regulatory and cell cycle checkpoint mechanisms.^162^ This complex landscape of DNA methylation alterations is a defining characteristic of CRC and gives rise to various methylation subtypes. One such subtype is the CIMP, which is distinguished by particular clinical and molecular features, including MSI and mutations in the BRAF gene.^20,163^ These epigenetic modifications in CRC underscore the intricate interplay between genetic and epigenetic mechanisms in tumorigenesis.

Recent advances in high-throughput sequencing technologies have facilitated the genome-wide analysis of DNA methylation patterns, known as methylome analysis, in CRC. Such studies have identified numerous differentially methylated regions (DMRs). In addition to the static view of methylation patterns, there is an increasing recognition of the dynamic nature of DNA methylation during CRC evolution. Environmental factors, dietary components, and the microbiome can influence the methylation status of CRC cells, thereby affecting gene expression and tumor behavior.

Given the reversible nature of DNA methylation, the use of DNMTi has emerged as a therapeutic strategy in CRC. These agents can demethylate silenced tumor suppressor genes, restore their expression, and induce cellular differentiation, apoptosis, or senescence in neoplastic cells. Moreover, DNA methylation markers are being explored as potential biomarkers for early detection, prognosis, and prediction of therapeutic responses in CRC.^164^

Overall, the study of DNA methylation dynamics in CRC provides a deeper understanding of the molecular mechanisms underlying tumorigenesis and offers promising avenues for the development of epigenetic therapies and diagnostic tools.

##### Mechanisms of DNA methylation alterations in CRC

In CRC, the DNA methylation landscape is characterized by a paradoxical co-existence of both hypermethylation and hypomethylation events. Hypomethylation predominantly occurs within repetitive DNA sequences, leading to CIN and the potential reactivation of transposable elements, which may contribute to oncogene activation. On the other hand, hypermethylation typically affects the promoter regions of tumor suppressor genes, resulting in their transcriptional silencing and the loss of critical growth regulatory functions.^165^

The CIMP is a distinct molecular subtype of CRC characterized by widespread hypermethylation of CpG islands. CIMP-positive tumors exhibit a high frequency of DNA hypermethylation at specific gene loci and are often associated with distinct clinical and pathological features. Weisenberger et al. investigate the CIMP in CRC and its association with BRAF mutations and MSI. By systematically analyzing 195 CpG island methylation markers in 295 primary human colorectal tumors, the study identifies CIMP-positive tumors as a distinct subset strongly linked to BRAF mutations, with an odds ratio of 203. The study also reveals that sporadic cases of mismatch repair deficiency in CRC are predominantly due to CIMP-associated methylation of the MLH1 gene. To classify CIMP-positive tumors accurately, Weisenberger et al. propose a robust new marker panel consisting of CACNA1G, IGF2, NEUROG1, RUNX3, and SOCS1. The findings emphasize the importance of CIMP in the underlying biology of CRC and its potential role in the development of personalized therapeutic strategies.^163^

DNA methyltransferases, including DNMT1, DNMT3A, and DNMT3B, are the enzymes responsible for the addition of methyl groups to cytosines. DNMT1 is primarily involved in the maintenance of methylation patterns following DNA replication, whereas DNMT3A and DNMT3B are responsible for establishing new methylation marks during cellular differentiation and development.^161^

In CRC, overexpression of DNMTs has been linked to the abnormal methylation patterns observed in the disease. DNMT1 has been shown to be upregulated and associated with the hypermethylation of tumor suppressor genes, contributing to the CIMP phenotype. Similarly, DNMT3B overexpression has been correlated with increased methylation levels and tumor progression.^161^

The role of DNMTs in CRC underscores the potential therapeutic benefit of targeting these enzymes. DNMT inhibitors, such as 5-azacytidine and decitabine, have shown promise in preclinical models by reversing aberrant methylation patterns and reactivating silenced genes. Clinical trials are ongoing to evaluate the efficacy of DNMT inhibitors as part of combination therapies for CRC.^166^

Overall, the dynamic alterations in DNA methylation observed in CRC highlight the complex regulatory mechanisms that govern epigenetic modifications and their impact on tumor biology. Further research into the mechanisms driving these methylation changes will be essential for developing targeted epigenetic therapies and improving the management of CRC.

Furthermore, it is worth noting that alterations in the expression of DNMTs can be both a cause and consequence of tumorigenesis. As such, they serve as potential biomarkers for CRC and targets for epigenetic therapy.^146^

##### Impact of DNA methylation on CRC signaling pathways

*Wnt/β-catenin Pathway:* The Wnt/β-catenin signaling cascade is quintessential for the maintenance of intestinal homeostasis and is frequently dysregulated in CRC due to epigenetic alterations. DNA methylation of Wnt antagonists such as the SFRP family, WIF1, and DKK3 has been documented, leading to their transcriptional silencing and consequent disinhibition of the Wnt/β-catenin pathway. This epigenetic silencing facilitates the stabilization and nuclear translocation of β-catenin, culminating in the transcriptional activation of target genes that promote cell proliferation and survival. Therapeutic strategies aimed at reversing these methylation patterns could restore the expression of these critical Wnt pathway regulators and suppress tumorigenesis.^167^ Inhibition of DNMTs markedly diminishes the stem-like properties of colorectal cancer cells. This effect is mediated through the demethylation of genes that inhibit the Wnt pathway, leading to a downregulation of the Wnt/β-catenin signaling cascade. Furthermore, the methylation of the SFRP1 gene is crucial for sustaining the stem cell population within colorectal tumors.^168^

*TGF-β signaling:* TGF-β signaling exerts complex effects on CRC cells, acting as a tumor suppressor in early lesions but promoting invasion and metastasis in advanced stages. DNA methylation-induced inactivation of TGF-β receptors (such as TGFBR2) and downstream effectors (including SMAD4) can disrupt this pathway, facilitating escape from growth inhibitory signals. Furthermore, hypermethylation of TGF-β pathway inhibitors may exacerbate signaling, contributing to the epithelial-mesenchymal transition and metastatic potential of CRC cells.^169^ Modulation of DNA methylation patterns in these key nodes of TGF-β signaling may offer a therapeutic lever to mitigate CRC progression.

*PI3K/AKT/mTOR pathway:* The PI3K/AKT/mTOR signaling axis is integral to cell growth, metabolism, and survival, and its dysregulation through aberrant DNA methylation patterns is a hallmark of various cancers, including CRC. The tumor suppressor gene PTEN, which antagonizes PI3K/AKT signaling, often undergoes hypermethylation that correlates with reduced expression and enhanced pathway activity. This epigenetic alteration not only accelerates tumor growth but also confers resistance to certain therapeutic agents, underscoring the need for epigenetic therapies that can restore PTEN function and attenuate the oncogenic signaling.^170^

##### DNA methylation as a biomarker for CRC diagnosis and prognosis

DNA methylation patterns are increasingly recognized as valuable biomarkers for the diagnosis and prognosis of CRC. In CRC, global hypomethylation and gene-specific hypermethylation events are prevalent and can serve as molecular signatures reflecting neoplastic transformation and tumor progression.^171^

The phenomenon of CIMP, characterized by widespread hypermethylation of CpG islands, is associated with distinct clinical and pathological features of CRC, including MSI and BRAF mutations.^172^ Clinical studies confirmed that none of the tumors of MMR germline mutations cases showed positive for BRAFV^600E, suggesting that BRAF^V600E mutation-specific immunostaining has a low risk of excluding Lynch syndrome patients. MMR status has been proposed as a prognostic marker, with evidence suggesting that CIMP-high tumors may have a different clinical outcome compared to CIMP-low or CIMP-negative tumors (so-called Ogino and Weisenberger panels).

Additionally, hypermethylation of specific genes such as SEPT9, MLH1, and CDKN2A (p16) has been explored as diagnostic and prognostic indicators. The SEPT9 DNA methylation assay, for example, is a non-invasive blood test that detects methylated SEPT9 DNA as a biomarker for CRC screening, demonstrating reasonable sensitivity and specificity in detecting both early-stage and advanced CRC.^173^

The loss of MLH1 function due to promoter hypermethylation leads to MSI, which is a hallmark of a subset of CRCs. The presence of MSI in CRC, secondary to MLH1 methylation, is associated with a better prognosis and has implications for treatment decisions, including the potential benefit from immunotherapy.^174^

The methylation status of CDKN2A is associated with tumor progression and has been correlated with decreased survival in CRC patients, indicating its potential as a prognostic biomarker. The epigenetic silencing of CDKN2A results in the loss of its product p16, an important regulator of the cell cycle, thus contributing to uncontrolled cell proliferation.^175^

Methylome analysis using high-throughput techniques such as next-generation sequencing (NGS) and array-based platforms has facilitated the identification of novel methylation biomarkers with diagnostic and prognostic value. These approaches enable comprehensive profiling of the CRC epigenome and may lead to the discovery of methylation signatures that could refine risk stratification and guide personalized treatment strategies.^176^

In summary, DNA methylation-based biomarkers hold promise for improving the detection and management of CRC. Nevertheless, further validation in large-scale, prospective studies is required before these biomarkers can be fully integrated into clinical practice.

#### Histone modification patterns in CRC

##### Overview of core histone modifications: acetylation, methylation, phosphorylation

Histone proteins, around which DNA is tightly coiled, undergo various post-translational modifications (PTMs) that serve as a complex regulatory code, often referred to as the “histone code,” which influences chromatin structure and gene expression.^177^ Acetylation of histone lysine residues, catalyzed by histone acetyltransferases (HATs), neutralizes the positive charge on the histones, leading to a relaxed chromatin state and facilitating transcriptional activation. Methylation of histone lysine and arginine residues, mediated by histone methyltransferases (HMTs), can have varying effects on transcription, with certain methylation marks associated with active gene expression and others with gene repression.^178^ Phosphorylation, involving the addition of phosphate groups to serine, threonine, or tyrosine residues by histone kinases, is implicated in the regulation of chromatin condensation, DNA repair, and transcriptional activation in response to signaling events.^179^

##### Enzymes involved in histone modifications and CRC

*Histone Acetyltransferases (HATs) and Deacetylases (HDACs):* Within the CRC landscape, dysregulation of HATs, such as p300 and CBP, has been connected to oncogenic processes through the upregulation of gene expression that promotes tumor progression. HDACs, conversely, are generally associated with gene repression and can contribute to tumorigenesis when aberrantly expressed or mutated. HDACs, such as HDAC2, have been found to be overexpressed in CRC, correlating with poor prognosis, and HDAC inhibitors (HDACis), such as vorinostat and trichostatin A (TSA), have shown therapeutic potential in preclinical CRC models.^180^

*Histone Methyltransferases (HMTs) and Demethylases (HDMs):* Aberrations in HMTs such as EZH2, which is responsible for the methylation of H3K27, have been associated with the silencing of tumor suppressor genes and poor clinical outcomes in CRC. On the other hand, mutations or altered expression of HMTs like SETD2, which methylates H3K36, can lead to genomic instability and contribute to CRC pathogenesis. HDMs, such as the Jumonji domain-containing protein (JMJD) family and LSD1, which demethylate specific histone lysine residues, have been shown to modulate transcription in CRC and represent potential therapeutic targets. For example, inhibition of LSD1 has demonstrated efficacy in reducing proliferation and inducing apoptosis in CRC cell lines.^181^

Therefore, the enzymes responsible for writing, reading, and erasing histone modifications are crucial not only for normal chromatin function but also in the etiology of CRC. These enzymes represent potential biomarkers for disease progression and therapeutic targets, as evidenced by ongoing clinical trials evaluating the efficacy of epigenetic modulators in CRC treatment.^182^

##### Consequences of histone modifications on CRC signaling pathways

*Regulation of Apoptosis and Cell Cycle Genes:* Histone modifications have significant implications for the transcriptional regulation of apoptosis and cell cycle-related genes in CRC, which can impact cancer cell survival and proliferation.

*Acetylation and deacetylation:* HATs such as p300 and CBP play a pivotal role in the regulation of genes involved in apoptotic pathways by adding acetyl groups to histones, thus facilitating an open chromatin conformation conducive to gene transcription.^183^ The acetylation of histone H3 on lysine 27 (H3K27ac), for example, has been associated with the activation of pro-apoptotic genes. Conversely, HDACs, such as HDAC2, have been found to be overexpressed in CRC and correlate with the repression of tumor suppressor genes, including those involved in apoptosis.^184^

*Methylation and demethylation:* Histone methylation is another critical layer of regulation in CRC. Enhancer of zeste homolog 2 (EZH2), the catalytic subunit of the PRC2 complex, mediates the trimethylation of H3K27 (H3K27me3), which is commonly associated with gene repression. EZH2 overexpression in CRC has been linked to the downregulation of tumor suppressor genes, thus inhibiting apoptosis and promoting cell cycle progression. Inhibitors of EZH2, such as tazemetostat, have shown promise in reactivating these genes and inducing apoptosis in CRC cells.^185^

*Crosstalk with DNA methylation and chromatin remodeling:* Epigenetic modifications in CRC are not isolated to histone changes but also encompass DNA methylation and chromatin remodeling, with significant crosstalk between these processes.

In the context of CRC, DNA hypermethylation often occurs at promoter CpG islands of tumor suppressor genes, leading to their transcriptional silencing. This methylation can attract proteins like MBDs, which further recruit HDACs and HMTs, compounding the repressive chromatin state.^146^ For example, the gene encoding the DNA mismatch repair protein MLH1 is frequently silenced by DNA methylation in CRC, contributing to MSI and tumorigenesis.^186^

*Chromatin remodeling complexes:* Chromatin remodelers, such as the SWI/SNF complex, are crucial for the regulation of gene expression by altering nucleosome positioning. Mutations in SWI/SNF complex members, like ARID1A, result in dysregulated gene expression and have been implicated in CRC development. The interplay between chromatin remodelers and histone modifiers, such as the recruitment of PRC2 by SWI/SNF subunits, underscores the complex regulation of gene expression in CRC.^187^

In conclusion, the dynamic modifications of histones, in conjunction with DNA methylation and chromatin remodeling, play a fundamental role in the epigenetic regulation of genes critical for CRC pathogenesis. Targeting these modifications offers therapeutic potential in restoring the expression of tumor suppressor genes and promoting apoptotic pathways in CRC.

##### Therapeutic targeting of histone modifications in CRC

The therapeutic targeting of histone modifications in CRC represents an emerging and promising approach in oncology. The pharmacological manipulation of enzymes responsible for histone modifications can potentially reverse the aberrant epigenetic landscape characteristic of CRC and restore normal gene function.

*HDAC Inhibitors:* HDACi are among the most studied epigenetic drugs for CRC treatment. HDACi, such as vorinostat and romidepsin, have shown efficacy in preclinical models by inducing apoptosis, cell cycle arrest, and differentiation in CRC cells. Clinical trials have explored these agents, either as monotherapies or in combination with traditional chemotherapies or targeted agents, to enhance antitumor efficacy and overcome resistance.^188^

*EZH2 Inhibitors:* The inhibition of EZH2, the catalytic subunit of the polycomb repressive complex 2 (PRC2), has also been investigated as a therapeutic strategy in CRC. EZH2 is often overexpressed in CRC and associated with poor prognosis. Small molecule inhibitors of EZH2, such as EPZ-6438 (tazemetostat), can decrease the trimethylation of H3K27, leading to re-expression of silenced genes involved in growth inhibition and apoptosis.^189^ Early-phase clinical trials are evaluating the safety and efficacy of EZH2 inhibitors in solid tumors, including CRC.^190^

*Bromodomain and Extra-terminal domain (BET) Inhibitors:* BET inhibitors are a class of drugs that target bromodomain proteins, which recognize acetylated lysine residues on histone tails. By inhibiting the BET family of proteins, such as BRD4, these inhibitors can modulate the expression of oncogenes and genes involved in cell cycle progression and apoptosis. In CRC, BET inhibitors have shown preclinical activity and are currently being tested in clinical trials.^191^

*Combination Therapies:* Given the complexity of histone modification patterns and their interplay with other epigenetic mechanisms, combination therapies that target multiple epigenetic regulators simultaneously may be particularly effective. For instance, combining HDACis with DNMTi has been shown to have synergistic effects in CRC models, leading to the reactivation of silenced tumor suppressor genes and enhanced antitumor activity.^192^

#### Non-coding RNAs and their regulatory roles in CRC

The intricate world of ncRNAs has revealed a myriad of new regulatory mechanisms that contribute to the pathogenesis of CRC. These ncRNAs, which do not code for proteins, exert their influence by modulating gene expression at various levels, including chromatin remodeling, transcription, and post-transcriptional processing.

##### Classification of non-coding RNAs: miRNAs, lncRNAs, and circRNAs

Non-coding RNAs are classified into various categories based on their size and structural characteristics. MiRNAs are short ncRNAs approximately 22 nucleotides in length that regulate gene expression post-transcriptionally. LncRNAs are transcripts longer than 200 nucleotides that function through diverse mechanisms, including chromatin modification, transcriptional interference, and serving as molecular scaffolds.^150^ CircRNAs are a novel class of ncRNA with a closed loop structure that can act as miRNA sponges, affecting the stability and translation of mRNAs.

##### miRNAs and their influence on CRC signaling pathways

In CRC, particular attention has been given to miRNAs that target key tumor suppressor genes and oncogenes involved in cell signaling pathways. For instance, miR-135 and miR-155 have been shown to target APC, a negative regulator of the Wnt signaling pathway, which is frequently mutated in CRC.^193^ Additionally, miRNAs such as let-7 and miR-143 target the oncogene KRAS, influencing the MAPK/ERK signaling cascade and thereby impacting cell proliferation and survival.^194^ The tumor suppressor gene TP53 is also regulated by several miRNAs, including miR-125b, which modulates the p53-dependent apoptotic pathway.^195^

##### miRNAs as tumor suppressors or oncogenes

miRNAs can act as tumor suppressors or oncogenes (oncomiRs) depending on the context and the target genes they regulate. miR-34a, for example, is a well-characterized tumor suppressor miRNA that is transcriptionally activated by p53 and can induce cell cycle arrest and apoptosis by targeting multiple oncogenes. Conversely, miR-21 is an oncomiR that is frequently upregulated in CRC and promotes tumor growth and metastasis by targeting tumor suppressor genes such as PTEN and PDCD4.^196^

In summary, ncRNAs, particularly miRNAs, play pivotal roles in the regulation of CRC pathogenesis through their impact on critical signaling pathways. These ncRNAs offer potential as diagnostic biomarkers and therapeutic targets; however, the challenge lies in the delivery of ncRNA-based therapeutics and in understanding the complexity of their regulatory networks.

##### Potential of non-coding RNAs as therapeutic targets and biomarkers

The functional versatility of ncRNAs in gene regulatory networks renders them attractive targets for therapeutic intervention. One of the most promising strategies involves the use of oligonucleotide-based therapies, such as small interfering RNAs (siRNAs) or antisense oligonucleotides (ASOs), to modulate the activity of ncRNAs. For instance, the miR-34a mimic MRX34 was one of the first miRNA-based therapies to enter clinical trials, though it was discontinued due to immune-related adverse effects. Despite this setback, the field is progressing with other candidates, such as the lncRNA H19-targeting agent BC-819, which has demonstrated potential in early-phase clinical studies.^197^

In addition to synthetic oligonucleotides, the use of small molecules to modulate ncRNA function is an emerging area of research. These molecules can alter the biogenesis, stability, or interaction of ncRNAs with their binding partners. For instance, ASOs that disrupt the interaction between the oncogenic lncRNA MALAT1 and its associated proteins are under investigation.^198^

##### Potential of non-coding RNAs as biomarkers

ncRNAs possess several features that make them excellent candidates as biomarkers for cancer detection, prognosis, and monitoring therapeutic response. Their stability in body fluids, disease-specific expression patterns, and relative ease of detection via non-invasive or minimally invasive samples are compelling attributes for clinical application.^199^

miRNAs, in particular, have been extensively studied as biomarkers in various cancers, including CRC. For example, a panel of miRNAs, such as miR-21 and miR-92a, has been reported to be elevated in the plasma of CRC patients and could serve as potential diagnostic markers.^200^ lncRNAs, such as PCA3 in prostate cancer, have been approved for use in diagnostic assays, indicating the clinical viability of ncRNA-based diagnostics.^201^

circRNAs are also gaining attention as potential biomarkers due to their stability and specificity. circRNA_002178 can be detected at higher levels in the tissues of liver cancer patients and may serve as a novel biomarker for hepatocellular carcinoma.^202^

In conclusion, ncRNAs offer immense potential as therapeutic targets and biomarkers in the context of cancer. The continued elucidation of their biological functions and mechanisms of action will undoubtedly lead to the refinement of ncRNA-based interventions and their translation into clinical utility.

## Gut microbiota’s role in CRC signaling pathways

### The influence of gut microbiota on colorectal cancer development

The association between gut microbiota and the pathogenesis of CRC has been an area of active research for decades. Initial insights into this relationship were gleaned from animal models in the latter half of the 20th century. Studies utilizing germ-free versus conventionally raised rodents provided compelling evidence that gut microflora plays a pivotal role in the development of CRC. A seminal study from the late 1960s discovered that the presence of intestinal microbiota was necessary for the carcinogenic activity of cycasin, as evidenced by the absence of cancer development in germ-free rodents compared to their conventional counterparts.^203^

Further experiments employing the carcinogenic compound 1,2-dimethylhydrazine demonstrated a stark contrast in tumor incidence between germ-free and conventional rats, with the former exhibiting significantly fewer colonic tumors.^204^ The involvement of specific bacterial genera, such as Escherichia, Enterococcus, Bacteroides, and Clostridium, was later identified as a contributing factor in colorectal carcinogenesis by promoting an increase in aberrant crypt foci formation.^205^

In line with these findings, fecal transplants from CRC patients into mice not only heightened intestinal epithelial cell proliferation in germ-free mice but also escalated tumor development in mice exposed to azoxymethane, a chemical inducer of colon neoplasia.^206^

In human research, comparative metagenomic and metataxonomic analyses have been employed to elucidate the composition of the gut microbiota in CRC patients versus healthy individuals. These studies have consistently revealed distinct differences in the microbial communities between these populations. Increased microbial diversity and a shift in the relative abundance of specific taxa have been observed, with a decrease in taxa such as Roseburia that may confer protective effects and an enrichment of taxa with pro-carcinogenic potential, including Bacteroides, Escherichia, Fusobacterium, and Porphyromonas.^207^

These investigations underscore the functional significance of the microbial milieu in CRC and suggest a potential consortium of microorganisms that may contribute to oncogenesis.

### Characterizing the colorectal cancer microbiota

The advent of high-throughput human shotgun metagenomics and 16S rRNA gene sequencing has been pivotal, paving the way for a more comprehensive characterization of the CRC microbiota. These technologies have enabled us to study microbial communities from both fecal and mucosal specimens, providing insights into the gut’s luminal versus mucosa-associated microbiota, respectively.^208^

**Fig. 5:**
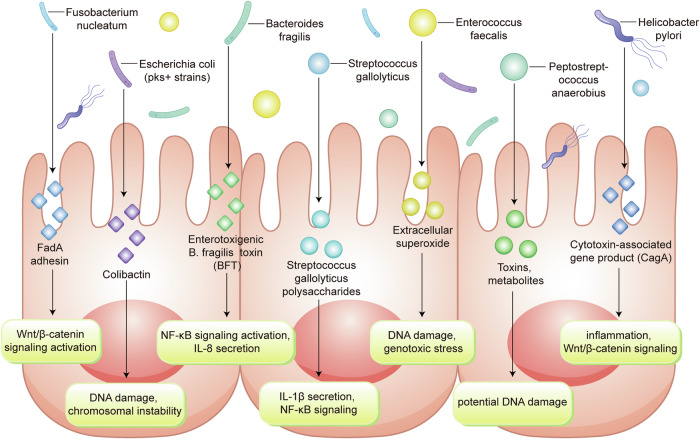
Interplay between colorectal cancer pathogenesis and the gut microbiota. This figure illustrates the interaction between various gut microbiota and the development of colorectal cancer. Specific bacteria, such as Fusobacterium nucleatum, Escherichia coli (pks+ strains), Bacteroides fragilis, Streptococcus gallolyticus, Enterococcus faecalis, Peptostreptococcus anaerobius, and Helicobacter pylori, produce factors that contribute to cancer pathogenesis. These factors include FadA adhesin, colibactin, B. fragilis toxin (BFT), extracellular superoxide, and cytotoxin-associated gene product (CagA), which lead to processes like Wnt/β-catenin signaling activation, NF-κB signaling activation, IL-8 secretion, IL-1β secretion, DNA damage, chromosomal instability, genotoxic stress, and inflammation. The Figure underscores the significant role of gut microbiota in influencing colorectal cancer pathways

The evidence amassed from these studies consistently points to a stark contrast between the microbial consortia in CRC patients and those in healthy individuals. This shift, termed dysbiosis, is not merely a fluctuation in microbial diversity but signifies a profound alteration in the ecological landscape that may contribute to the pathogenesis of CRC.^207^ The specific bacterial strains that have been recurrently associated with CRC, such as Bacteroides fragilis, Escherichia coli, Enterococcus faecalis, and Streptococcus gallolyticus, have been the subject of numerous association and mechanistic studies.^209,210^ Their individual roles in promoting carcinogenesis are gradually being elucidated, with links to inflammation, genotoxicity, and immune modulation^94,97^ (Fig. 5).

Moreover, metagenomic studies have brought to light associations with other bacteria that were previously underappreciated in the context of CRC. For example, Fusobacterium nucleatum, a bacterium notorious for its pro-inflammatory and adhesion properties, has been found to be more abundant in both fecal and tumor samples from CRC patients.^211^ Similarly, members of the Parvimonas, Peptostreptococcus, Porphyromonas, and Prevotella genera have been identified as being more prevalent in the CRC microbiota.^212^

The potential diagnostic value of these microbial shifts is immense. The relative abundance of specific bacterial taxa can be quantified, and such fold-changes may serve as robust biomarkers for CRC detection.^213^ Meta-analyses have underscored the consistency of certain bacterial associations with CRC, irrespective of geographical disparities in gut microbiota composition. This suggests that, despite the inherent variability in microbiota across populations, there may be a universal microbial signature associated with CRC.

In previous meta-analysis, incorporating a substantial collection of fecal shotgun metagenomic datasets, authors distilled a core set of seven bacteria that were notably enriched in CRC.^208^ Among them were the enterotoxigenic B. fragilis and a subset of oral bacteria including F. nucleatum, Parvimonas micra, Porphyromonas asaccharolytica, and Prevotella intermedia. The inclusion of Alistipes finegoldii and Thermanaerovibrio acidaminovorans completes this core microbiota, which interestingly shows a negative correlation with a consortium of bacteria that are depleted in CRC.^208^

The implications of this microbial dysbiosis extend beyond mere correlation. For instance, the antagonistic relationship between the enriched and depleted taxa suggests a disruption of the mutualistic networks that maintain gut homeostasis. Some of the depleted species, such as Clostridium butyicum, a known butyrate producer, and Streptococcus thermophilus, have established roles in maintaining gut health and have been utilized in probiotic formulations aimed at preventing antibiotic-associated diarrhea in infants.^214^ These probiotic candidates, through their antagonistic action on pathobionts, present a tantalizing prospect for CRC therapy or prevention.

Subsequent research efforts have continued to refine the microbial signature of CRC, identifying up to 29 core species consistently enriched across multiple geographic regions, underscoring the potential of these microbial markers in CRC screening strategies.^207^

Furthermore, the gut microbiota’s complexity is augmented by the presence of viruses and fungi, each playing a role that is yet to be fully understood. Molecular and histological techniques have identified various viruses within CRC tissues, including cytomegalovirus, John Cunningham virus, and human papillomavirus, although the associations have been mixed and at times contradictory. Nonetheless, in an expansive untargeted metagenomic analysis, we observed a distinct alteration in the enteric virome of CRC patients, identifying 22 viral taxa, including cytomegalovirus, capable of distinguishing CRC cases from controls with a significant degree of accuracy.^215^

Bacteriophage dynamics within the CRC microbiota have also been scrutinized, revealing that cancer-associated viromes are predominantly composed of temperate bacteriophages, which may act as key nodes within bacterium-virus community networks.^216^ This observation has led to the hypothesis that transkingdom interactions, especially between bacteria and viruses, might be integral to the tumorigenic process in CRC.

The fungal component of the gut microbiota—termed the mycobiome—has received less attention but is increasingly recognized as playing an important role in CRC. A shift towards a higher abundance of the genus Malassezia, among other fungi, in the CRC mycobiome suggests potential transkingdom interactions that could influence disease progression.^217^ These fungal elements, though less studied, are gaining recognition for their possible involvement in the modulation of the immune response and the integrity of the gut barrier—factors that are critical in the development and progression of CRC.

The exploration of such transkingdom interactions is not without its challenges. The complex interplay between various microbial kingdoms necessitates advanced bioinformatic tools capable of constructing and analyzing multi-omic networks. The integration of metagenomic, metatranscriptomic, and metaproteomic data is essential to unravel the functional dynamics of these microbial communities within the CRC environment.^218^ Indeed, spatial multi-omics, an approach that combines these multi-layered omics data with spatial resolution, offers an unprecedented opportunity to dissect the heterogeneity and localization of microbial populations in relation to tumor architecture.^219^

In summary, the diversification of the microbiota in the context of CRC is a dynamic and multifaceted process. The advancement of high-throughput omics technologies and their integration into a spatial framework will undoubtedly enhance our understanding of the microbial underpinnings of CRC. Such insights could pave the way for the development of novel diagnostics, therapeutics, and preventive strategies that exploit the intricate microbial landscape of the gut.

### Immune dynamics and inflammatory contributions to colorectal carcinogenesis

The gastrointestinal interface is a pivotal arena where the gut microbiota and the host immune system engage in critical crosstalk, maintaining homeostasis and influencing disease outcomes. Chronic inflammation is not only a defining feature but also a significant risk factor for CRC,^220^ as exemplified by the increased incidence of CRC in individuals with IBD. Literature has documented a 30-year cumulative CRC risk of 18.4% for ulcerative colitis and 8.3% for Crohn’s disease, with variations attributable to the heterogeneity of study populations, healthcare settings, and clinical practices.^221^

Microbial constituents of the gut have been identified as key modulators of inflammatory processes within the gastrointestinal tract (Fig. 5). Fecal microbiota transplantation studies from CRC patients into germ-free or carcinogen-exposed rodents have shown an augmentation of histological inflammation and upregulated expression of inflammatory genes, suggesting that gut microbes can direct the mobilization of immune cells, such as cytotoxic T lymphocytes and TH1 cells, to tumor sites by the production of chemotactic factors.^222^

Specific microbial species like Fusobacterium nucleatum have been implicated in the activation of the nuclear factor-κB pathway, leading to the infiltration of myeloid cells into tumors and promoting a pro-inflammatory state that is conducive to neoplastic transformation in genetically predisposed rodents.^211^ Similarly, Enterotoxigenic Bacteroides fragilis, which is overrepresented in the CRC patient microbiota, can elicit inflammatory responses in colonic epithelial cells via its toxin, resulting in a recruitment of immature myeloid cells to the distal colon, thereby precipitating an inflammatory milieu.^209^

Additionally, other microbes such as pks+ Escherichia coli, Enterococcus faecalis, and Alistipes finegoldii are associated with inflammation-driven oncogenic activities within the colorectal environment.^210^

Pattern recognition receptors (PRRs) are crucial in mediating the interaction between microbial antigens and the immune system, triggering host immune responses. These receptors, which encompass Toll-like receptors (TLRs), nucleotide-binding oligomerization domain-like receptors, RIG-I-like receptors, and absent in melanoma 2-like receptors, play a role in the pathogenesis of colitis-associated cancer in animal models.^223^ Notably, F. nucleatum can activate TLR4 signaling, thereby enhancing tumor development, while Peptostreptococcus anaerobius has been shown to promote CRC through TLR2 and TLR4 pathways in rodent models.^224^

### Dietary metabolite interactions with the gut microbiome

The interplay between the gut microbiota and the host’s metabolism is a critical point of intersection (Fig. 5). The expansive genetic capacity of the microbiota allows for the breakdown of a variety of dietary substances, including complex carbohydrates that are resistant to human digestive enzymes, such as galacto-oligosaccharides and fructo-oligosaccharides, as well as the transformation of host-derived molecules like bile acids. Building upon the seminal work by Doll and Peto, which attributed about 35% of cancer cases to diet, more recent epidemiological data suggest that nearly 38.3% of new CRC instances might be associated with suboptimal dietary patterns characterized by low intake of whole grains and dairy, alongside high consumption of processed meats. Furthermore, certain dietary elements have been implicated in CRC risk, particularly red and processed meats.^225^ Compounds emerging from microbial metabolism, such as N-nitroso compounds and hydrogen sulfide, have been identified as procarcinogenic. In murine studies, the gut microbiota has been shown to exacerbate heme-induced epithelial proliferation and compromise the integrity of the mucus barrier.^226^

Short-chain fatty acids (SCFAs), predominantly butyrate and propionate, are central to the conversation on microbial metabolites and CRC. These SCFAs are the result of the fermentation of indigestible carbohydrates like dietary fibers and resistant starches in the colon. Butyrate is celebrated for its anti-inflammatory properties and ability to inhibit HDACs in colonocytes and immune cells, which leads to a downregulation of pro-inflammatory cytokines and induction of apoptosis in CRC cells.^227^ Both butyrate and propionate have displayed immunomodulatory effects, particularly influencing colonic regulatory T cells (Tregs) in animal models, thereby exerting anti-inflammatory actions. SCFA levels are notably decreased in populations with heightened CRC risk, including African Americans and individuals with ulcerative colitis or advanced colorectal adenomas.^228^ While butyrate is often considered anti-tumorigenic, it has also been reported to drive aberrant cell proliferation in certain in vitro contexts. This contradiction may arise from the complex interplay between host genetics, metabolic environment, and the presence of other metabolites.

Another crucial set of metabolites are bile acids, which are synthesized by the liver and converted into secondary bile acids by intestinal bacteria.^229^ High-fat diets have been associated with increased levels of secondary bile acids like deoxycholic acid in the colon, which correlates with an elevated CRC risk. Experimental models have further underscored the potential of bile acids in promoting tumorigenesis; for example, rats subjected to surgical bile diversion and subsequent exposure to carcinogens developed increased colonic tumors. Deoxycholic acid, in particular, is known to induce oxidative DNA damage in vitro and foster tumor growth in vivo.^230^

### Synthesis of genotoxic agents

The microbiome has been implicated in carcinogenesis through various mechanisms, one of which includes the synthesis of agents that can damage DNA. Known as genotoxins, these substances are secreted by certain bacteria and can lead to direct genomic instability (Fig. 5). Examples include the cytolethal distending toxin (CDT) and colibactin. CDT is secreted by specific enteric pathogens such as some species of Escherichia and Campylobacter, which is known to cause double-stranded breaks in DNA due to its inherent DNase activity.^231^ Research using animal models of CRC has shown that strains lacking CDT exhibit a reduced capacity to induce tumors.^232^ Colibactin, synthesized by certain Enterobacteriaceae, also induces DNA strand breaks. Furthermore, Bacteroides fragilis toxin (BFT)^209^ and the production of reactive oxygen species by Enterococcus faecalis have been linked with DNA damage and CIN in experimental settings. The potential therapeutic or preventive benefits of blocking these toxins are significant; for instance, inhibitors that impede colibactin biosynthesis have demonstrated tumor reduction in murine models.^233^

In relation to translational medicine, while a comprehensive analysis of CRC mechanisms is beyond our present discussion, it is crucial to highlight those microbiota-related factors that have tangible clinical implications. Delving into the metagenomics of CRC-associated microbiota may offer promising biomarkers, and understanding the microbiota’s mechanisms may present novel targets for cancer prophylaxis and treatment. It is also vital to differentiate between the roles of bacteria in the driver-passenger model of carcinogenesis:^234^ the driver bacteria, which can directly cause cancer, and the passenger bacteria, which thrive in the TME and could potentially be exploited for diagnosis, prevention, or treatment strategies. Recognizing the specific ecological function of each bacterium is essential in developing strategic approaches to translate these microbial insights into practical clinical applications.

### Leveraging the intestinal microbiome for colorectal cancer therapy

**Fig. 6:**
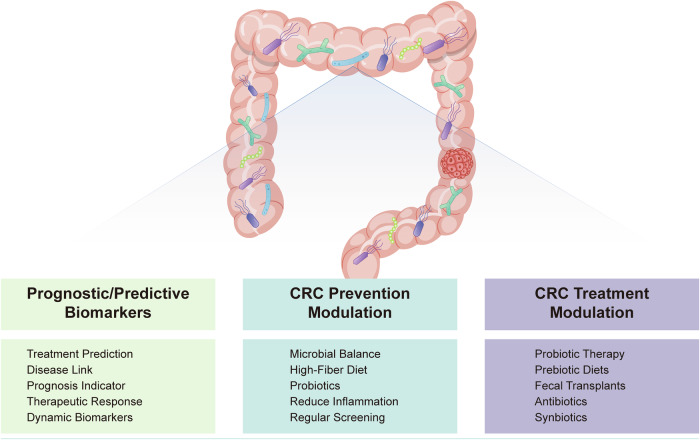
Therapeutic modulation of the gut microbiota in colorectal cancer management. This graph outlines prognostic/predictive biomarkers, CRC prevention modulation, and CRC treatment modulation

The intestinal microbiome’s influence on CRC treatments is increasingly recognized as a pivotal area of investigation. The microbiome is implicated not only in the oncogenic process and tumor development but also in modulating responses to both chemotherapy and immunotherapy, offering promise as both a predictive biomarker and a target for therapeutic modulation to enhance patient outcomes^235^ (Fig. 6).

Chemotherapeutics, such as 5-FU, cyclophosphamide, gemcitabine, and oxaliplatin, exhibit altered efficacy in relation to the gut microbiota through a variety of mechanisms, including microbial translocation and immunomodulation. Studies in rodent models reveal that the absence of a complex gut microbiome can impair myeloid-derived cell function, undermining chemotherapeutic efficacy.^236^ Furthermore, Fusobacterium nucleatum’s interaction with autophagy mechanisms can promote chemoresistance to agents like oxaliplatin,^237^ suggesting potential targets for enhancing treatment outcomes. The metabolism of irinotecan, a topoisomerase inhibitor, is profoundly influenced by the gut microbiota. This drug is metabolized to SN-38 and subsequently inactivated by hepatic glucuronidation. However, bacterial β-glucuronidase enzymes in the gut can reverse this process, leading to the reactivation of SN-38 and subsequent gastrointestinal toxicity. Strategies to inhibit these bacterial enzymes have demonstrated potential in ameliorating such adverse effects in murine models.^238^

In the realm of immunotherapy, the gut microbiota is essential for an optimal response, influencing the efficacy of checkpoint inhibitors targeting PD-1/PD-L1 and CTLA-4 pathways.^239,240^ Certain bacterial species, such as Akkermansia muciniphila and Bifidobacterium spp., have been associated with improved responses to these therapies.^239^ Moreover, oral administration of specific bacterial strains can restore anti-tumor immunity in antibiotic-disrupted mouse models.^240^ Meta-analyses across multiple studies have identified key bacterial taxa correlated with positive responses to immunotherapy, and consortia of bacteria have been shown to potentiate checkpoint inhibitor efficacy in preclinical models.^241^ These discoveries underscore the potential for microbial profiling to guide immunotherapeutic interventions. Adverse events linked to immunotherapy, such as checkpoint inhibitor-induced colitis, also appear to be influenced by microbiome composition, with certain bacterial phyla associated with either increased resistance or susceptibility to such effects.^242^ Fecal microbiota transplantation has been reported as a successful intervention in cases of refractory colitis following immunotherapy. While immunotherapy may not uniformly succeed across all CRC subtypes, it has shown particular promise in microsatellite instability-high (MSI-H) or DNA mismatch repair-deficient metastatic CRCs, characterized by a high mutational burden and upregulated immune checkpoints.^243^ The microbiota offers a promising avenue to potentiate immunotherapy’s effectiveness and reduce associated adverse effects.

Other therapeutic strategies targeting the microbiota are in development, leveraging microbial agents or their derivatives for cancer treatment. These include the use of antibiotics to target cancer-associated bacteria, probiotics to enhance antitumor immune responses, and small molecule inhibitors to mitigate chemotherapy-induced toxicity.^239,240^ Clinical trials are exploring the role of fecal microbiota transplantation in conjunction with cancer therapies.^244^ Furthermore, the manipulation of microbial metabolites through dietary interventions and bioengineering approaches are promising strategies under examination. In conclusion, the gut microbiome presents a frontier for innovative CRC treatment strategies, with the potential to personalize therapy and improve patient outcomes. A deeper understanding of host-microbiome interactions will be instrumental in translating these findings into effective clinical applications.

## Targeted therapy: current strategies and pathway interactions

### Rationale for targeted therapy: precision medicine approach

The inception of targeted therapy in oncology stems from the paradigm shift toward precision medicine, which emphasizes the tailoring of medical treatment to the individual characteristics of each patient. Unlike conventional chemotherapeutic agents that indiscriminately affect both cancerous and normal dividing cells, targeted therapies are designed to interfere with specific molecular targets that are involved in the growth, progression, and spread of cancer.^245^

The rationale for this approach is grounded in the understanding that cancer is not a single disease but a collection of diverse disorders with varied genetic and epigenetic alterations. The advent of high-throughput genomic technologies has facilitated the identification of actionable mutations and aberrant signaling pathways that are critical for tumor cell survival and proliferation.^246^ By focusing on these molecular aberrations, targeted therapies can block the growth and spread of tumors while minimizing damage to normal cells, thus offering potentially higher efficacy and lower toxicity.

### Overview of hallmark pathways in cancer and rationale for targeting them

Cancer cells exhibit several hallmark capabilities that enable tumor growth and metastatic dissemination. These include sustaining proliferative signaling, evading growth suppressors, resisting cell death, enabling replicative immortality, inducing angiogenesis, and activating invasion and metastasis.^3^ The dysregulation of various signaling pathways often underlies these hallmark traits, thereby presenting logical targets for therapeutic intervention.

One of the most critical pathways implicated in cancer is the Wnt/β-catenin signaling pathway, which plays a pivotal role in cell proliferation and differentiation. Aberrant activation of Wnt signaling is a common feature in several cancers, such as colorectal cancer, and is associated with poor prognosis.^247^ Targeting this pathway could potentially impair cancer cell proliferation and induce apoptosis.

Similarly, the EGFR pathway is frequently dysregulated in cancers like non-small cell lung cancer (NSCLC) and colorectal cancer.^248^ EGFR mutations or overexpression lead to uncontrolled cell proliferation, and EGFR-targeted therapies have shown significant clinical benefits in specific patient subsets.

**Fig. 7:**
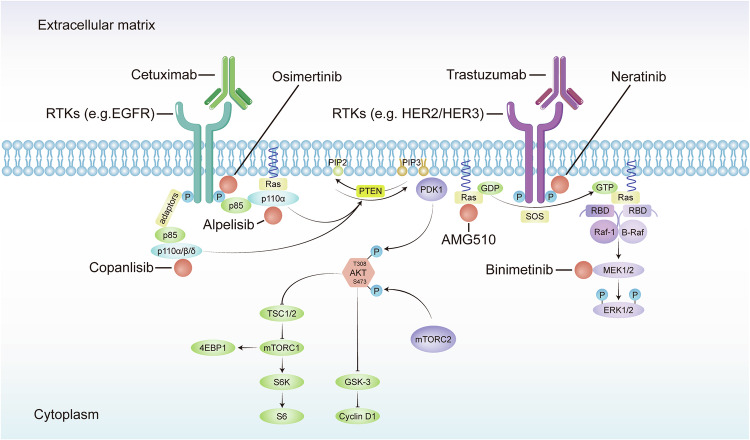
Colorectal cancer: therapeutic targeting of oncogenic signaling cascades. This figure illustrates key oncogenic signaling pathways in colorectal cancer, focusing on receptor tyrosine kinases (RTKs) such as EGFR and HER2/HER3, and their downstream cascades. It highlights the targeted therapies Cetuximab and Osimertinib for EGFR, Trastuzumab and Neratinib for HER2/HER3, AMG510 for KRAS, Binimetinib for MEK1/2, as well as Alpelisib and Copanlisib, which target the PI3K pathway by inhibiting the PI3K p110α subunit. These inhibitors disrupt critical signaling cascades (Ras/Raf/MEK/ERK and PI3K/AKT/mTOR pathways) involved in cell proliferation, growth, and survival, demonstrating their potential effectiveness in treating colorectal cancer

The RAS/RAF/MEK/ERK pathway, also known as the MAPK pathway, is another critical signaling cascade that regulates cell division, survival, and differentiation. Mutations in this pathway, especially in genes such as KRAS, NRAS, and BRAF, are prevalent in various malignancies. Inhibitors targeting different nodes of this pathway have been developed, with some showing success in treating cancers with specific genetic alterations (Fig. 7).

By targeting these and other essential pathways, targeted therapies offer a more precise and effective means to combat the complex disease that is cancer. The ongoing identification and validation of novel targets through genomic and proteomic approaches continue to expand the repertoire of targeted agents, providing hope for improved outcomes in cancer care.

### Targeting the Wnt/β-catenin signaling pathway

#### Porcupine inhibitors

Porcupine is an O-acyltransferase required for the palmitoylation and subsequent secretion of Wnt proteins. Inhibitors of Porcupine, such as LGK974 and ETC-159, block the secretion and activity of all Wnt ligands, thus preventing the activation of Wnt/β-catenin signaling in cancer cells.^249^ These agents are currently being investigated in clinical trials for their efficacy against Wnt-dependent tumors.

#### Frizzled receptor antagonists

Frizzled receptors are the cell-surface receptors for Wnt ligands. Antagonists of Frizzled receptors, like vantictumab and ipafricept (OMP-54F28), aim to inhibit the Wnt signaling at the membrane level, preventing downstream pathway activation.^250^ These antagonists are designed to disrupt the ligand-receptor interaction that is critical for pathway activation.

#### β-catenin inhibitors and degraders

Given the central role of β-catenin in the Wnt signaling pathway, direct inhibition of β-catenin is a rational therapeutic approach. Small molecule inhibitors, such as PRI-724, disrupt the interaction between β-catenin and its transcriptional coactivators, thereby inhibiting the expression of Wnt target genes. Proteolysis-targeting chimeras (PROTACs) that promote the degradation of β-catenin are also under development as a novel therapeutic strategy to target this pathway.^251^

#### Clinical trial updates on wnt pathway inhibitors

Several Wnt pathway inhibitors are currently undergoing clinical evaluation. As of the most recent updates, LGK974 has shown some promise in patients with Wnt-ligand dependent malignancies, with acceptable safety profiles.^252^ Clinical trials involving Frizzled receptor antagonists have also been initiated, and preliminary results suggest that these agents are well-tolerated with potential anti-tumor activity in patients with advanced solid tumors.^253^

#### Challenges in targeting the Wnt pathway and drug resistance mechanisms

Despite the clear rationale for targeting the Wnt/β-catenin pathway, clinical development of Wnt inhibitors has faced several challenges. One major hurdle is toxicity due to the pathway’s role in normal adult tissue homeostasis and regeneration. Additionally, drug resistance mechanisms have emerged, such as compensatory upregulation of alternative Wnt ligands and receptors or mutations in downstream components of the pathway.^254^ Understanding these resistance mechanisms is crucial for the development of next-generation Wnt inhibitors and combination therapies that can provide durable clinical responses.

### Inhibition of the EGFR signaling pathway

The EGFR, or ERBB1, is a transmembrane receptor tyrosine kinase that belongs to the ERBB protein family, which encompasses three additional members: HER2/ERBB2, HER3/ERBB3, and HER4/ERBB4. Binding of ligands such as EGF and TGFα to EGFR triggers conformational changes and activates its intrinsic kinase domain, promoting downstream signaling cascades such as the RAS/RAF/MEK/ERK axis and the PI3K/AKT pathway. These pathways are fundamental for cellular processes including proliferation, survival, and motility (Fig. 7).

The ERBB receptor network has been implicated in the etiology and progression of numerous solid tumors. Dysregulated EGFR signaling, often due to receptor overexpression or mutation, has been shown to enhance oncogenesis by fostering uncontrolled cell division, impeding programmed cell death, and facilitating invasion and metastasis. Seminal work by Masui et al. demonstrated the potential for EGFR-targeted therapeutics in cancer treatment, specifically highlighting the efficacy of anti-EGFR agents in inhibiting the proliferation of epidermoid carcinoma cells.^255^ Subsequently, research has focused on two categories of EGFR antagonists: monoclonal antibodies such as cetuximab and panitumumab, and tyrosine kinase inhibitors.^256^ In summary, targeted interventions against the EGFR signaling axis have reshaped the therapeutic landscape for mCRC and continue to be refined based on molecular markers, offering precision medicine approaches to improve patient outcomes.

Cetuximab is a chimeric IgG1 monoclonal antibody targeting EGFR, which gained FDA approval in 2004 for metastatic colorectal cancer (mCRC) management either in combination with irinotecan following failure of irinotecan-based chemotherapy or as monotherapy for irinotecan-intolerant patients. This approval was predicated on a pivotal trial that reported improved response rates (RR) and progression-free survival (PFS) when cetuximab was administered alongside irinotecan.^257^ The CRYSTAL study further established cetuximab’s efficacy in combination with FOLFIRI chemotherapy as a first-line treatment, particularly in patients with KRAS wild-type mCRC, though overall survival (OS) benefits were not observed in this cohort. The phase II OPUS trial echoed these findings, with cetuximab augmenting the effectiveness of FOLFOX4 in KRAS wild-type patients.^258^ A meta-analysis of the CRYSTAL and OPUS trials underscored the significant survival advantage for KRAS wild-type patients receiving cetuximab plus chemotherapy over chemotherapy alone.^259^ Recently, the FDA approved the combination of encorafenib and cetuximab for BRAF^V600E mutant mCRC based on the BEACON CRC trial, which demonstrated a median OS benefit.^260^

Panitumumab, a fully humanized IgG2 monoclonal antibody against EGFR, exhibits a high binding specificity and a lower immunogenic profile compared to cetuximab. Panitumumab, as a monotherapy or in combination with chemotherapy, has demonstrated efficacy in improving PFS in mCRC patients, particularly those with wild-type KRAS genotype, as reported in phase III trials.^261^ The FDA has sanctioned panitumumab both as a single-agent treatment post-chemotherapy failure and in combination with FOLFOX for front-line treatment in mCRC with wild-type KRAS.^262^ (Table 1).

**Table 1:** The exon localization, EGFR mutations, expected effect on anti-EGFR therapy effectiveness in which they have been described at least once

Exon	Mutation	Domain	Drug	Effect on EGFR/Therapy	Reference
12	R451C	III (ECD)	mAb^a^	Resistance	Arena et al.^531^
	S464L	III (ECD)	mAb^a^	Resistance	Arena et al.^531^
	G465E	III (ECD)	mAb^a^	Resistance	Siravegna et al.^532^
	G465R	III (ECD)	mAb^b^	Resistance	Arena et al.^531^
	K467T	III (ECD)	mAb^b^	Resistance	Arena et al.^531^
	I491M	III (ECD)	mAb^b^	Resistance	Arena et al.^531^
	S492R	III (ECD)	mAb^b^	Resistance	Montagut et al.^275^
18	E709K	TKD (ICD)	mAb^a^	Sensitivity	Kim et al.^533^
	G719A	TKD (ICD)	mAb^c^	Sensitivity	Bruera et al.^534^
	G719S	TKD (ICD)	mAb^b^	Sensitivity	Kim et al.^533^
	G724S	TKD (ICD)	mAb^b^	Sensitivity	Cho et al.^535^
19	E749K	TKD (ICD)	TKI^d^	Sensitivity	Zhang et al.^536^
20	T790M	TKD (ICD)	TKI^e^	Sensitivity	Buzard et al.^537^
22	E884K	TKD (ICD)	TKI^d^	Sensitivity	Deihimi et al.^538^

^a^Cetuximab/panitumumab

^b^Cetuximab

^c^Panitumumab

^d^Gefitinib

^e^Osimertinib

#### Resistance to anti-EGFR therapeutics

The phenomenon of drug resistance encompasses intrinsic (primary) and extrinsic (acquired) resistance to targeted therapies. Intrinsic resistance might arise from genetic mutations, loss of heterozygosity, or gene amplification, which can negate or attenuate the efficacy of the therapeutic targets. Notably, mutations in genes such as RAS, BRAF, and PIK3CA, as well as loss of PTEN and amplification of HER2, have been implicated in intrinsic resistance to anti-EGFR agents. Approximately 40% of patients with mCRC are estimated to benefit from anti-EGFR monoclonal antibodies (mAbs), underscoring the clinical imperative to identify biomarkers predictive of treatment response.^257^ Extrinsic resistance, on the other hand, involves acquired mutations like EGFR (S492R), as well as genomic alterations in RAS, BRAF, HER2, and MET, along with the selection of preexisting subclones that are intrinsically resistant to anti-EGFR mAbs. These mechanisms have been extensively reviewed in the literature.

#### Primary resistance

Mutations in the RAS gene occur in an estimated 40% of CRC cases, with these aberrations leading to constitutive activation of the MAPK signaling pathway and subsequent resistance to anti-EGFR mAbs.^263^ Mutations in exon 2 (codons 12 and 13) of KRAS are particularly significant, accounting for 85–90% of RAS mutations in CRC, and are present in ~40% of mCRC cases.^262^ Numerous studies have demonstrated that patients harboring KRAS exon 2 mutations do not benefit from anti-EGFR mAbs and suffer from decreased PFS, OS, and RR compared to those with wild-type KRAS.^264^ In 2009, the FDA restricted the use of anti-EGFR mAbs to patients with mCRC harboring wild-type KRAS exon 2.^265^ Additional mutations in KRAS exons 3 and 4, as well as mutations in NRAS, have been identified in 15–20% of patients who are wild-type for KRAS exon 2 and are similarly associated with reduced PFS and OS.^263^

BRAF mutations, particularly the V600E variant, which accounts for the majority of BRAF alterations, result in direct activation of the RAS/RAF/ERK signaling pathway and confer resistance to anti-EGFR mAbs. Although BRAF V600E mutations have been associated with poorer clinical outcomes in patients receiving anti-EGFR mAbs,^266^ evidence regarding its role as a predictive biomarker remains inconclusive. Indeed, a comprehensive meta-analysis conducted by Rowland and colleagues revealed no significant disparity in OS and PFS between patients with wild-type RAS/BRAF and those harboring mutations in these genes. The findings of this meta-analysis suggest a lack of conclusive evidence to assert that individuals with RAS wild-type/BRAF mutant (RAS WT/BRAF MT) phenotypes experience differing therapeutic outcomes from anti-EGFR mAbs in mCRC compared to those with both RAS and BRAF wild-type (RAS WT/BRAF WT).^267^ However, a separate meta-analysis by Lu et al. reached different conclusions. In their study of RAS wild-type (WT) mCRC patients undergoing EGFR-targeted therapy, they identified the BRAF mutation as a significant prognostic and predictive biomarker. Conversely, mutations in PIK3CA, PTEN, or deletions in these genes did not exhibit a substantial effect. However, a composite biomarker profile consisting of KRAS/NRAS/BRAF/PIK3CA mutations was predictive of resistance to anti-EGFR therapy.^268^ In addition, the BEACON CRC study has established encorafenib combined with cetuximab as a new standard of care for previously treated BRAF V600E-mutant mCRC. The regimen significantly improved OS, objective response rate (ORR), and PFS compared to standard chemotherapy, as evidenced by both the initial and updated results. Therefore, although different reports debating the prognostic value to BRAF mutation, encorafenib plus cetuximab should be considered a preferred therapeutic option for this patient subgroup, marking a significant advancement in the management of this challenging oncological condition.^260^

PIK3CA mutations and PTEN loss are also implicated in resistance to anti-EGFR therapy. The PI3K-AKT-mTOR pathway, which can be activated by EGFR, is crucial in cell survival and proliferation in CRC. PIK3CA mutations, which occur in 10–18% of CRC cases, activate the PI3K/AKT pathway, thereby conferring resistance to anti-EGFR mAbs.^269^ Mutations in PIK3CA exons 9 and 20, which constitute the majority of these mutations, lead to pathway activation. Clinical resistance to cetuximab and panitumumab in the presence of PIK3CA mutations was reported by Sartore-Bianchi and colleagues.^270^ Yet, a large retrospective analysis posited that only mutations in exon 20 of PIK3CA are associated with reduced RR, PFS, and OS in response to combined cetuximab and chemotherapy, unlike exon 9 mutations.^271^ A meta-analysis by Mao et al. suggested that exon 20 mutations in PIK3CA may predict resistance to anti-EGFR mAbs in patients with wild-type KRAS mCRC, although the evidence is not definitive due to sample size limitations.^272^

PTEN, a tumor suppressor gene, negatively regulates the PI3K-AKT-mTOR pathway. Loss of PTEN expression or function results in continuous pathway activation, contributing to uncontrolled cell growth. While some studies suggest PTEN could be a predictive biomarker for response to anti-EGFR therapy, particularly in patients with wild-type KRAS,^88^ other research has not found a significant correlation between PTEN status and therapeutic response.^269^ Further large-scale clinical studies are necessary to clarify the role of PTEN in anti-EGFR resistance.

#### Emergence of acquired resistance

Development of KRAS Mutations: It is well-established that the RAS/RAF signaling axis is a critical determinant in mediating primary resistance to anti-EGFR mAbs in mCRC, as well as in the emergence of secondary resistance.^5^ Diaz et al. observed mutations in KRAS in ~38% of patients with initially KRAS wild-type status following treatment with panitumumab, typically manifesting after a median treatment duration of 5 to 6 months.^273^ Intriguingly, a computational model derived from the study indicated the pre-existence of KRAS-resistance mutations within minor clonal populations prior to the commencement of panitumumab therapy.^273^ Complementary findings by Morelli et al. demonstrated that 55% of patients who acquired resistance to cetuximab or panitumumab had secondary KRAS mutations, with a smaller fraction (9%) exhibiting KRAS gene amplification.^274^ Furthermore, circulating tumor DNA analysis revealed the presence of KRAS variants in patients up to 10 months before radiographic disease progression became evident.^274^

Alterations in EGFR S492R: First described by Montagut and colleagues in 2012, the S492R mutation within EGFR was implicated in conferring resistance to cetuximab therapy in mCRC patients, a phenomenon not paralleled in those treated with panitumumab.^275^ Their investigation revealed that 20% of cetuximab-resistant patients possessed this mutation.^275^ A subsequent expansive study involving 505 mCRC patients with KRAS exon 2 wild-type genotype suggested that the EGFR S492R mutation was not associated with primary resistance to cetuximab.^276^ Evidence from the ASPECCT trial, a phase III comparative analysis, indicated that 16% of cetuximab-treated patients developed this mutation, as determined by liquid biopsy, compared to only 1% of those receiving panitumumab.^277^

*HER2 amplification:* HER2, an activator of the RAS/RAF/ERK and PI3K/AKT signaling cascades via dimerization with EGFR and HER3, is a member of the ERBB receptor tyrosine kinase family. Its status has been investigated as a potential marker for anti-EGFR therapy response.^278^ Research involving patient-derived xenografts indicates that HER2 amplification or the heightened expression of its ligand, heregulin, induces resistance to cetuximab, especially in tumors lacking KRAS and BRAF mutations.^279^ Clinical corroboration comes from a comprehensive retrospective study by Martin and associates, which highlights that HER2 gene amplification may be linked to anti-EGFR therapeutic resistance in KRAS wild-type mCRC. While HER2 amplification is a relatively infrequent event, affecting about 2% of mCRC cases, it is predominantly recognized as a mechanism of secondary rather than primary resistance.^280^

*MET amplification dynamics:* The proto-oncogene MET encodes the receptor tyrosine kinase c-MET, which upon binding to hepatocyte growth factor (HGF), triggers signaling pathways such as PI3K/AKT, RAS/RAF/ERK, STAT3, and NF-κB, promoting cellular growth and survival. Liska and colleagues reinforced the role of MET activation in reactivating MAPK and AKT signaling in the context of CRC and anti-EGFR treatment resistance.^281^ Bardelli et al. provided evidence that MET amplification, detectable in circulating tumor DNA, can signal the onset of acquired resistance to anti-EGFR therapy in KRAS wild-type cases prior to clinical relapse.^282^ Additionally, research by Troiani et al. implicated TGF-α-mediated EGFR-MET interaction in the induction of MET-driven resistance, suggesting that MET inhibition could restore cetuximab sensitivity.^283^ Despite its relevance, the infrequency of MET amplification in mCRC (approximately 1%) renders it a limited predictive biomarker for primary resistance to anti-EGFR therapies.

*Subclonal selection and resistance:* Secondary resistance in mCRC is not solely attributed to the occurrence of new genetic alterations during treatment but may also involve the selective outgrowth of preexisting subclones that are inherently resistant to targeted therapies.^273^ Analyses by Misale et al. of the gene copy number and mutational landscape of both parent and cetuximab-resistant cell lines indicated the presence of mutations such as KRAS G13D and gene amplification within low-frequency parental cell populations, suggesting a subclonal selection process.^5^ However, other alterations, including KRAS G12R and EGFR S492R mutations, were identified exclusively in resistant cells, supporting the notion that certain mutations may arise as a direct consequence of anti-EGFR mAb treatment.^5^

### Targeting the RAS/RAF/MEK/ERK signaling cascade

#### RAS inhibitors and the challenge of directly targeting RAS

RAS proteins (HRAS, KRAS, and NRAS) are small GTPases that function as molecular switches in various signal transduction pathways. Oncogenic mutations in RAS genes, particularly KRAS, are among the most common alterations in human cancers and have historically been considered “undruggable” due to the lack of suitable pockets for small-molecule binding. Recent advancements, however, have led to the development of covalent inhibitors that target specific KRAS mutations, such as the KRAS^G12C inhibitors, which show promising preclinical and early clinical activity^284^ (Table 2). Sotorasib has been lauded as a groundbreaking agent in the targeted therapy landscape, particularly given its specificity for the KRAS^G12C mutation. In CRC, the presence of the KRAS^G12C mutation, albeit less prevalent than in NSCLC, signifies a poor prognosis and presents a challenge for effective treatment. Recent clinical trials, such as the CodeBreaK100 phase II study, have demonstrated that Sotorasib can achieve disease control and extend progression-free survival in CRC patients harboring this specific mutation.^285^ Despite these advances, the response rates in CRC appear lower than those observed in NSCLC, suggesting that intrinsic differences in KRAS^G12C-driven signaling or the TME may influence therapeutic efficacy.

**Table 2:** Reported clinical trials of KRAS G12C inhibitors in metastatic colorectal cancer

Trial name	Phase	drug	Tumor type	Number of patients	Regimen	ORR (%)	DCR (%)	Median PFS (months)	Median overall survival (months)
CodeBreaK 300, (NCT05198934)	III	sotorasib	advanced CRC with KRAS G12C mutation	53patients 53patients 54patients	Sotorasib (960 mg,po,qd) plus panitumumab, Sotorasib (240 mg,po,qd) plus panitumumab, the investigator’s choice of trifluridine-tipiracil or regorafenib	26.4%; 5.7% 0%		5.6 m (4.2–6.3) ; 3.9 m (3.7–5.8) ; 2.2 m (1.9–3.9)	maturing
CodeBreaK 101(NCT04185883)	1b	sotorasib	advanced mCRC with KRAS G12C mutation	48	Sotorasib (960 mg,po,qd) combine with panitumunab(6 mg/kg.iv.q2w) standard FOLFIRI(iv,q2w)	58.1%	93.5%	5.7 m	15.2 m
CodeBreaK100 (NCT03600883)	I/II	sotorasib	advanced mCRC with KRAS G12C mutation	62	Soto (960 mg,po,qd)	9.7%	82.3%	4.0 m	10.6 m
NCT03785249	I/IB	Adagrasib	advanced CRC with KRAS G12C mutation	44 pts ada 32 pts ada + cetux.	Adagrasib (600 mg BID) in combination with/notwith intravenous cetuximab (cetux:400 mg/m^2^ followed by 250 mg/m2 QW or 500 mg/m^2^ Q2W)	19% 46%	86% 100%	5.6 m 6.9 m	
NCT04585035	II	D-1553 (Garsorasib)	mCRC with KRAS G12C mutation	40	Garsorasib (600 mg BlD) plus Cetuximab (standard dose)	45.0%	95%	7.62 m	maturing
NCT05497336	1b	IBI351 (GFH925)	advanced m CRC with KRAS G12C mutation	42	600 mg BID	43.5%	87%		
Codebreak300 (NCT05194995)	I/II	JAB-21822+Cetuximab	advanced CRC, small intestine cancer and appendiceal cancer with KRAS G12C mutation	160	JAB 21822+Cetuximab	26.4%	72%	5.6 m	

#### RAF inhibitors (e.g., vemurafenib, dabrafenib) in BRAF-mutant cancers

Inhibitors of RAF kinases, particularly those targeting the BRAF^V600E mutation, have shown significant clinical benefit in melanoma and other cancers harboring this mutation. Vemurafenib and dabrafenib are selective BRAF inhibitors that have transformed the treatment landscape for patients with BRAF^V600E mutant melanoma, resulting in improved rates of response and overall survival.^286^ Their development underscores the importance of personalized medicine in oncology.

#### MEK inhibitors (e.g., trametinib, cobimetinib) and their clinical efficacy

MEK1 and MEK2 are downstream kinases in the MAPK pathway and are critical effectors of BRAF and RAS signaling. The MEK inhibitors trametinib and cobimetinib have demonstrated clinical efficacy, particularly in combination with BRAF inhibitors, for the treatment of BRAF-mutant melanoma.^287^ These combinations have been approved for use based on their ability to improve outcomes, including progression-free survival and overall survival, in these patients.

#### ERK inhibitors as emergent therapeutic agents

ERK1/2 are the terminal kinases in the MAPK cascade and have recently emerged as therapeutic targets with the development of ERK inhibitors. These inhibitors hold potential in overcoming resistance to upstream inhibitors and are currently being evaluated in clinical trials.^288^ Their place in the treatment algorithm of MAPK pathway-driven cancers remains to be fully elucidated.

#### Adaptive resistance and feedback loops in the MAPK pathway

Resistance to MAPK pathway inhibitors is a major clinical challenge and often arises through adaptive resistance mechanisms. These include reactivation of the pathway downstream of the blockade, activation of parallel survival pathways, and genetic alterations that bypass the inhibited kinase.^289^ Feedback loops within the MAPK pathway itself can also contribute to resistance, as inhibition of one component can lead to upregulation of upstream components due to relief of negative feedback. Understanding these resistance mechanisms is crucial for the development of next-generation inhibitors and combination strategies to improve patient outcomes.

### Additional targeted pathways and agents

#### PI3K/AKT/mTOR pathway inhibitors

PI3K/AKT/mammalian target of rapamycin (mTOR) pathway is another critical intracellular signaling pathway that is frequently dysregulated in cancer, promoting cell growth, survival, and metabolism. Inhibitors targeting various components of this pathway, including PI3K inhibitors (e.g., idelalisib), AKT inhibitors (e.g., ipatasertib), and mTOR inhibitors (e.g., everolimus), have shown clinical efficacy in different cancer types.^290^ Due to the complexity and redundancy of the PI3K/AKT/mTOR network, combination therapies are being evaluated to overcome resistance mechanisms and improve therapeutic outcomes.

#### CDK4/6 inhibitors in cell cycle regulation

Cyclin-dependent kinases 4 and 6 (CDK4/6) play a pivotal role in cell cycle progression by phosphorylating the retinoblastoma (Rb) protein. CDK4/6 inhibitors such as palbociclib, ribociclib, and abemaciclib have been approved for the treatment of hormone receptor-positive, HER2-negative advanced breast cancer.^291^ They are often used in combination with endocrine therapy and have significantly improved progression-free survival in this setting.

#### Targeting angiogenesis with VEGF inhibitors

Angiogenesis, the formation of new blood vessels, is crucial for tumor growth and metastasis. Vascular endothelial growth factor (VEGF) is a potent angiogenic factor, and its signaling can be inhibited by agents such as bevacizumab (a monoclonal antibody against VEGF), and tyrosine kinase inhibitors like sunitinib and sorafenib that target VEGF receptors.^292^ These agents have shown benefits in various cancers, including colorectal, lung, and renal cell carcinomas, and have become a cornerstone of anti-angiogenic therapy.^293^

#### Poly (ADP-ribose) polymerase (PARP) inhibitors in DNA repair pathways

PARP inhibitors exploit synthetic lethality by targeting DNA repair pathways. They are particularly effective in cancers characterized by BRCA1/2 mutations, which already have compromised homologous recombination repair mechanisms.^294^ PARP inhibitors such as olaparib, rucaparib, and niraparib have been approved for the treatment of ovarian and breast cancers with BRCA mutations, and their use is expanding to other cancers and settings.^295^

#### Other emerging targets and pathways of interest

Research continues to uncover a myriad of novel targets and pathways with therapeutic potential in oncology. These include targeting the TME, modulating the cancer epigenome, inhibiting the proteasome, and interfering with cancer metabolism. Agents targeting specific molecular aberrations, such as NTRK fusions and cMET alterations, have also shown promising activity.^296^ For patients with ntrk positive colorectal cancer, NCCN (2024, v1) guidelines recommend larotrectinib and entrectinib for treatment. Larotrectinib (vitrakvi) is an oral Trk inhibitor. For patients aged 4 months to 76 years, the overall remission rate of 17 different cancer treatments, including colorectal cancer, is 75%.^297^ Entrectinib, a selective tyrosine kinase inhibitor (TKI), was approved by FDA in 2019. Research shows that the remission rate of ntrk fusion positive solid tumors (including colorectal cancer) is 57%.^298^

RET is a receptor tyrosine kinase, which plays a key role in the development and maintenance of nerve and genitourinary tissues through downstream MAPK and PI3K signaling pathways. Somatic activation changes of RET include point mutation and gene rearrangement, and have been identified in a variety of tumors. In September 2022, FDA has accelerated the approval of retevmo (selpercatinib) for the treatment of locally advanced or mCRC patients with RET gene fusion. The total remission rate was 20%.^299^

The clinical application of these new targeted drugs brings a new dawn to the treatment of patients with colorectal cancer. As our understanding of cancer biology deepens, precision oncology is poised to offer increasingly individualized and effective treatment options.

### Overcoming therapeutic hurdles in targeted therapy

The advent of targeted therapies has heralded a new era in the treatment of malignancies. However, the clinical benefits are often transient due to inherent and acquired resistance mechanisms. Overcoming these therapeutic hurdles necessitates a multi-faceted approach that encompasses combination therapies, predictive biomarkers, adaptive dosing strategies, the development of next-generation inhibitors, and strategies to tackle tumor heterogeneity and clonal evolution.

#### Combination therapy strategies to prevent or overcome resistance

Combination therapy is a cornerstone strategy to prevent or overcome resistance to targeted therapies. This approach can involve the concurrent targeting of multiple pathways critical to tumor survival and proliferation. Preclinical studies have shown the efficacy of combining BRAF and MEK inhibitors in melanoma, leading to improved outcomes and a delay in the onset of resistance.^287^ Clinical trials evaluating the efficacy of combining immune checkpoint inhibitors with targeted therapies are also underway.^300^

#### Utilization of predictive biomarkers for therapy selection

The successful application of targeted therapy is contingent upon the presence of specific biomarkers that can predict therapeutic response. The identification of such biomarkers through comprehensive genomic profiling can guide the selection of appropriate targeted agents. For example, the presence of EGFR mutations in NSCLC predicts response to EGFR tyrosine kinase inhibitors.^301^ Biomarker-driven therapy selection remains an area of intense research and is critical for the realization of personalized medicine.

#### Adaptive dosing and schedule modulation

Adaptive dosing and schedule modulation are strategies that can mitigate the development of resistance and improve therapeutic index. By adjusting the dose and timing of drug administration based on real-time assessment of tumor response and patient tolerance, it is possible to maintain therapeutic efficacy while minimizing toxicity. Mathematical modeling and computational approaches are being utilized to optimize dosing regimens.^302^

#### Development of next-generation inhibitors with improved specificity

The development of next-generation inhibitors is focused on improving specificity and reducing off-target effects. These novel agents are designed to bind more selectively to mutated oncogenic proteins while sparing the wild-type counterparts, thereby enhancing efficacy and reducing toxicity. An example is the development of ALK inhibitors with increased potency and selectivity for the treatment of ALK-positive NSCLC.^303^

#### Approaches to target tumor heterogeneity and clonal evolution

Tumor heterogeneity and clonal evolution po.se significant challenges to targeted therapy, as subpopulations of cancer cells may harbor distinct genetic profiles that confer resistance. Single-cell sequencing and spatial multi-omics are being employed to unravel the complexity of heterogeneous tumors. Strategies to target this heterogeneity include the development of broad-spectrum inhibitors, the use of combination therapies that target multiple clonal populations simultaneously, and the implementation of adaptive therapy protocols that anticipate and respond to clonal evolution.^304^

In conclusion, overcoming the obstacles posed by targeted therapy resistance requires an integrated approach that combines advanced molecular diagnostics, innovative drug development, and strategic treatment regimens. As our understanding of the molecular underpinnings of cancer continues to grow, so too will our ability to devise effective interventions that improve patient outcomes.

## The immunological landscape and immunotherapy in CRC

### The interface between the immune system and CRC

The innate immune system, the body’s first line of defense, encompasses a variety of cell types, including natural killer (NK) cells, macrophages, dendritic cells (DCs), and granulocytes, which collectively mount an immediate response to malignancies such as CRC. NK cells, for instance, are critical due to their ability to recognize and destroy tumor cells without prior sensitization. Their cytotoxic activity is mediated by the release of perforin and granzymes, which induce apoptosis in target cells.^305^ Nonetheless, CRC can evade NK cell-mediated lysis through the expression of immune checkpoint molecules or by altering NK cell receptors’ expression patterns.^306^

Macrophages exhibit remarkable plasticity, and within the TME, they can differentiate into tumor-associated macrophages (TAMs) that often promote tumorigenesis. Phenotypically, these TAMs resemble M2 macrophages, which are associated with tissue repair and immunosuppression, rather than the tumoricidal M1 phenotype.^307^ TAMs can facilitate tumor growth through the production of growth factors, promotion of angiogenesis, and suppression of adaptive immunity.

Dendritic cells are pivotal in bridging the innate and adaptive immune systems by presenting antigens to T cells, thus initiating specific immune responses. Within CRC, the function of DCs is often compromised; they are rendered tolerogenic, leading to decreased T cell activation and a dampened immune response.^308^

Granulocytes, including neutrophils, eosinophils, and basophils, can also infiltrate tumors. Their role in CRC is complex, with some evidence suggesting that they may contribute to tumor growth and metastasis through the release of proteases, reactive oxygen species, and cytokines.^309^

The adaptive immune system is characterized by its antigen-specific responses and the development of immunological memory. T lymphocytes, particularly CD8+ cytotoxic T cells, are crucial in controlling tumor growth by directly killing cancer cells. The presence of tumor-infiltrating lymphocytes (TILs), especially CD8 + T cells, in CRC is often indicative of a better prognosis. CD4 + T helper cells are also essential as they aid in activating other immune cells, including B cells and macrophages.^310^

B lymphocytes contribute to the adaptive immune response against CRC through antibody production and antigen presentation. These antibodies can directly target tumor cells or opsonize them for phagocytosis by macrophages and other phagocytic cells.^311^

Tregs, a subset of CD4 + T cells, are known to maintain immune tolerance and prevent autoimmunity. However, in the context of CRC, they can also suppress effective anti-tumor immunity and are correlated with poor clinical outcomes.^312^

The roles of innate and adaptive immune responses in CRC are multifaceted, with each arm of the immune system contributing to the surveillance and potential elimination of tumor cells. The efficacy of these responses can be heavily influenced by the TME, which can either enhance or suppress immune activity. A deeper understanding of these mechanisms is crucial for the development of more effective immunotherapies for CRC.

### Immune surveillance and tumor escape mechanisms

The delicate equilibrium between immune surveillance and tumor escape is a central theme in cancer biology. The complex interplay between evolving neoplastic cells and the host’s immune system can dictate the trajectory of tumor progression and patient prognosis. This dynamic process is well exemplified in CRC, where both innate and adaptive immune mechanisms are actively engaged in recognizing and eliminating malignant cells, while the tumor develops strategies to evade these responses.

Immunoediting is a term that describes the dual role of the immune system in both protecting against tumors by eradicating cancer cells and sculpting the immunogenicity of tumors by selecting for less detectable cell variants. In CRC, this concept is manifested through three distinct phases: elimination, equilibrium, and escape.^313^ Elimination: This initial phase involves the recognition and destruction of nascent tumor cells by immune effector cells. In CRC, this is mediated by the cytotoxic effects of NK cells, CTLs, and γδ T cells, supported by the pro-inflammatory cytokine milieu. Equilibrium: As the immune system exerts pressure, tumor cell populations that survive can enter a state of dynamic equilibrium, wherein immune-mediated selection pressures lead to the outgrowth of tumor variants that are less immunogenic and more capable of resisting immune attack. Escape: In this final phase, immune-resistant tumor cells proliferate and may even manipulate the immune system to facilitate growth and metastasis. Mechanisms of immune escape in CRC include the loss of tumor antigenicity, secretion of immunosuppressive cytokines (e.g., TGF-β, IL-10), recruitment of suppressive cell populations (e.g., Tregs, myeloid-derived suppressor cells), and alteration of antigen processing and presentation machinery.^314^

### The role of immune checkpoints in immune evasion

Immune checkpoints are pivotal regulators of immune responses, maintaining self-tolerance and modulating the duration and amplitude of physiological immune responses in peripheral tissues. In CRC, cancer cells exploit these checkpoints to avoid immune destruction.

#### CTLA-4

Cytotoxic T-lymphocyte-associated protein 4 (CTLA-4) is an inhibitory receptor expressed by T cells. Its role in immune evasion is primarily attributed to its competition with the costimulatory receptor CD28 for binding to CD80 and CD86 on antigen-presenting cells, thus dampening T cell activation.^315^

#### PD-1/PD-L1

Programmed cell death protein 1 (PD-1) and its ligand PD-L1 are perhaps the most well-characterized checkpoint inhibitors in CRC. The interaction between PD-1 on T cells and PD-L1 on tumor cells leads to T cell exhaustion and anergy. PD-L1 expression can be induced on tumor cells and immune cells within the TME, constituting a formidable barrier to anti-tumor immunity.^316^

#### Other checkpoints

Additional checkpoints such as LAG-3, TIM-3, and TIGIT are also implicated in immune regulation within the TME of CRC. These molecules, either alone or in combination, contribute to a complex network of inhibitory signals that cancer cells can leverage to suppress effective immune responses.^317^

The intricate balance between immune surveillance and tumor escape is a defining feature of the host-tumor interaction in CRC. Understanding the mechanisms of immunoediting and the role of immune checkpoints in immune evasion is crucial for developing novel therapeutic strategies, including checkpoint blockade and other forms of immunotherapy, which aim to tilt the balance in favor of the host’s immune system.

### Immune checkpoint inhibitors

**Fig. 8:**
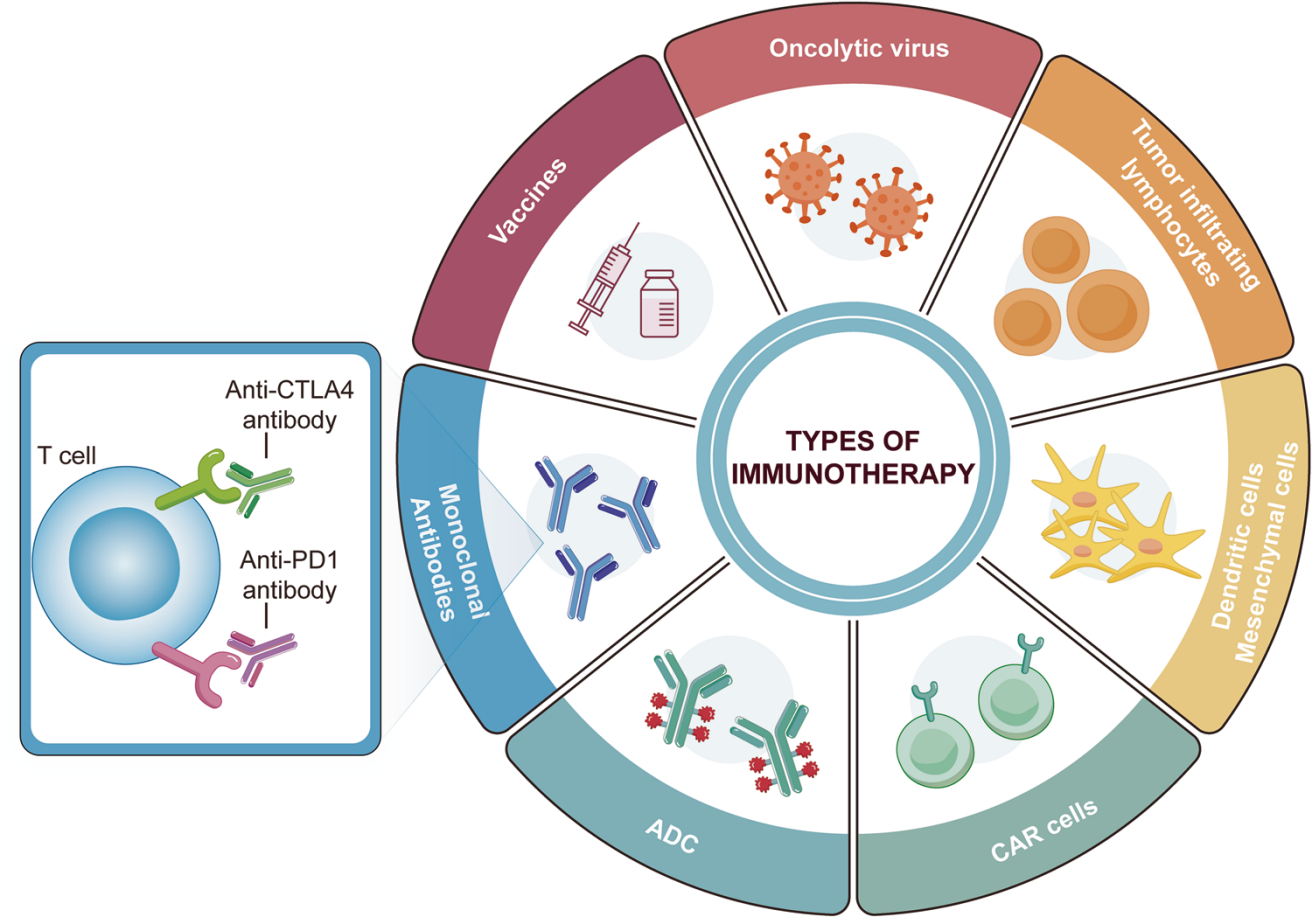
Immunotherapeutic strategies for CRC. This panel illustrates various immunotherapeutic strategies for colorectal cancer. The central circle highlights the different types of immunotherapy, including Vaccines, Oncolytic virus, Tumor-infiltrating lymphocytes, Dendritic cells/Mesenchymal cells, CAR cells, ADC, and Monoclonal antibodies. The inset on the left depicts the mechanism of action of T cells, highlighting the roles of Anti-CTLA4 and Anti-PD1 antibodies in targeting cancer cells

Immune checkpoint inhibitors (ICIs) are a class of monoclonal antibodies that target immune checkpoint proteins such as CTLA-4, PD-1, and its ligand PD-L1. These proteins play critical roles in maintaining immune homeostasis but can be co-opted by tumors to evade immune surveillance (Fig. 8).

Pembrolizumab, a humanized monoclonal antibody targeting the PD-1, is designed to disrupt the interaction between PD-1 and its ligands, PD-L1 and PD-L2. This blockade enhances the ability of cytotoxic T cells to recognize and destroy tumor cells. Initially approved by the FDA in 2016, pembrolizumab was sanctioned for use in patients with PD-L1-expressing metastatic NSCLC.^318^ By 2020, its application was extended to include treatment for patients with unresectable or MSI-H mCRC who had not previously received systemic treatment for advanced disease.^319^ A multitude of clinical trials, both completed and ongoing, have been conducted to assess the impact of pembrolizumab on advanced CRC. The phase II KEYNOTE-164 trial, for example, demonstrated notable antitumor activity of pembrolizumab in MSI-H mCRC, affirming its therapeutic benefit.^320^ Interestingly, only a minority (2.85%) of patients developed antibodies against pembrolizumab, which might impede the drug’s efficacy.^321^ Nonetheless, variability in response and instances of resistance have been noted, possibly due to the immunosuppressive actions of certain immune cells within the TME. To counteract this, strategies involving the use of pembrolizumab in combination with other immunotherapeutics are being explored to transform the TME from a ‘cold’ to a ‘hot’ state, thereby enhancing T-cell responses.^322^

Research by Herting et al. investigated the safety and efficacy of combining pembrolizumab with standard FOLFOX chemotherapy in treating mCRC. While safe, this combination did not significantly improve median PFS or OS compared to chemotherapy alone. Analysis of immune responses revealed that lower levels of TNF-α were associated with better clinical outcomes, whereas higher levels of Flt3 ligand and TGF-α were linked to improved PFS.^323^ Additional studies have focused on innovative combinations, such as pembrolizumab with the GVAX colon vaccine, which aims to alter the TME and boost TILs. Though ORR did not markedly change, a significant reduction in tumor markers was observed.^324^ Another approach involved the CCR5 inhibitor maraviroc, known to regulate the recruitment of immunosuppressive M2 macrophages, in combination with pembrolizumab. This regimen resulted in increased levels of antitumor chemokines and was correlated with improved OS.^325^ Further exploratory combinations have included AMG 820, a colony-stimulating factor 1 receptor inhibitor, and NOX-A12, a CXCL12 inhibitor, both of which have shown potential in modulating the TME and enhancing immune responses.^326^ The latter study revealed changes in cytokine profiles conducive to a favorable inflammatory cell milieu, with tissue responders exhibiting increased levels of inflammatory cytokines and higher numbers of activated CD3 + T cells.^326^

Avelumab is a humanized anti-PD-L1 antibody that inhibits the interaction of PD-1 receptors with B7-1 on T cells, while simultaneously promoting antibody-dependent cellular cytotoxicity through its engineered Fc gamma receptor 1. The FDA has sanctioned avelumab for treating metastatic Merkel cell carcinoma and locally advanced or metastatic urothelial carcinoma. A correlation between immune-related adverse events (AEs) and enhanced survival has been noted in patients undergoing avelumab treatment.^327^ The safety profile and efficacy of combining avelumab with autologous DCs in mCRC patients indicated a well-tolerated regimen, with a PFS of 3.1 months and an OS of 12.2 months.^328^

Nivolumab, a potent ICI targeting the PD-1 receptor on activated T cells, is a human monoclonal immunoglobulin (Ig) G4 antibody. It binds with high affinity to its receptor, blocking the PD-1 interaction with its ligands (PD-L1 and PD-L2) on tumor cells, thereby rejuvenating T-cell activity and fostering anti-tumor immune responses.^329^ Nivolumab was approved by the FDA in 2014 for advanced melanoma and subsequently for additional malignancies such as NSCLC, renal cell carcinoma, and Hodgkin’s lymphoma, and CRC.^330^ Ongoing and completed clinical studies have explored nivolumab both as a standalone treatment and in combination with other therapeutic agents for CRC, especially in advanced or metastatic settings. The FDA’s accelerated approval of nivolumab in July 2017 for the second-line treatment of microsatellite instability-high/mismatch repair-deficient (MSI-H/dMMR) CRC was based on robust data from phase II clinical trials.^331^ The CheckMate142 study (NCT02060188) specifically tested nivolumab’s efficacy in dMMR/MSI-H mCRC patients, confirming its safety profile consistent with previous studies on other solid tumors, with no new safety concerns identified.^332^ These results led to FDA approval for the treatment of dMMR/MSI-H mCRC in adults and children over 12 years. Additionally, the combination of nivolumab and ipilimumab received accelerated approval for treating refractory MSI-H/dMMR CRC, following evidence from the CheckMate142 study that suggested clinical benefits from combined ICIs in this patient subgroup.^333^

The advent of nivolumab has markedly altered the therapeutic landscape for multiple cancers, including dMMR/MSI-H CRC, enhancing patient outcomes and extending survival. Nevertheless, not all cases of dMMR/MSI-H CRC are responsive to immunotherapy, with primary resistance observed in about 50% of patients, underscoring the molecular heterogeneity within dMMR/MSI-H CRC.^334^ Some subtypes of CRC display limited sensitivity to current immunotherapies, highlighting a critical need to transform these less responsive CRC subtypes into highly immunogenic tumors akin to MSI-H CRC. Continued research and clinical trials are essential to fully realize the potential of nivolumab in CRC therapy.

Atezolizumab, a humanized IgG1 monoclonal antibody targeting PD-L1, has garnered FDA approval for the management of metastatic NSCLC following the failure of platinum-based chemotherapies. Initial explorations into the efficacy of atezolizumab were conducted through a phase I trial that encompassed patients with various advanced malignancies, including NSCLC, melanoma, gastric cancer (GC), renal cell carcinoma, head and neck squamous cell carcinoma, and CRC.^335^ Continuing investigations are assessing atezolizumab’s effectiveness in CRC through several trials, with a particular focus on its combination with capecitabine and bevacizumab. This combination, however, has demonstrated limited clinical benefits. Notably, the concurrent inhibition of the VEGF alongside PD-1 or PD-L1 pathways has shown enhanced efficacy in patients with microsatellite-stable (MSS) and mismatch repair-proficient (pMMR) tumors, especially in those without liver metastases.^336^ Additionally, when combined with the FOLFOXIRI/bevacizumab regimen, atezolizumab led to extended PFS in patients with metastatic CRC (mCRC). The therapeutic benefits appear more pronounced in patients with dMMR tumors. However, the results from the completed clinical trial IMblaze370 indicated that the combination treatments involving atezolizumab with cobimetinib or regorafenib did not enhance OS in patients. The safety profiles of these combinations were similar to those observed when the drugs were administered independently, underscoring the challenges in amplifying the benefits of immunotherapy in tumors with low baseline immune inflammation.^337^

Collectively, these efforts underscore the potential of atezolizumab to enhance the immune response against tumors and curb the progression and metastasis of cancer cells, particularly when used in conjunction with chemotherapy and other therapeutic agents. In CRC, ICIs have demonstrated significant efficacy, particularly in patients with mismatch repair-deficient/microsatellite instability-high (dMMR/MSI-H) CRC, which are characterized by a high mutational burden that generates neoantigens, enhancing the immune system’s ability to recognize and attack tumor cells. The KEYNOTE-177 trial, a pivotal study, established pembrolizumab as a first-line treatment for patients with dMMR/MSI-H metastatic CRC, showing superior progression-free survival compared to standard chemotherapy.^338^

Durvalumab, a monoclonal antibody targeting the PD-1/PD-L1 axis, has been sanctioned by the FDA for use in multiple oncological conditions. Current research illustrates durvalumab’s potential through favorable clinical outcomes, particularly notable in mCRC patients with MSI-H/dMMR or mutations in the exonuclease domain of polymerase epsilon. Notably, the therapeutic response in polymerase epsilon-mutated mCRC appears predominantly in patients exhibiting dMMR characteristics.^339^ Additionally, a study highlighted the immune activation of T and B cells in pMMR mCRC patients treated with a neoadjuvant combination of durvalumab and tremelimumab prior to liver resection.^340^ Further inquiries have assessed the safety and efficacy of PexaVec combined with durvalumab and tremelimumab, demonstrating promising results in pMMR mCRC, though calling for further research to identify reliable predictive biomarkers.^341^ Another investigation evaluated the addition of bevacizumab and FOLFOX to a regimen of durvalumab and oleclumab; while this combination improved response rates, it failed to extend progression-free survival beyond standard treatments.^342^ One phase II trial examined the efficacy of durvalumab and tremelimumab with palliative hypofractionated radiotherapy in MSS mCRC, confirming the safety and tolerability of this immunotherapeutic combination.^343^ Another study assessed the same combination with radiotherapy for inducing systemic antitumor immunity but did not achieve its primary endpoints, though rare systemic immune enhancements and reductions in nonirradiated lesions suggested potential abscopal effects.^344^ The safety of integrating Y90 radioembolization with durvalumab and tremelimumab treatment was also tested; however, this combination did not provoke significant tumor-specific immune responses in liver-metastasized MSS CRC.^345^ Furthermore, the combination of trametinib and durvalumab was found to be tolerable in refractory MSS mCRC patients, though it did not meet efficacy criteria for further progression in clinical trials.^346^

Ipilimumab, a monoclonal antibody targeting CTLA-4, has been approved by the FDA for the treatment of melanoma and specific lung cancers.^347^ It is actively under investigation in numerous clinical trials for its potential in treating CRC in combination with other therapeutic agents. In a particular study, the objective was to determine the optimal phase II dosages for a regimen involving regorafenib, ipilimumab, and nivolumab. The study found that this combination was particularly effective in MSS mCRC patients who did not have liver metastases.^348^ However, to corroborate these observations, randomized controlled trials are necessary. Additionally, another research initiative reported that pseudoprogression was infrequent in MSI/dMMR mCRC patients treated with nivolumab and ipilimumab. This combination therapy demonstrated impressive rates of disease control and survival.^349^ The completed MAYA trial revealed that pre-treatment with temozolomide followed by low doses of ipilimumab and nivolumab led to lasting therapeutic effects in patients with MSS mCRC and silenced O6-methylguanine-DNA methyltransferase.^350^

A completed trial evaluating ticilimumab as a standalone treatment showed minimal effects, although one patient exhibited a mild response and 21 patients survived beyond six months. These outcomes suggest potential benefits in combination with other ICIs^351^. Additionally, the safety of combining Y90 radioembolization with durvalumab or durvalumab and tremelimumab has been confirmed. In another study (NCT04258111), the efficacy and safety of IBI310 in combination with sintilimab were evaluated in patients with locally advanced or MSI-H/dMMR mCRC. In summary, while anti-CTLA-4 monotherapy in CRC has shown limited efficacy, its potential is significantly enhanced when combined with other ICIs, such as anti-PD-L1 agents.

Concerning LAG-3 as an immunotherapeutic target, favezelimab, an anti-LAG-3 IgG4 monoclonal antibody, shows promise. It disrupts the interaction between LAG-3 and MHC II molecules, enhancing the production of key cytokines and expression of activation markers in T cells.^352^ Favezelimab’s safety and efficacy profiles are currently being evaluated in a phase I/II clinical trial in combination with pembrolizumab.^353^ Initial results suggest a manageable safety profile, paving the way for further studies on LAG-3 targeted therapies in CRC. Another ongoing study involves XmAb®22841, either as a monotherapy or in combination with pembrolizumab, to establish its maximum tolerated and/or recommended doses. This study aims to assess various clinical parameters including safety, tolerability, pharmacokinetics, immunogenicity, and antitumor efficacy in patients with advanced solid tumors.^354^ XmAb22841 is a bispecific antibody engineered to target CTLA-4 and LAG-3, both of which are critical immune checkpoint receptors. By blocking these pathways, XmAb22841 may enhance T-cell activation and proliferation more effectively than single-receptor inhibition.

### Cancer vaccines

Cancer vaccines in CRC are designed to provoke a robust immune response against cancer-specific antigens. Unlike prophylactic vaccines, therapeutic cancer vaccines aim to treat existing cancer by priming the immune system to recognize and attack tumor cells. An array of vaccine platforms, including peptide vaccines, cell vaccines, and viral vector-based vaccines, are under investigation in CRC (Fig. 8).

Synthetic peptide-based cancer vaccines harness the immune system’s capabilities to target cancer cells by introducing specific antigens that stimulate an immune response. These vaccines offer benefits such as ease of production and the ability to be tailored to specific antigens, making them attractive candidates for cancer immunotherapy. Nevertheless, their efficacy is generally modest, necessitating the use of adjuvants to enhance the immune response. A notable phase II trial assessed the application of a 13-mer mutated K-Ras peptide as an adjuvant vaccine in CRC and pancreatic cancer.^355^ This peptide includes the prevalent G12V mutation and spans 13 amino acids. The study involved a cohort comprising five pancreatic cancer patients, seven CRC patients, and twelve individuals without active disease. Notably, this peptide triggered an increase in IFN-γ mRNA expression in nearly half of the participants. The observed median disease-free survival (DFS) was 35.2+ months and OS was 44.4+ months in the pancreatic cancer subgroup, while in the CRC subgroup, the DFS averaged 27.2+ months with an OS of 41.5+ months.^355^

Further advancing this approach, Rahma et al. explored combining the mutated K-Ras vaccine with cytokines such as IL-2 and GM-CSF to potentiate the immune response in patients with solid metastatic tumors, including CRC.^356^ Their study included a diverse patient group with cancers of the colorectum, pancreas, lung, and common bile duct, divided into three different treatment arms. The findings highlighted a significant variation in immune response across the groups, with the highest response observed in one of the treatment arms (92.3%). This study underscored that while GM-CSF could enhance vaccine efficacy, the addition of IL-2 did not improve and potentially impaired the immune response, necessitating further investigation into its role.

In relation to other potential targets, proteins such as TOMM34 and RNF4 are frequently overexpressed in CRC patients and thus represent promising targets for vaccine-based strategies.^357^ A phase II clinical trial evaluated the cytotoxic T lymphocyte (CTL) response to a peptide cocktail combined with uracil–tegafur (UFT/LV) chemotherapy as adjuvant immunotherapy. This trial enrolled 44 patients, who were divided based on their HLA-A*24:02 compatibility. Positive CTL responses to the peptides were observed in both matched and unmatched groups, with notably higher 3-year relapse-free survival (RFS) rates in the CTL-positive participants.^358^

Expanding on this approach, Hazama et al. conducted a phase II trial assessing a five-peptide vaccine cocktail in combination with standard chemotherapy regimens (FOLFOX, XELOX) in patients with advanced CRC.^359^ The trial built upon phase I findings indicating safety and minimal systemic adverse reactions. Noteworthy outcomes included longer OS in HLA-A*24:02-matched patients who received the vaccine for over a year, and the identification of the neutrophil–lymphocyte ratio as a predictive marker for treatment response. This trial highlighted both the potential and limitations of peptide-based vaccines, suggesting the necessity for a phase III trial to confirm these findings in a broader patient population.

Nucleic acid-based vaccines, particularly those utilizing mRNA technology, have demonstrated substantial efficacy in eliciting both humoral and cellular immune responses against tumor antigens. These vaccines are engineered in vitro to encode tumor-specific antigens that provoke an immunogenic response potentially capable of overcoming previous resistance observed in cancer vaccine applications.^360^ Currently, an mRNA vaccine, mRNA-5671/V941, targeting prevalent KRAS mutations (G12D, G12V, G13D, and G12C), is under evaluation in a phase II clinical trial (NCT03948763) to determine its safety, tolerability, and optimal dosing regimen. This vaccine was developed through a collaboration between Moderna and Merck. Concurrently, a phase I study is investigating its efficacy both as a standalone therapy and in combination with the immune checkpoint inhibitor pembrolizumab.^361^ Administered intramuscularly encapsulated within lipid nanoparticles, this vaccine is given over nine cycles every three weeks. Preliminary results suggest that mRNA-5671 is well-tolerated and capable of inducing an antitumor response. Upon uptake by APCs, the mRNA-encoded tumor antigens are processed and presented via major histocompatibility complexes (MHCs), thereby initiating CTL and memory T-cell responses.

Liu et al. have discussed several mRNA vaccines that are currently being evaluated in early-phase trials for their efficacy against melanoma and other malignancies. These vaccines, including TriMix, BNT111, mRNA-4157, and BNT122, encode not only tumor antigens but also immunomodulatory molecules and inflammatory cytokines.^362^ A phase II trial highlighted a robust CD8 + T-cell response elicited by the TriMix vaccine, which includes a tumor-associated antigen (TAA) mRNA, in stage III and IV melanoma patients. Moreover, BNT111, which encodes four TAAs, has shown potent immunotherapeutic potential in melanoma, especially when used in conjunction with checkpoint inhibitors.^363^ Another innovative approach involves a neoantigen-based mRNA vaccine (RO7198457; NCT03289962) developed by BioNTech and Genentech, which is currently being tested in a phase I trial across various cancer types, including CRC. This vaccine has been administered both as a monotherapy and in combination with the checkpoint inhibitor atezolizumab, showing a favorable safety profile and inducing both cytokine release and a peripheral T-cell response. A subsequent phase II trial is in the recruitment phase to further evaluate the efficacy of RO7198457 in CRC patients with detectable circulating tumor DNA post-surgical resection (NCT04486378).^364^

DNA vaccines are comprised of circular plasmids that encode specific tumor antigens, which are pivotal in activating targeted immune responses against cancer cells.^365^ These vaccines function by transporting the encoded genetic material into the nucleus of host cells, where it undergoes transcription and translation to produce the relevant antigens. Subsequently, these antigens are processed in the cytoplasm and presented on both MHC class I and II molecules, thus eliciting CD8+ and CD4 + T-cell responses respectively.^365^ Typically, the antigen presentation pathway involves either direct presentation to CD8 + T cells via MHC I, release and capture by APCs after secretion or cell death followed by presentation to CD4 + T cells via MHC II, or direct transfection and presentation on both MHC I and II in APCs. The versatility of DNA vaccines allows for the encoding of multiple antigens regardless of their molecular size, offering high specificity and safety with relatively low production costs. However, despite these advantages, they have exhibited limited immunogenicity, which has curtailed their therapeutic efficacy in clinical settings.^366^

In preclinical settings, Duperret et al. evaluated a synthetic neoantigen-based DNA vaccine, designed to enhance the immune response against tumor-specific neoantigens. The vaccine, which included strings of multiple epitopes bound to MHC I, was observed to increase CD8 + T cell responses with cytolytic activity, as indicated by the expression of the degranulation marker CD107 and the release of multiple cytokines such as IFN-γ, TNF-α, and IL-2. This study provided promising insights into the potential of tailored DNA vaccines to induce robust antitumor responses in a murine model, suggesting avenues for further development and clinical translation.^367^ In clinical trials focusing on CRC, the therapeutic potential of DNA vaccines has been explored. Gribben et al. investigated ZYC300, a DNA vaccine encapsulated in biodegradable poly-DL-lactide-coglycolide microparticles, which encodes the enzyme cytochrome P450 1B1 (CYP1B1). This enzyme is linked to the activation of procarcinogens, and hence, targeting it could potentially exert antineoplastic effects on cells expressing CYP1B1. In a phase I trial involving patients with advanced CRC, the vaccine was administered in differing doses: five patients received 12 doses while the rest received six doses. Among these, patients who developed an immune response to CYP1B1 exhibited stable disease or responsiveness to subsequent salvage therapies, suggesting a possible link between immune response development and therapeutic efficacy.^368^ Additional studies, such as a phase I trial (NCT00381173), have assessed the combination of ZYC300 with the chemotherapeutic agent cyclophosphamide in various cancer types including CRC, though the outcomes were inconclusive. Moreover, a phase I/II trial explored the immunogenicity and safety of a DNA vaccine encoding the DOM-CAP-1 fusion gene, targeting a peptide from carcinoembryonic antigen (CEA) in CRC patients. The results demonstrated significant immunological responses, particularly in patients with measurable disease, underscoring the potential of DNA vaccines to diminish peripheral tolerance in both normal and cancerous tissues.^369^

In cell-based vaccines, cells are used to stimulate the immune system to attack cancer cells. There are two main types of cell-based vaccines: tumor cell-based vaccines and dendritic cell (DC)-based vaccines. A phase II study enrolling three patients with CRC and liver metastasis explored the effect of the Vigil™ autologous vaccine, a novel dual-modulatory autologous tumor cell-based vaccine. In this vaccine, cells are transfected with a DNA plasmid encoding a granulocyte–macrophage colony-stimulating factor (GM-CSF) transgene and a bifunctional shRNA construct to knock down furin convertase and prevent GM-CSF degradation by Tgfb1 and Tgfb2. In the study, the vaccine was used in combination with folinic acid (leucovorin), 5-FU, and oxaliplatin (FOLFOX-6) chemotherapy.^370^ Two patients showed a disease-free survival (DFS) of over 8 years after receiving 12 doses of Vigil with FOLFOX-6. This study demonstrated a significant induction of long-lasting systemic adaptive immunity among patients. Vigil, in combination with FOLFOX-6, was found to be safe and exhibited a potential antitumor effect against advanced CRC with resectable liver metastases.^370^ A clinical trial in patients with advanced cancer, including CRC, also demonstrated the potential of Vigil to induce an immune response that correlates with prolonged survival.^371^ All of these findings point to Vigil™ as a potential treatment option for people with advanced colorectal cancer that is worth further investigation and development. Hu et al. reported the outcomes of a clinical trial that enrolled 254 patients with stage II and III CRC to test adjuvant active specific immunotherapy with an autologous tumor cell-bacillus Calmette-Guerin vaccine (OncoVAX®). This vaccine comprises irradiated autologous tumor cells with weakened live bacillus Calmette-Guerin as an immune adjuvant to prevent CRC recurrence following surgery.^372^ This trial was more effective in resectable treated rather than resectable alone. A significantly longer recurrence-free period and a 61% reduction in disease recurrence were observed. Phase III of the clinical trial revealed a notable beneficial effect of OncoVAX on the recurrence-free interval (57.1% relative risk reduction), overall survival (OS; 5 years), and recurrence-free survival (RFS; 5 years) among patients with stage II CRC.^373^ These results pave the way for new developments and underscore the importance of further research to unravel the potential effects of combining adjuvants with vaccines for enhancing treatment strategies in colorectal cancer.

Apart from autologous tumor cell-based vaccines, DC-based vaccines have been extensively tested in preclinical and clinical trials.^374^ DC-based vaccines are made by taking patients’ DCs and loading them with tumor antigens. Loaded DCs are then injected back into patients to train the immune system to recognize and attack cancer cells.^374^ A phase II clinical trial assessed the effect on disease progression and clinical benefits of autologous tumor lysate-pulsed DC immunotherapy with cytokine-induced killer cells in a small cohort of patients with GC and CRC. A total of 46 patients were enrolled in the study, with 14 and 13 patients randomly assigned to the cell-based immunotherapy group and control group, respectively.^375^ Patients who received cell-based immunotherapy combined with low-dose chemotherapy had higher interferon-gamma (IFN-γ) and interleukin (IL)-12 levels than controls. Additionally, patients who received cell-based immunotherapy had a lower risk of disease progression after surgery (*p* < 0.01) and longer OS (*p* < 0.01). These results suggest that DC/cytokine-induced killer immunotherapy is a promising and effective treatment for GC and CRC. This study emphasizes the value of combining chemotherapy or radiotherapy with DC/cytokine-induced killer immunotherapy, paving the way for further improvements in treatment efficacy.^375^ Combining immunotherapy with chemotherapy is crucial for treating CRC; however, the dosage plays a pivotal role in determining the outcome of these treatment modalities. At Duke Cancer Institute, Morse et al. evaluated the effectiveness of a CEA RNA-pulsed DC cancer vaccine and RFS in patients with resected liver metastases from colon cancer.^376^ The CEA RNA-pulsed DC cancer vaccine used DCs to deliver an RNA encoding the CEA protein. This protein is often found on the surface of cancer cells.^376^ In this trial, patients underwent leukapheresis, and their cells were then exposed to recombinant human-GM-CSF and recombinant human-IL-4 in a medium to generate DCs. They were loaded with mRNA encoding CEA. This phase I/II clinical trial revealed the safety and possibility of using mRNA-loaded DCs in patients with advanced malignancies.^376^ Therefore, using the patient’s own dendritic cells loaded with tumor antigen is a safe and practical method that raises the possibility that mRNA-loaded DCs could be used as an effective treatment for advanced cancers. This bolsters the continuous endeavors to utilize the immune system’s potential in combating malignancy. Another randomized clinical trial in patients with resectable mCRC used autologous tumor lysate-pulsed DCs and CD40L.^377^ After tumor resection, the tumor was irradiated and lysed in three freeze–thaw cycles in liquid nitrogen. DCs isolated from patients’ own peripheral blood mononuclear cells and transfected with recombinant human CD40L were loaded with tumor lysate to generate autologous tumor lysate-pulsed DCs expressing CD40L. This trial demonstrated increased IFN-γ levels in 15 of 24 patients, indicating T-cell proliferation. The 5-year RFS rate was 63% in responders and 18% in non-responders (*p* = 0.037). This work adds significant knowledge to the expanding corpus of research demonstrating the function of autologous tumor lysate-pulsed DCs in boosting immune responses and maybe benefiting long-term outcomes in patients with resectable mCRC.

Dendritic cell vaccines involve the isolation of a patient’s dendritic cells, loading them with tumor antigens, and reinfusing them to elicit a potent T cell response. Clinical trials have shown the potential for these vaccines to induce durable responses, and ongoing research is focused on optimizing antigen selection and delivery methods.^378^ Such vaccines have shown potential in generating durable immune responses and are currently being evaluated in combination with other immunotherapies.

Vaccination strategies in CRC are designed to induce robust and specific anti-tumor immune responses by targeting tumor-associated antigens (TAAs) or neoantigens. TAAs are proteins preferentially expressed in tumor cells, while neoantigens arise from tumor-specific mutations. Personalized cancer vaccines based on neoantigens have demonstrated promise in preclinical models and early-phase clinical trials by eliciting T cell-mediated responses that can be further potentiated when combined with ICIs.^379^

### T cell therapy

The ongoing advancements in basic research on chimeric antigen receptor T-cell (CAR-T) immunotherapy are notably propelled by continuous investigative efforts. Several emerging CAR-T therapeutic strategies have shown promise in both preclinical settings and early clinical trials for CRC treatment. The primary objective of CAR-T therapy is to pinpoint optimal antigens or synergistic combinations of innovative checkpoint inhibitors or mAbs, aiming to expand the therapeutic options available to CRC patients and provide durable clinical outcomes.^380^ CAR-T therapy, a transformative approach in cancer immunotherapy, involves extracting T cells from a patient’s blood, genetically engineering them to express a specific chimeric antigen receptor, and reinfusing them back into the patient. This method enables precise, exclusive, and personalized treatment modalities. Originally developed in 1989, CAR-T therapy has established itself as a revolutionary technique by demonstrating significant safety and enduring clinical responses, although it is associated with severe side effects such as cytokine release syndrome.^381^ The engineered T cells are designed to produce functional chimeric receptors capable of recognizing cancer-specific antigens without targeting normal tissues, in a non-MHC restricted manner, suggesting the potential to develop T-cell receptors (TCRs) with any desired specificity.^382^ The efficacy of CAR-T immunotherapy is enhanced by its ability to exhibit improved selectivity and cytotoxicity towards major histocompatibility complex (MHC) molecules, through the incorporation of a single-chain variable fragment (scFv) to the TCR, compared to traditional cell-mediated therapies.^383^ The CAR construct is composed of three main domains: the tumor-targeting scFv domain which aids T cells in recognizing and binding to antigens on the tumor cell surface; a hinge or spacer domain that connects the scFv to the transmembrane domain, enhancing the flexibility and attachment capability of the scFv;^384^ and a transmembrane domain that integrates the extracellular and intracellular components, adding stability and effectiveness to the CAR-T cells. The intracellular portion typically includes essential signaling domains such as CD3, CD28, and CD8α.^385^ Current targets under investigation in clinical trials registered on ClinicalTrials.gov include HER2, epithelial cell adhesion molecule (EpCAM), and mesothelin, alongside antigens such as NK group 2 member D ligand (NKG2DL), MUC-1, and CD133, which are significantly overexpressed in CRC.^386^

Engineered T-cell receptor therapy represents a pivotal approach in adoptive cell therapy, wherein patients’ T cells are genetically modified to introduce a tumor-specific TCR gene sequence. This modification enables the T cells to target and recognize tumor antigens specifically via TCR-mediated mechanisms.^387^ Prior to the development and widespread research into CAR-T cells, TCR-T cell therapies were being explored. The foundational work in this field was conducted by Dembić et al. in 1986, who demonstrated the feasibility of altering T-cell specificity by transducing them with recombinant TCRα and TCRβ genes in a murine model, setting a precedent for future therapies.^388^ However, the application of TCR-T cell therapy in CRC remains in nascent stages, largely due to various challenges and limitations observed in early clinical trials. The efficacy and safety profiles of these therapies are still under investigation.^389^ A notable study by Parkhurst et al. in 2011 examined the use of TCR-engineered T cells targeting CEA in patients with treatment-resistant CRC. Despite initial indications of potential therapeutic benefits, the trial revealed significant issues: two patients showed disease progression within 5–6 months, and all participants developed severe colitis, suggesting off-target effects on healthy tissues. This led to the suspension of the trial, though it did underscore the feasibility and challenges of employing TCR-T cell therapy in metastatic CRC.^389^ Current clinical trials exploring TCR-T cell therapy in CRC are listed on ClinicalTrials.gov, with various statuses including suspended, terminated, recruiting, active non-recruiting, and completed, under the following NCT identifiers: NCT03970382, NCT01723306, NCT03431311, NCT03638206, NCT05124743, NCT05451849, NCT05292859, NCT06043713, NCT05194735, and NCT00496860. This therapeutic approach holds promise for addressing solid tumors, propelled by innovative developments in tumor immunology. A significant milestone in TCR-T cell therapy was reached in January 2022 with the FDA’s approval of tebentafusp, a bispecific TCR CD3 T cell engager that targets the gp100 peptide in the context of HLA-A*02:01. This approval marks a significant advancement in the treatment of metastatic melanoma and sets a precedent for the potential expansion of TCR-based therapies in other solid tumor indications.

ACT represents a personalized immunotherapy approach where immune cells with antitumor activity are expanded ex vivo and reinfused into the patient. In CRC, ACT can use TILs or genetically modified T cells, such as chimeric antigen receptor (CAR) T cells or T cell receptor (TCR)-engineered T cells (Fig. 8). TIL therapy involves the isolation of lymphocytes from resected CRC tumors, which are then activated and expanded in vitro before being reintroduced into the patient. This approach takes advantage of the patient’s own tumor-specific immune cells, and its effectiveness is being evaluated in ongoing clinical trials.^390^

### Viruses therapy

Viral vectors represent a cornerstone in the realm of cancer immunotherapy due to their inherent immunogenicity and the flexibility for genetic manipulation to express tumor-associated antigens. The utilization of recombinant viruses such as adenoviruses in oncological vaccines has demonstrated their capability to instigate both innate and adaptive immune responses. These vectors efficiently infect antigen-presenting cells, notably dendritic cells, facilitating the expression of encoded transgenes which subsequently prime cytotoxic T lymphocytes with high avidity to target malignancies. The immunogenic superiority of viral vector-encoded tumor antigens compared to those delivered with adjuvants has been documented, potentially attributed to virus-mediated pro-inflammatory responses.^391^ Despite the relative simplicity in generating recombinant viruses, some concerns persist, such as the induction of vector-specific neutralizing antibodies which could dampen therapeutic efficacy.^392^

Oncolytic viruses, particularly adenoviruses, are employed not only for antigen delivery but also for their direct oncolytic activities, selectively lysing tumor cells and potentially disrupting the immunosuppressive TME to enhance immunotherapeutic outcomes. In the context of CRC, virus-based strategies are under rigorous investigation. For instance, an exploratory phase II trial assessed an intratumoral influenza vaccine, revealing increased CD8 + T cell infiltration and a notable modulation of immune-related gene expression post-vaccination (NCT04591379).^393^ Preclinical evidence also supports the immune-modulatory efficacy of this approach in enhancing responses to immune checkpoint inhibitors by transforming ‘cold’ TMEs into ‘hot’, immune-active zones.^394^

Further advancing CRC vaccine development, the Ad5-GUCY2C-PADRE vaccine, a non-replicating adenoviral vector encoding the GUCY2C antigen linked to a helper T-cell epitope, has demonstrated promising results in inducing robust cytotoxic and humoral immune responses specifically tailored against CRC cells overexpressing GUCY2C, with minimal adverse effects.^395^ However, the presence of pre-existing neutralizing antibodies against the adenovirus vector was shown to potentially interfere with the immune response to the vaccine, highlighting a challenge in the clinical application of adenoviral vaccines.^395^ Moreover, the oncolytic adenovirus Ad5 [E1-, E2b-]-CEA(6D), engineered for enhanced immunogenicity against CEA, has shown potential in eliciting CEA-specific immune responses, despite prevalent pre-existing immunity against the adenovirus vector in the patient population, suggesting a viable strategy for overcoming immunological hurdles in vaccine development.^396^

### Bispecific antibodies

Bispecific antibodies (BsAbs) are an innovative class of therapeutic agents engineered to bind two different epitopes or antigens simultaneously. By recognizing both a tumor-associated antigen and a T-cell activating molecule (usually CD3), BsAbs can recruit and activate T cells in the vicinity of cancer cells, thereby facilitating targeted cell-mediated cytotoxicity (Fig. 8).

Catumaxomab is one example of a BsAb that targets EpCAM on tumor cells and CD3 on T cells. While initially showing promise, its clinical development was hampered by adverse effects related to its strong immune activation.^397^ More recently, novel BsAb formats with improved safety profiles, such as blinatumomab, have been approved for hematological malignancies and are under investigation for solid tumors like CRC.

The development of BsAbs is rapidly evolving, with new generations optimizing the balance between efficacy and toxicity. This includes the engineering of BsAbs that incorporate immune checkpoint blockade domains or that target additional immune regulatory pathways, potentially enhancing their therapeutic index in CRC.^398^

In the realm of CRC immunotherapy, the modalities discussed herein represent the cutting edge of our current understanding and therapeutic arsenal. The integration of multi-omics data—including genomic, transcriptomic, proteomic, and metabolomic analyses—has the potential to unveil novel biomarkers and therapeutic targets, enabling more precise and personalized immunotherapy approaches. Furthermore, spatial multi-omics is an emerging field that allows us to map these molecular features within the architectural context of the tumor and its microenvironment, providing insights into the spatial heterogeneity of immune responses and facilitating the design of more effective immunotherapies.^399^

### The interplay between signaling pathways and immunotherapy in colorectal cancer

The intricate network of intracellular signaling pathways within CRC cells is a crucial determinant of tumorigenesis and has a profound impact on the tumor immune microenvironment (TIME). A deeper understanding of the crosstalk between these pathways and the immune system is essential for optimizing immunotherapeutic strategies. Here, we dissect the influence of key signaling pathways on the efficacy of immunotherapy.

#### The interplay between signaling pathways and immunotherapy

##### WNT/β-catenin signaling

The WNT/β-catenin pathway plays a central role in CRC development and progression. Aberrant activation of WNT/β-catenin signaling has been linked to immune exclusion, characterized by a lack of T cell infiltration within the tumor. This immune desert phenotype is associated with resistance to ICIs due to the absence of a pre-existing anti-tumor immune response. Recent studies suggest that targeting WNT/β-catenin may sensitize tumors to immunotherapy by altering the TIME and enhancing T cell infiltration.^400^

##### MAPK/ERK signaling

The mitogen-activated protein kinase/extracellular signal-regulated kinase (MAPK/ERK) pathway is frequently activated in CRC through mutations in KRAS, NRAS, or BRAF. This pathway influences the immune landscape by modulating the expression of immune-related genes, including those involved in T cell trafficking and PD-L1 expression on tumor cells.^401^ Combining MAPK/ERK pathway inhibitors with ICIs is an area of active investigation that holds promise in overcoming resistance to immunotherapy.^402^

##### PI3K/AKT/mTOR signaling

PI3K/AKT/mTOR axis is another key oncogenic pathway implicated in CRC. It affects the immune system by regulating cell survival, metabolism, and growth, as well as by influencing the secretion of immunosuppressive cytokines and the expression of checkpoint molecules. Targeting this pathway has the potential to reverse immunosuppression and improve the response to ICIs.^403^

##### JAK/STAT signaling

Janus kinase/signal transducers and activators of transcription (JAK/STAT) signaling is involved in various cellular processes, including immune response regulation. In CRC, aberrations in this pathway can lead to an immunosuppressive milieu. Inhibiting JAK/STAT signaling may enhance anti-tumor immunity and has been proposed to synergize with ICIs, although this approach is still in the early stages of research.^404^

#### Implications of MSI status on immunotherapy response

##### MSI-high CRC and responsiveness to ICIs

CRC with MSI-H represents a unique subset with a heightened sensitivity to ICIs. MSI-H tumors are characterized by defective DNA dMMR and exhibit a high mutational burden, leading to the generation of numerous neoantigens that render them highly immunogenic.^405^ ICIs such as pembrolizumab and nivolumab have achieved remarkable success in this group, leading to their approval for MSI-H/dMMR CRC patients.^332^

The increased immunogenicity of MSI-H CRC is multifactorial. The abundance of neoantigens facilitates the priming and activation of T cells. Moreover, MSI-H tumors often display an inflammatory phenotype with upregulated cytokine expression and enhanced T cell recruitment. This immunologically hot environment may contribute to the higher efficacy of ICIs observed in MSI-H CRC.

### Strategies for modulating the TME to enhance immunotherapy in colorectal cancer

A major challenge in the field of cancer immunotherapy, particularly for CRC, is modulating the TME to overcome immunosuppressive barriers and enhance treatment efficacy. The TME consists of a complex matrix of cellular and non-cellular components that can impede effective anti-tumor immune responses. Herein, we discuss innovative strategies aimed at remodeling the TME to improve the outcomes of immunotherapy.

One promising approach is the combination of immunotherapeutic agents with other drugs that target various components of the TME. For instance, the use of agents that deplete immunosuppressive cell populations, such as Tregs and myeloid-derived suppressor cells (MDSCs), can enhance the efficacy of ICIs.^406^ Additionally, targeting the extracellular matrix (ECM) and associated signaling pathways can improve immune cell infiltration and function within the TME.^407^

Angiogenesis inhibitors, such as bevacizumab, have also been explored in combination with ICIs. These inhibitors can normalize tumor vasculature, potentially enhancing T cell infiltration and decreasing hypoxia, which is known to contribute to immune suppression.^408^ Furthermore, the administration of oncolytic viruses can selectively lyse cancer cells, thereby releasing tumor antigens and promoting a pro-inflammatory TME that is more amenable to immunotherapy.^409^

## Advancements in precision medicine

### Advancements in multi-omics approaches for precision medicine in colorectal cancer

Epigenetic dysregulation has been increasingly recognized as a pivotal factor in the pathogenesis of CRC. Through epigenomic profiling, various epigenetic alterations have been identified that are crucial for the initiation, progression, and drug resistance in CRC. Specific biomarkers such as SFMBT2, ITGA4, THBD, and ZNF304 have emerged as critical indicators for early CRC screening at the tissue level, while methylation patterns of genes like KCNJ12, VAV3-AS1, and EVC are being employed to stage CRC accurately.^410^ Additionally, methylation anomalies in SEPT9 and SDC2 detected in fecal samples are proving to be effective for non-invasive early detection of CRC.^411^ The therapeutic landscape is also evolving with the proposal to use DNA methyltransferase inhibitors to target methylation abnormalities in CRC treatment strategies. Histone modifications represent another layer of epigenetic regulation integral to CRC dynamics. One notable discovery involves the ubiquitination of HDAC3, which reduces histone acetylation levels, thereby promoting the expression of genes associated with cancer stem cells. This finding highlights a unique aspect of ubiquitination-mediated epigenetic control in CRC.^412^

Transcriptomic profiling has become an essential tool in uncovering biomarkers within the CRC microenvironment. This approach facilitates the evaluation of immune and stromal cell compositions within transcriptomic datasets. Variations in the TME are closely linked to distinct molecular tumor subtypes, and these variations correlate with tumor mutational burdens, which in turn influence both the prognosis and the responsiveness to immunotherapy in CRC patients.^413^ Utilizing single-cell RNA sequencing (scRNA-seq), researchers have pinpointed optimal targets for immunotherapy and elucidated the role of neutrophils in iron metabolism and migration processes specific to CRC.^414^ Single-cell transcriptomics holds promise for refining diagnostic and prognostic biomarkers and identifying actionable therapeutic targets for individual cancer patients. Furthermore, RNA-seq datasets have been instrumental in screening for diagnostic and prognostic biomarkers in colorectal cancer, pinpointing differentially expressed genes in the intestinal mucosa, and developing a risk score model to aid clinical decision-making. A seven-gene prognostic marker specific to colon cancer has also been identified through meticulous single-cell transcriptome analysis.^415^

Proteomics has become a fundamental tool in the discovery of novel candidate protein biomarkers that are potentially secreted into bodily fluids such as blood, urine, and saliva.^416^ In a notable study, Ahn et al. utilized a CNN classification approach to identify a panel of five proteins—SAA2, APCS, APOA4, F2, and AMBP—that are indicative of both early and advanced-stage CRC.^417^ Additionally, the role of PTMs in proteomics has been emphasized in relation to their critical contributions to early diagnosis, prognostic stratification, and therapeutic approaches in CRC.

Multi-omics approaches have been instrumental in elucidating the complex molecular framework of KRAS-mutant CRC. In their research, Chong et al. leveraged multi-omics data to delineate two distinct molecular subtypes within KRAS-mutant CRC, designated KM1 and KM2. Their comprehensive analysis, incorporating both proteomics and phosphoproteomics, highlighted significant differences in signaling pathways between these subtypes. Notably, they discovered that PI3K/AKT, MEK, and FGFR inhibitors were particularly effective for the KM2 subtype, while fluorouracil and CDK inhibitors showed higher efficacy in treating the KM1 subtype.^418^ This sophisticated molecular stratification provides crucial insights for crafting targeted and potent therapeutic strategies for KRAS-mutant CRC. In the realm of early-onset colorectal cancer (EOCRC), distinct molecular characteristics have been observed, such as elevated tumor mutation burdens, unique DNA repair signatures, variations in gene expression driven by DNA methylation and somatic copy number variations, and differing patterns of immune infiltration. Additionally, the kinase LMTK3 has been identified as a potential biomarker for EOCRC.^419^ Further multi-omics analysis has brought to light LRRC26 and REP15 as novel prognosis-related driver genes in CRC, alongside the identification of sixty-six putative susceptibility genes including DIP2B and SFMBT1. This extensive analysis has also shed light on the interactions between genetic susceptibility risk loci and CRC pathogenesis.^420^

### Advanced integration of single-cell spatial omics for the study and diagnosis of colorectal cancer

Spatial omics technologies have facilitated profound insights into the TME, identifying critical biomarkers, elucidating cell signaling, and metabolic pathways pertinent to CRC. The confluence of single-cell sequencing with spatial transcriptomics offers a nuanced understanding of cellular taxonomy and the spatial arrangement of cells within CRC tissues. Roelands and colleagues have effectively merged multi-omics with spatial transcriptomics in CRC studies, unveiling CD47/SIRPα as a pivotal biomarker in the transition from normal to tumorous colon tissue.^421^ Wang et al. utilized scRNA-seq combined with spatial omics to explore how microbiota distribution within tumors influences immune and epithelial cell functions, thereby accelerating cancer progression.^422^ Additionally, Peng et al. employed methodologies like single-sample gene set enrichment analysis (ssGSEA), gene set variation analysis (GSVA), pseudotime, and cell proportion analysis to delineate the intricate interactions between inflammatory cancer-associated fibroblasts (iCAFs) and stromal elements in the TME, which are instrumental in advancing tumor growth and metastasis.^423^ Analyses at the single-cell and spatial levels have confirmed the role of the interaction between FAP+ fibroblasts and SPP1+ macrophages in fostering a pro-fibrotic TME that impedes lymphocyte ingress, potentially conferring resistance to immunotherapeutic interventions. This interaction presents a viable therapeutic target in CRC.^424^ Additionally, the spatial localization of immunological markers within tumors could serve as predictive indicators of colon cancer metastasis.^425^ In a comprehensive study involving scRNA-seq and spatial transcriptomics on 97 matched samples, Wu et al. identified metabolically active MRC1 + CCL18 + M2-like macrophages predominantly in metastatic tumors and their peripheries. Notably, metastatic tumor cells appeared to evade the cytotoxic effects of these M2 macrophages, highlighting a complex dynamic between tumor and immune cells within the microenvironment.^426^ Utilizing both spatial transcriptomics and single-cell data, they noted specific localizations of CRC cells at the invasion front and observed interactions involving SPP1+ macrophages and HLA-G signaling, which augmented anti-tumor immunity and promoted the proliferative and invasive traits of CRC cells.^427^ These findings illuminate the intricate cellular communications within the CRC tumor microenvironment, offering vital insights for understanding tumor progression and devising effective therapeutic strategies.

Spatial proteomics also holds promise in identifying pertinent biomarkers for CRC. Utilizing the CODEX (CO-Detection by IndEXing) technology, a proteomics platform based on immunofluorescence, researchers have discovered that tertiary lymphoid structures are present in CLR tumors but absent in DII tumors, with CLR tumors exhibiting higher T cell populations in contrast to the immunosuppressive granulocyte and macrophage-rich environment of DII subtype tumors, correlating with poorer prognoses in DII patients.^428^ Levy et al. employed the GeoMX digital spatial profiler to identify spatial proteomic biomarkers for lymph nodes and distant metastases in colorectal adenocarcinoma, adding predictive value for metastasis assessments.^429^ Plattner et al. leveraged spatial omics data derived from patient-derived organoids to probe intracellular and intercellular signaling networks in colorectal cancer.^430^ Despite the advancements, spatial omics in CRC research faces several challenges. The requirement for sophisticated instrumentation, intricate sample preparation, and complex data analysis renders widespread application and standardization difficult. The need for numerous probes, antibodies, metal tags, and substantial computing resources contributes to high operational costs. Additionally, the relatively low resolution of current spatial omics methods complicates the differentiation between individual cells or subcellular components, limiting insights into the spatial structure and function within cells. Moreover, the large datasets generated necessitate specialized tools for data processing, analysis, and visualization, along with robust statistical methods and biological interpretations, presenting significant data management challenges.

### Analysis and clinical relevance of circulating tumor cells in colorectal cancer

Circulating tumor cells (CTCs) serve as emissaries of the primary tumor, migrating through blood and lymphatic systems to promote metastasis. CTC clusters, though infrequent in the bloodstream, exhibit significant resistance to apoptosis and heightened metastatic capabilities.^431^ Echoing Dr. Paget’s seed and soil theory, where the seed includes cancer stem cells and CTCs, and the soil is the TME, it’s recognized that most CTCs perish shortly after entering the bloodstream due to environmental and immune challenges.^432^ Only a resilient few survive, adapting to new conditions and potentially forming clusters with CTCs to foster a conducive microenvironment for metastasis. These cells are pivotal in liquid biopsy applications for detecting residual disease, monitoring therapeutic response, and predicting recurrence, thereby underlining their potential in early cancer diagnosis and personalized treatment strategies.^433^ Unlike other biomarkers, CTCs contain comprehensive biological and molecular data from cancer cells, facilitating single-cell analyses that provide insights into cancer evolution at various stages.^434^ CTCs are increasingly recognized for their role in early disease detection, therapy monitoring, and understanding disease progression, making them vital targets for cancer therapy. Over the past decade, research has not only confirmed the existence of CTC clusters but also elucidated their clinical significance. Despite the established prognostic value of CTCs, their routine clinical application is hindered by challenges in assay specificity and concerns about the reliability of CTC counts for early metastasis detection. Enhancing these assays with additional biomarkers could improve the precision of liquid biopsies for cancer screening, disease monitoring, and therapeutic response assessment.

Studies in CRC patients have shown that these clusters, composed not of malignant cells but of tumor-derived endothelial cells, can accurately differentiate between healthy individuals and early-stage CRC patients (IIa).^435^ Furthermore, liquid biopsies aimed at detecting tumor components in blood will capture not only tumor cells but also other vital cellular elements of the TME. CAFs, which drive tumor cell proliferation, migration, invasion, and drug resistance through their secretions, play a crucial role in cancer progression and metastasis.^436^ Recent research underscores the clinical relevance of CTCs in CRC for early diagnosis, prognosis, and treatment monitoring, highlighting their role in identifying key cancer-associated proteins and pathways.^437^ Agarwal et al. have linked the presence of CAF/CTC clusters to tumor growth and metastasis.^438^ Despite the identification of CAFs beyond primary or metastatic sites, direct evidence of CAFs in patient circulation remains scant.

The clinical importance of CTCs in CRC is becoming well recognized, yet challenges related to their low abundance and high variability persist, hampering broader clinical adoption as a biomarker.^439^ Targeting or disrupting CAF/CTC complexes, therefore, represents a promising avenue for cancer control and metastasis prevention.

### Role and diagnostic potential of extracellular vesicles in colorectal cancer

The exploration of extracellular vesicles (EVs), specifically exosomes, has emerged as a pivotal area in tumor research over the last decade, addressing key challenges in therapeutics, diagnostics, and prevention. Exosomes are small vesicles, typically ranging from 30 to 140 nm in diameter, formed through the endocytic pathway. They play a crucial role in cellular communication by transporting miRNAs, proteins, and lipids between cells, thus influencing various biological responses. These vesicles are abundant in all bodily fluids, making them accessible targets for liquid biopsy approaches in cancer research, particularly in CRC. Exosomes contain diverse molecules such as miRNAs, proteins, and mRNAs, which are consistently altered in individuals with CRC. Although research specifically targeting exosomes in CRC is still limited, the EVs released from CRC cells provide critical insights into key molecules and signaling pathways that contribute to the disease’s progression, metastasis, chemoresistance and TME modulation.^440^ The presence of tumor-derived EVs in circulating body fluids positions them as potential novel biomarkers for early detection, prognosis, and predictive purposes in CRC. The examination of non-coding RNA (ncRNA) contents within extracellular vesicles (EVs) presents a practical and efficient method for both the diagnosis and monitoring of CRC. The ncRNA cargo includes mRNAs, miRNAs that selectively target mRNAs, and lncRNAs, including circRNAs that function either by sequestering miRNAs or by influencing transcription through mechanisms such as epigenetic modifications or interactions with transcription factors.^441^ A study utilizing small RNA sequencing from blood samples of patients across various CRC stages identified miR-320c encapsulated in EVs as a potential biomarker for metastatic CRC.^442^ Advanced RNA sequencing techniques comparing CRC tissues with matched normal controls highlighted the downregulation of circLPAR1, a circRNA originating from the circularization of exons 3 and 4 of the lysophosphatidic acid receptor 1 (LPAR1) transcript. Notably, the levels of circLPAR1 in exosomal plasma were significantly lower in patients with CRC and varied with the stage of the disease (polyps vs. adenocarcinomas), increasing post-tumor resection. Functionally, exosomal circLPAR1, when internalized by CRC cells, binds to eIF3h, which in turn reduces BRD4 accumulation leading to inhibited cell proliferation and less invasiveness. This suggests that circLPAR1 could serve as a specific biomarker for CRC diagnosis, patient monitoring, and potentially as a therapeutic target to alter cancer cell behavior. However, the study did not explore the origin of exosomal circLPAR1 under normal physiological conditions or the mechanisms behind its decreased levels in CRC patients, whether due to local reduction at the tumor site, in healthy tissue, or a systemic decline. Further research by Dou et al. indicated that circRNAs are enriched in the EVs compared to the cancer cells themselves and that activation of KRas negatively impacts circRNA levels.^443^ Furthermore, the analysis of circulating DNA within exosomes offers a more sensitive approach to identifying specific cancer cell mutations compared to using cell-free DNA. This is crucial for precision medicine, particularly in identifying patients eligible for targeted therapies, such as EGFR inhibitors in cases of wild-type RAS. The encapsulation of DNA within EVs protects it from degradation, enhancing mutation detection. This approach is invaluable for cases where tissue biopsies from metastatic sites are not feasible and for monitoring changes in the mutation status of cancer cell subpopulations during treatment.^444^

In the realm of proteomics, the integration of mass spectrometry with the analysis of EVs has led to significant advancements in identifying potential biomarkers for CRC. A recent study utilizing a proteomic approach, complemented by machine learning algorithms, demonstrated the capability of EVs to not only identify individuals with cancer but also to ascertain the cancer’s tissue origin.^445^ This analysis was conducted on EVs derived from both tissue and plasma samples, encompassing 497 samples from both normal and cancerous origins. These EVs were categorized into Exo S, Exo L, and exomeres, and their protein contents were meticulously profiled. Impressively, this profiling achieved a sensitivity of 100% and a specificity of 92% in differentiating between cancer-afflicted individuals and healthy controls. Furthermore, this proteomic characterization of tissue-specific EVs facilitated the discrimination among melanoma, colorectal, pancreatic, and lung cancers.^445^ Additionally, a comprehensive study involving 100 participants, distributed evenly across healthy individuals, patients with early or late adenomas, and patients with adenocarcinomas ranging from stage I to IV, employed liquid chromatography–tandem mass spectrometry to analyze serum EVs. This study identified six proteins—GCLM, KEL, APOF, CFB, PDE5A, and ATIC—that could distinguish between states of health, early neoplasia, and advanced neoplasia.^446^ Other investigations have reported elevated levels of EVs containing glycosylated fibrinogen beta chain and beta-2-glycoprotein 1 in the plasma of patients with CRC compared to control groups. These markers have shown higher sensitivity and specificity for CRC diagnosis than traditional markers such as CEA and carbohydrate antigen 19-9, suggesting their potential utility in diagnosing early-stage CRC.^447^ In parallel, Shiromizu et al. conducted proteome analysis on EVs isolated from the sera of CRC patients and healthy controls, identifying annexins A3, A4, and A11 as peptides deriving from EVs with enhanced sensitivity in detecting stage II CRC.^448^ Further, the analysis of serum from stage III colon cancer patients revealed higher levels of EVs containing SPARC and LRG1, which were predictive of disease recurrence. This selective increase in SPARC and LRG1 in colon cancer EVs, as opposed to other cancers such as gastric, thyroid, or cervix cancers, underscores the specificity of these markers for colon cancer. It should be noted that SPARC has been previously identified as ectopically expressed in the stroma surrounding digestive tumors, but not by the cancer cells themselves.^449^ Moreover, a decline in EVs containing QSOX1, which originate from cancer-associated fibroblasts (CAFs), has been observed in the sera of CRC patients.^450^ Following the isolation of plasma EVs and subsequent data-independent acquisition mass spectrometry, Zheng et al. identified several proteins, including phosphorylated fibronectin 1, haptoglobin, calgranulin-B, and fibrinogen α chain, which were significantly associated with cancer progression from healthy states to colonic adenoma and adenocarcinoma, with fibrinogen α chain being particularly distinct.^451^ In terms of clinical outcomes, elevated blood concentrations of total EVs and CD133+ EVs prior to treatment correlate with shorter overall survival in patients with metastatic CRC. Higher levels of CD133+ EVs are also associated with a lower overall response rate to first-line systemic therapy, suggesting their potential role in risk stratification and treatment optimization for metastatic cancer.^452^ Additionally, CXCL7-enriched EVs have been identified as early response biomarkers in patients with liver metastases undergoing systemic chemotherapy, with levels decreasing post-secondary tumor resection, indicating metastatic lesions as a primary source of these EVs.^453^

### Organoid

Organoids possess certain characteristics and advantages that distinguish them from prior models, including their high success rate in deriving from tumors and their relatively straightforward culture methods, which render them a promising instrument for functional precision medicine applications. As a preliminary step toward the molecular-diagnostic prediction and assignment of experimental therapies, there have been limited studies exploring the correlations and predictive accuracy of organoid-based assays for standard-of-care treatments in CRC and other advanced malignancies. Vlachogiannis et al.^454^ assessed the treatment response of gastrointestinal cancer patients and their corresponding organoids to identical therapeutic agents. These organoid cultures were developed from tumors of patients who had undergone extensive prior treatments and were participating in various phase 1 to 2 clinical trials. The study examined the predictive capability of organoids in 21 pairs of clinical and ex vivo responses to treatments like paclitaxel for gastroesophageal cancer, EGFR-antibodies for CRC, and TAS-102 for CRC. Additionally, the response to regorafenib in patients and patient-derived organoid xenografts (PDO-xenografts) was compared. Remarkably, they documented a 100% sensitivity and 93% specificity in predicting treatment responses based on organoid assays, thereby laying the groundwork for more comprehensive investigations. Ooft et al.^455^ conducted the first systematic study, named TUMOROID, in 2019. This research compared the responses of organoids and patients to palliative chemotherapy and analyzed the drug response of 35 organoid cultures derived from 29 patients across several lines of treatment, including second-line irinotecan, a combination of second-line 5-FU and irinotecan, and first-line therapy with 5-FU and oxaliplatin. The organoids were subjected to drug combinations at various concentrations in standardized viability assays. Intriguingly, they found that the organoid assay predicted the treatment response accurately in 80% of cases involving irinotecan-based therapies, whereas the response to the 5-FU and oxaliplatin combination was not predictable. This finding aligns with another study that also found no correlation between patient and organoid responses to 5-FU and oxaliplatin combination therapy in organoids derived from peritoneal metastases of nine patients.^456^ The underlying reasons for these discrepancies, particularly with oxaliplatin-based therapies, remain uncertain, potentially involving pharmacological properties of the drug, its solvent, or the absence of microenvironmental components such as stroma, immune cells, or microbiota in the organoid models.

Further investigations into organoids’ utility in predicting radiation or chemoradiation responses in rectal cancer have also been reported.^457,458^ Yao et al.^457^ conducted the largest study in this area to date, assessing the chemoradiotherapy responses of 80 patients and achieving 78% sensitivity and 92% specificity in predicting patient responses using a microscopy-based method that measured organoid size post-treatment. Ganesh et al.^458^ analyzed a biobank of 65 rectal cancer organoids, finding a tentative correlation between organoid viability post-radiation and endoscopically observed patient responses in 19 cases. Wensink et al.^459^ provided a meta-analysis of existing data on the correlation between organoid and clinical responses across various tumor types, analyzing 17 publications including the studies mentioned above. They reported pooled sensitivity and specificity of 0.81 and 0.74, respectively, indicating a generally positive outlook on the predictive value of organoid-based testing for approved chemotherapeutic agents. However, caution is advised as most studies were not systematic clinical trials with rigorous protocols, often had small sample sizes, and employed diverse methodologies, which could affect the reliability of the data. According to Wensink et al.,^459^ the most robust evidence is available for CRC, primarily due to the largest studies conducted on chemotherapy^455^ and radiation.^457^

Ooft et al.^460^ reported on the SENSOR trial, the first published interventional study that aimed to allocate off-label or investigational drugs to advanced cancer patients based on organoid drug profiling. Despite the innovative approach, the study did not achieve its primary endpoint of a >20% ORR, and no objective clinical responses were observed among the treated patients, highlighting some of the current challenges in organoid-based precision medicine. These challenges include limited culture success rates, high patient dropout rates during the drug testing phase, and the inability of predicted therapies to benefit patients. The study’s small sample size should be considered, but its outcomes suggest that organoids may not yet be universally predictive of clinical drug effects. Future improvements in model systems or drug screening protocols might enhance outcomes in larger upcoming studies. However, as of now, organoids remain somewhat distant from being a routinely used predictive tool in the clinical practice of cancer medicine. Other modelsAh, welcome to the thrilling world of cancer immunology and multi-omics, a field that has been dramatically reshaped by recent advances in technology and methodology. As we delve into this topic, it’s essential to understand how these technological innovations have enabled us to explore the intricate dance between cancer cells and the immune system at an unprecedented resolution.

### Identification and validation of DNA methylation biomarkers for therapeutic targeting and response prediction in colorectal cancer

A synthesis of existing research has led to the recognition of potential molecular markers pertinent to CRC therapies. For instance, the solute carrier family 25 member 22 (SLC25A22) has been implicated in promoting DNA methylation in KRAS mutant CRC cells. Disruption of this pathway through SLC25A22 knockdown results in DNA demethylation and reactivation of protocadherins, subsequently inhibiting WNT/β-catenin signaling, diminishing stem cell traits, and reducing resistance to 5-FU.^461^ Although primarily investigated in cell lines, human tissue samples, and animal models, this pathway bears considerable promise for not only CRC treatment but potentially other malignancies as well. The exploration of predictive DNA methylation biomarkers, although not yet clinically validated, merits further investigation in clinical trials. LINE-1 elements, which constitute 17% of the human genome, exhibit hypomethylation linked with genome-wide hypomethylation, correlating with early-onset CRC and adverse outcomes. LINE-1 methylation status has been identified as a therapeutic marker, associated with the prognosis of patients undergoing oral fluoropyrimidine therapy for stage II or III CRC.^462^ Further studies have highlighted the role of DNA methylation in influencing the behavior of bystander CD8+ TILs. Zou et al.^463^ crafted a quantitative DNA methylation-based signature to assess CD8+ TILs, providing a valuable tool for the development of new methylation biomarkers and the identification of potential therapeutic targets. Previous research has documented the reduced expression of DMTN in CRC tissues. Overexpression of DMTN has demonstrated potential in curbing the invasion and metastasis of CRC cells, suggesting its utility as a therapeutic target in precision medicine strategies for CRC patients.^464^ The heat shock protein 90 (HSP90) inhibitor, ganetespib, has proven effective in affecting DNA methylation by downregulating DNMT expression, which correlates with global DNA methylation levels in CRC cell lines. Ganetespib represents a promising approach to modulating DNA methylation and reactivating silenced genes in CRC.^465^ The depletion of ubiquitin-like with PHD and ring finger domains 1 (UHRF1) in conjunction with histone deacetylase (HDAC) inhibition has been shown to trigger rapid DNA demethylation, reactivating silenced genes and significantly reducing CRC cell proliferation. This dual targeting strategy of UHRF1 and HDAC presents a potent therapeutic avenue for CRC.^466^ Elevated UHRF1 levels, paired with reduced tumor suppressor gene (TSG) expression, are inversely related to CRC progression and poorer patient survival, underscoring the importance of investigating key UHRF1 domains and their relevance in CRC prognosis and suggesting potential therapeutic routes.

The dMMR in colorectal tumors is closely associated with the CIMP, and dMMR serves as a predictive marker for the ineffectiveness of 5-FU-based adjuvant chemotherapy. A study assessed the role of 5-azacytidine in enhancing sensitivity in treatment-resistant CIMP-high patients receiving a combination of capecitabine and oxaliplatin.^467^ Although preclinical findings are promising, ample clinical evidence is still needed to confirm that epigenetic therapies can re-sensitize tumors to chemotherapy. Research into epigenetic therapies aimed at reprogramming tumor cells to increase their susceptibility to radiation and cytotoxic treatments appears promising. Inhibitors of DNMTs and HDACs can re-induce the expression of TSGs such as p16, RASSF1A, DAPK, and genes methylated in specific chemotherapeutic pathways.^468^ This “reprogramming” can enhance the sensitivity of tumor cells to cytotoxic agents. For example, MSS cell lines are more likely to exhibit chemosensitization to irinotecan following pretreatment with 5-azacytidine.^469^ The identification of methylation markers in CRC has proven instrumental in monitoring treatment responses and tailoring therapeutic strategies based on the methylation profile of the patient. For instance, MGMT hypermethylation in patients with advanced rectal cancer undergoingchemoradiotherapy with temozolomide has been associated with a favorable clinical response, whereas lack of MGMT methylation correlates with treatment resistance.^470^ This underscores the potential of individualized treatment plans based on epigenetic profiles.

Circulating tumor DNA (ctDNA) has been increasingly recognized as a pivotal diagnostic tool across various malignancies. In the terrain of cancer, epigenetic dysregulation is a hallmark, and its manifestations can be traced in liquid biopsies, including effusions, urine, stool, and blood samples. Numerous epigenetic indicators have been validated as effective for CRC screening and are also regarded as indicators of poor prognosis. These epigenetic biomarkers are instrumental in tracking the progression of cancer, the efficacy of treatment, and the potential recurrence across the cancer care continuum. Yet, the availability of DNA methylation biomarkers with prognostic significance, especially for patients undergoing chemotherapy, is limited. Biomarkers such as estrogen receptor 1, zinc finger protein 132 (ZNF132), and cytoplasmic polyadenylation element binding protein 1 are under investigation for their potential as prognostic and predictive markers in CRC contexts.^471^ A prospective cohort study encompassing 1493 high-risk individuals demonstrated the effectiveness of a singular ctDNA methylation marker, cg10673833, achieving an impressive sensitivity of 89.7% and a specificity of 86.8% in detecting CRC and its precancerous stages. This highlights the critical role of ctDNA methylation markers in the surveillance and prognostication of CRC.^472^ Additionally, the methylation of MLH1 on shores, regardless of genetic background, was found to be independent of promoter CpG island hypermethylation and MSI status.^473^ There is considerable evidence of CpG island hypermethylation contributing significantly to the reduced expression of protocadherin beta 3 (PCDHB3). This gene has been identified in prior research as a novel tumor suppressor gene in CRC, inhibiting the nuclear factor kappa-B (NF-κB) signaling pathway. Thus, PCDHB3’s expression and cellular localization are considered valuable prognostic indicators for advanced CRC.^474^ Methylation of retinoic acid induced 2 has been identified as an independent marker of poor prognosis in CRC, obstructing the protein kinase B (AKT) signaling pathway and curtailing CRC cell proliferation both in vitro and in vivo. There is also frequent hypermethylation of DIRAS1 in CRC, governed by its promoter methylation, suggesting its potential as a marker for poor prognosis.^475^ The lysine methyltransferase, suppressor of variegation 3–9 homolog 2, has been implicated in CRC prognosis and is known to promote malignant traits by tri-methylating the slit guidance ligand 1 promoter. ZNF331, frequently methylated and acting as a transcriptional repressor in CRC, has shown high specificity (98%) and sensitivity (71%) for CRC detection.^476^ The findings of Vedeld et al. further corroborate the methylation of ZNF331 in CRC, associating it with poor prognostic outcomes.^477^

### Harnessing CRISPR-Cas9 technology for precision oncology in colorectal cancer

Elucidating the genes that facilitate tumor evolution can provide vital insights into the onset and advancement of cancer. Comprehensive genomic screening serves as a robust methodology for identifying mutated genes, which showcase phenotypic transformations following pharmaceutical interventions or other stimuli. Gao et al. identified 44 essential genes for the propagation of colonic cancer stem cell-enriched spheroids. Notably, the involvement of principal cholesterol biosynthesis genes (HMGCR, FDPS, and GGPS1) underscores the potential of targeting cholesterol synthesis pathways in combination with standard chemotherapy for enhanced therapeutic outcomes in colon cancer.^478^ The tolerance of colon cancer to MEK inhibitors, which target the KRAS pathway, was explored by YU et al. By employing CRISPR for genome-wide knockout screening in a CRC cell model harboring a KRAS mutation, it was found that the gene GRB7 contributes to resistance against MEK inhibitors via the PTK pathway. The interaction between GRB7 and PLK1 reactivates the MAPK pathway, suggesting that a combination therapy involving PLK1 and MEK inhibitors could be efficacious in overcoming this resistance, thereby presenting a new avenue for treating KRAS-mutated colon cancer.^479^ Zhou et al. demonstrated that variations in the expression of histone modification factors correlate with drug resistance in tumor cells, highlighting the heterogeneity among patients. Their genome-wide CRISPR library screening linked the ZEB2 gene to resistance against 5-FU, providing insights that could facilitate personalized treatment strategies.^480^

Zhao et al. identified β-catenin-related target genes through genomic screening, revealing their roles in the proliferation, differentiation, metastasis, and angiogenesis of colon cancer. These findings suggest potential therapeutic and prognostic applications for targeting β-catenin in colon cancer.^481^ Additionally, Martin et al. explored the reliance of colon cancer cells with KRAS mutations on mitochondrial proteins. Their screening identified mitochondrial pathway components as crucial for the survival of these cells, proposing mitochondrial inhibition as a therapeutic strategy for KRAS mutant colon cancer.^482^ Hu et al. utilized CRISPR/Cas9 gene knockout to identify nine genes associated with colon cancer, which could serve as potential therapeutic targets. Elevated expression of CCT6A, RHOQ, and RRP12 was linked to lower survival rates, while high levels of UTP18, DDOST, YRDC, ACTG1, RFT1, and NLE1 correlated with improved survival outcomes.^483^ Chen et al. employed CRISPR/Cas9 to delineate the genetic interactions of chromatin regulatory factors (CRS) that influence drug responses in cancer, thus establishing a CRS gene interaction map to guide rational pharmacotherapy.^484^ Šuštić et al. identified ERN1 as a key modulator in the response of KRAS mutant colon cancer cells to MEK inhibitors. Through CRISPR-mediated knockout and subsequent genome-wide screening, they discovered that the ERN1-JNK-Jun pathway plays a critical role in regulating the sensitivity of these cancer cells to MEK inhibitors, suggesting a novel therapeutic target for overcoming resistance.^485^ Li et al. utilized genome-wide CRISPR gene knockout screening to identify genes involved in the regulation of oxidative stress, crucial in both tissue inflammation and tumorigenesis. Their study highlighted the potential therapeutic role of the glycan-binding protein galectin-2 in inhibiting colon cancer development.^486^

Choi and colleagues elucidated the therapeutic potential of the CRISPR/Cas12A system in colon cancer treatment. They demonstrated that Cas12A exhibits a preference for T-rich PAM sequences. By engineering mutations into Cas12A, they developed the LBCAS12A and LBABE8E variants. These modifications not only enhance the precision of this tool but also expand the scope of its applicability due to an improved targeting range by altering the PAM recognition specificity. This advancement positions the LBCAS12A and LBABE8E variants as promising tools for genome editing in cancer therapy.^487^ Li et al. employed the CRISPR/Cas9 system alongside single-guide RNA to rectify mutations in the β-catenin driver genes, pivotal in oncogenesis. Correcting these mutations not only restored normal gene function but also significantly impeded the proliferation of cancer cells. This approach heralds a novel avenue for gene therapy in oncology.^488^ Pothuraju and colleagues tackled the role of MUC5AC, a secretory mucin whose dysregulation is implicated in both the progression of colon cancer and the emergence of chemoresistance. Through CRISPR/Cas9 mediated knockout and subsequent in vitro and in vivo functional analyses, they elucidated the mechanisms by which MUC5AC contributes to tumorigenesis and drug resistance.^489^ Chakraborty et al. discovered the therapeutic potential of targeting the NPY/Y2R pathway in colon cancer, particularly in regulating angiogenesis. They employed CRISPR/Cas9 to knock out the VEGF-A gene, finding that its inhibition, coupled with a Y2R antagonist, effectively curtailed angiogenesis in treated mice.^490^

The RNA-binding protein HuR (ELAVL1) was found to enhance apoptosis significantly when knocked out using CRISPR/Cas9 technology. This author identifies HuR as a viable therapeutic target for colon cancer, given its role in tumoral growth and survival.^491^ ERO1α, a protein implicated in poor prognosis in colorectal cancer, was the focus of a study by Takei et al. Knocking out ERO1α via CRISPR/Cas9 revealed its role in promoting cell proliferation and mobility through interactions with cell surface integrin-β1, pointing to new therapeutic targets.^492^ Ngamkham et al. explored the overexpression of pyruvate carboxylase (PC) in colon cancer and its association with aggressive disease progression and poor survival. CRISPR-mediated knockout of PC substantiated its role in promoting tumorigenesis, offering a new target for therapeutic intervention.^493^ Oh et al. demonstrated that αTAT1, a tubulin acetyltransferase, modulates Wnt1 expression, thereby influencing microtubule acetylation and the malignant properties of colon cancer cells. CRISPR/Cas9-mediated knockout of αTAT1 effectively reduced tumor invasiveness and progression.^494^ In their work, Xia et al. identified membrane-associated loop CH protein 2 (MARCH2) as overexpressed in poor-prognosis colon cancer. Knockout of MARCH2 activated endoplasmic reticulum stress, inhibited cell growth, and induced apoptosis, highlighting its potential as a therapeutic target.^495^

Gunes et al. utilized CRISPR to upregulate the Klotho gene in colon cancer cell line Caco-2, observing a significant reduction in cell proliferation and a reversal of tumorigenic properties, which supports the pro-apoptotic role of Klotho in tumor cells.^496^ Chen and colleagues focused on FAPP2, a modulator of the Wnt/β-catenin signaling pathway, highly expressed in colon cancer cells. CRISPR/Cas9-mediated knockout of FAPP2 curtailed tumor growth and reduced tumorigenic potential, suggesting a significant role for FAPP2 in oncogenesis.^497^ Lastly, Li et al. studied the role of CLCA1 in colon cancer development. Contrary to expectations, knockout of CLCA1 via CRISPR technology led to enhanced proliferation and metastasis of tumor cells, indicating that high expression of CLCA1 suppresses Wnt signaling and the EMT process, thereby inhibiting tumor growth.^498^

## AI: Transforming the diagnosis and treatment of colorectal cancer

### Utilization of AI in the diagnostic paradigm of colorectal cancer

**Fig. 9:**
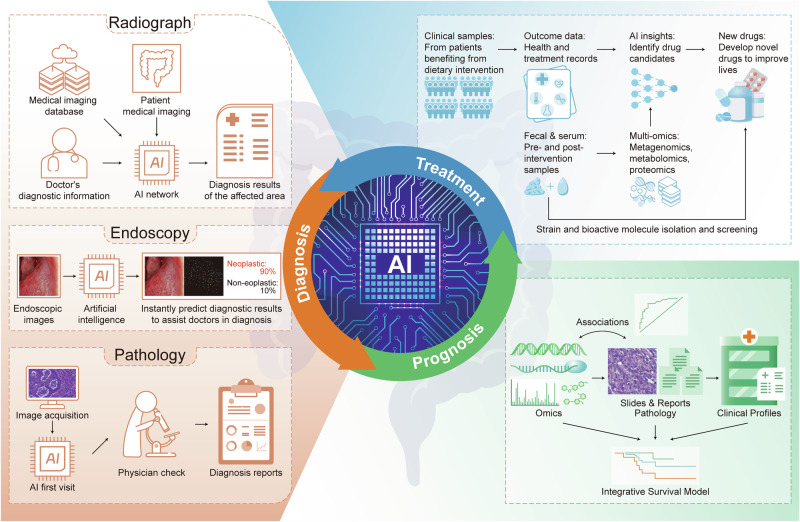
Artificial Intelligence: a paradigm shift in colorectal cancer diagnosis, prognosis, and therapy. This graph underscores the application of Artificial Intelligence in colorectal cancer diagnosis, prognosis, and therapy. The diagram details AI’s role in radiography, endoscopy, and pathology for diagnosis. The prognosis section depicts AI analyzing clinical profiles, pathology reports, and omics data to create integrative survival models, which predict patient outcomes and guide personalized treatment plans for colorectal cancer. It also illustrates the process of using AI for treatment, from analyzing clinical samples and health data to identifying drug candidates and developing personalized treatment plans

In the contemporary landscape of oncological diagnostics, AI is increasingly being recognized as a transformative adjunct to traditional methodologies, promising enhanced precision and accuracy in cancer detection. The integration of AI, particularly deep learning algorithms, into medical imaging and digital pathology represents a paradigm shift in our approach to cancer diagnosis, offering substantial improvements in the speed and reliability of image interpretation, workflow efficiency, image quality, and the incorporation of advanced 3D image reconstruction techniques^499^ (Fig. 9).

The synergy of AI with imaging modalities such as endoscopy and radiologic imaging (MRI/CT) is notably transformative. Convolutional Neural Networks (CNNs), a class of deep neural networks specialized in processing structured array data such as images, hold particular promise.^500^ These networks are adept at pattern recognition, learning from extensive datasets to identify and classify image features with minimal human intervention. CNNs, through their capacity for feature detection and translation invariance, provide a robust framework for enhancing the diagnostic utility of endoscopic imaging^501^ (Fig. 9). Granata et al. explore using radiomics and machine learning to evaluate growth patterns of colorectal liver metastases (CRLM) through MRI. They analyzed MRI scans of 81 patients, identifying specific imaging features that differentiate between expansive and infiltrative tumor growth. The study found that certain features from the portal phase of the MRI scans were particularly effective, achieving up to 92% accuracy with a machine learning model. This approach could help in better predicting tumor behavior and improving patient treatment strategies.^502^ Devoto et al. explore using texture analysis (TA) to develop a radiomic signature for early detection of hepatic metastasis in colorectal cancer patients. Analyzing CT images over five years, they found significant liver texture differences between patients who developed metastases and those who did not, particularly with coarse filtration. Patients who developed metastases had higher hepatic heterogeneity at presentation. Using TexRAD software, they identified medium to coarse texture features—mean intensity, standard deviation, entropy, and mean of positive pixels—as significant markers. This study suggests that TA could predict liver metastasis development, highlighting the need for further validation to confirm its clinical utility.^503^

The refinement of endoscopic technology has leveraged computer vision and segmentation techniques to differentiate between normal and pathological tissues with increasing accuracy, enabling more precise identification of neoplastic lesions. Recent studies have focused on reducing artifacts that may compromise diagnostic clarity by utilizing diverse datasets to improve segmentation algorithms^504^ (Fig. 9).

Innovative applications of CNNs in endoscopy have demonstrated the potential for real-time polyp detection. Misawa et al. developed an algorithm that showed promising results in identifying flat lesions, a notoriously challenging task, with a significant degree of accuracy when compared to expert annotations.^505^ Building upon this, Mori et al. conducted in vivo studies using an endocytoscope, achieving real-time differentiation of adenomas and hyperplastic polyps.^506^

These advances suggest that the integration of deep learning into endoscopic procedures could standardize detection rates, provide robust training tools for endoscopists, and potentially reduce unnecessary interventions. Despite the burgeoning application of AI in endoscopy, challenges persist in terms of technological advancement, regulatory frameworks, clinical validation, and the limitations of the available datasets which are critical for algorithm training.^507^

Radiologic imaging, including CT and MRI, similarly benefits from AI integration. The application of CNNs has shown promise in image detection, segmentation, and classification tasks, although literature specific to colorectal cancer applications remains sparse.^508^ Notably, AI has been instrumental in enhancing low-dose CT image quality, potentially reducing radiation exposure for patients. Deep learning has also been explored to improve attenuation correction of PET/MR images, aiding in the refinement of segmentation technologies^509^ (Fig. 9).

It is acknowledged that while AI augments imaging interpretation, it is not yet a replacement for human expertise. Collaboration between radiologists and computer scientists is crucial in developing tools that balance clinical efficacy with the potential reduction of human labor.^509^ The focus moving forward is on refining AI applications in colonoscopy and radiologic imaging, particularly in the detection of polyps and other abnormalities.

Nonetheless, CNNs and AI in imaging are not without limitations. Variability in anatomical structure and the presentation of artifacts can confound image interpretation, and segmentation challenges persist in discerning relevant information from inconsequential artifacts.^510^ Addressing these limitations requires ongoing optimization of AI technologies and a nuanced understanding of the complex interplay between computational models and biological variability.

The pursuit of enhanced pathological comprehension and genetic diagnostics is critical for augmenting the current nosological schema, with a focus on unearthing additional pathological and genetic etiologies of carcinogenesis. This endeavor is vital given that solely relying on histopathology or genetic profiles may be insufficient for the accurate diagnosis of CRC using AI (Fig. 9).

In the realm of AI, tumor classification via histopathological imaging has garnered some success. Yamashita et al. adeptly trained an algorithm for the classification of gastric and epithelial tumors, delineating them into adenocarcinoma, adenoma, or non-neoplastic categories.^511^ This process involved partitioning whole slide images into discrete tiles, which were subsequently assessed against a benchmark tile indicative of one of the three categories, culminating in a final classification. A comparative analysis between max pooling (MP) approaches and recurrent neural networks (RNNs) for image evaluation revealed a nuanced superiority in RNNs due to their capacity for information retention, although the statistical differences were not pronounced.^512^

The ramifications of delayed CRC diagnosis can lead to a plethora of complications. CNN models have showcased approximately 70% accuracy in discerning adenomatous from non-adenomatous polyps.^513^ This suggests that while the potential for AI to assist in CRC diagnostics is significant, optimization is essential. Dimitriou et al. contributed to this discourse by investigating methods to refine AI’s accuracy in stage II CRC prognosis, emphasizing the need to consider attributes beyond the tumor’s inherent characteristics, such as textural, spatial, and morphological traits. Their model, which incorporated machine learning to assess these features, demonstrated superior performance compared to the traditional pathological T staging, achieving notable AUROC values in both 5- and 10-year prognoses.^514^

Fuzzy logic systems, while theoretical in certain aspects of pathology, present a compelling case for application in the diagnosis of CRC. By allowing for gradations between binary states, these systems can accommodate the intricate variations encountered in pathological conditions. Fuzzy logic could be employed to assess a multitude of characteristics associated with colorectal polyps, potentially improving the predictive accuracy for cancer risk, which could potentially be extrapolated to CRC diagnostics.^515^

### Advancements in AI applications for colorectal cancer therapeutics

The efficacy of an AI model that leverages texture analysis of magnetic resonance (MR) images to predict complete therapeutic responses in rectal cancer patients undergoing neoadjuvant chemotherapy.^516^ The AI model’s receiver operating characteristic yielded an area under the curve of 0.86, indicating a high discriminative ability, with a 95% confidence interval ranging from 0.70 to 0.94. This model facilitates the early differentiation between complete response (CR) and non-response (NR) to therapy in the context of rectal cancer treatment (Fig. 9).

Furthermore, the pivotal role of AI in elucidating the drug metabolism associated with CRC has been acknowledged.^517^ AI technologies have been instrumental in deciphering the complex metabolic networks influenced by pharmacological interventions, thus elucidating the intricate metabolic transformations linked to CRC progression. Employing AI methodologies, researchers can now reliably characterize the metabolic networks specific to CRC, pinpointing key players in these metabolic pathways.^517^ Additionally, AI facilitates a dynamic representation of the rich metabolic network governing drug metabolism in CRC,^517^ significantly improving the processing of complex biological information networks (Fig. 9).

The predictive capabilities of AI in the realm of CRC, particularly employing artificial neural network (ANN) algorithms, are increasingly valued. ANNs are recognized for their nonlinear modeling capacity, offering flexibility that is particularly advantageous in medical research and clinical practice. Numerous advantages of ANNs include the enhancement of optimization processes, yielding cost-effective nonlinear models suitable for large datasets, and providing accurate and reliable predictions that support clinical decision-making. Furthermore, these models facilitate scholarly communication and the dissemination of knowledge.^518^ A systematic review of 27 studies (clinical trials or randomized controlled trials) utilizing ANNs for diagnostic or prognostic purposes indicated that majority studies showed improved healthcare outcomes, while the remaining ones demonstrated comparable results to traditional methodologies.^519^ Akbar et al. reported that ANN-based predictions of distant metastasis in CRC exhibited superior performance compared to logistic regression models in a cohort of CRC cases.^520^ Lu et al.^521^ crafted ML models capable of predicting genetic expressions related to immune checkpoints, including Tumor Mutational Burden (TMB), PD-L1 expression, and dMMR, categorizing them as either showing Durable Clinical Benefit (DCB) or No Durable Clinical Benefit (NDB). These models utilized a set of 359 human genes linked to immunotherapy response, immune cell infiltration, tumor-specific antigens, tumor markers, and key signaling pathways derived from RNA profiling data. Of these, the model distinguishing DCB from NDB demonstrated superior performance with an AUROC of 0.74, while the model differentiating PD-L1 positive from negative cases was less effective, achieving an AUROC of 0.52. In the context of CRC, the standard adjuvant chemotherapy regimen, FOLFOX, which combines fluorouracil, leucovorin, and oxaliplatin, poses challenges due to its neurotoxic side effects, which significantly impact patient quality of life. Chen et al. developed COLOXIS, an AI-based clinical decision support system, employing causal algorithms to predict the effectiveness of FOLFOX in CRC adjuvant therapy.^522^ COLOXIS assists clinicians in devising optimal treatment strategies while minimizing adverse effects. Empirical validation from large-scale clinical trials supports the efficacy of COLOXIS in enhancing predictive accuracy for chemotherapy responses, thereby fostering the advancement of precision medicine. Looking ahead, the deployment of AI-assisted nanorobots could potentially revolutionize chemotherapy by improving drug targeting and minimizing damage to healthy tissues. In our investigations, we initially employed scRNA-seq to delineate core transcriptional and protein markers. Subsequently, we leveraged a ResNet model to predict the efficacy of FOLFOX treatment. This prediction was based on a concise panel of five biomarkers derived from pre-treatment biospecimen samples. Remarkably, the accuracy of this predictive model reached an impressive 98.8%. This approach not only underscores the potential of integrating advanced computational models with molecular profiling but also enhances our ability to tailor chemotherapy treatments to individual patient profiles, thereby optimizing therapeutic outcomes.^523^

Hematoxylin and eosin (H&E) staining emerged as the predominant data modality utilized. Previous studies incorporated several machine learning (ML) methodologies, with supervised and weakly supervised learning being the primary strategies.^524^ Support Vector Machines (SVM) were notably effective in classifying various MSI statuses based on gene expression data. Subsequent multisite validation studies by Echle et al.^525^ confirmed the high performance of DL-based MSI prediction models from H&E images, with external validation cohorts showing AUROC values of 0.96. Additionally, high TMB has been predicted directly from H&E images, with three studies demonstrating the feasibility of this approach.^526^ Väyrynen et al.^527^ explored the prognostic implications of immune cell densities within TME using classical ML techniques. They found that higher densities of lymphocytes and eosinophils in the tumor-stroma were associated with better survival outcomes. Nestarenkaite et al.^528^ applied spatial analysis to assess immune cell migration across the tumor-stroma interface using the HALO multiplex IHC algorithm. Their findings indicated that the presence of CD8+ and CD20+ cells, as measured by immuno-gradient indicators, correlated with improved survival, whereas a pronounced infiltrative tumor growth pattern was linked to poorer outcomes. They also noted that MSI-H cases exhibited higher densities of CD8+ and CD68+ cells, with no significant differences in CD20+ cell densities between MSI and MSS statuses.

### AI-assisted drug discovery

The integration of AI in drug discovery heralds a transformative approach in the development of therapeutic agents, primarily due to its ability to process and analyze vast datasets to predict therapeutic outcomes. AI technologies streamline the drug development timeline and significantly lessen the financial burden traditionally associated with these processes. Particularly, the application of AI in drug repurposing enhances the efficiency and cost-effectiveness of identifying new uses for existing drugs.^529^

AI’s role extends across various stages of the drug discovery pipeline. Techniques such as machine learning are pivotal in forecasting the specific impacts of oncological treatments. Analytical methods including variance analysis, logistic regression, and advanced machine learning models like elastic net regression and random forests are employed to pinpoint molecular markers that predict responses to drugs.^530^ These capabilities are encapsulated in AI-driven platforms that integrate machine learning, predictive analytics, and data mining to advance drug development with clinical-stage assets.

However, the application of AI is not without limitations. AI models may not adequately capture the intricate dynamics of biological systems and molecular interactions, potentially leading to inaccuracies such as false positives or negatives in drug repurposing efforts. Moreover, AI-driven approaches often identify data patterns and correlations without elucidating the underlying mechanisms, which can impede the optimization and further development of repurposed drugs. The possibility of unforeseen side effects remains a significant concern due to the reliance on extensive but not always high-quality data sets. The pharmaceutical sector often grapples with the challenge of data sharing, emphasizing the need for platforms that not only offer vast data repositories but also ensure the data’s integrity.

## Challenges and future perspectives

In this section, we confront the myriad challenges impeding the seamless translation of our burgeoning understanding of CRC signaling pathways into clinical triumphs. Despite the strides made in disentangling the complex molecular web of CRC, numerous obstacles remain. These hurdles range from technological limitations to biological complexity, and from economic constraints to ethical considerations.

Firstly, we must acknowledge the intrinsic heterogeneity of CRC, both inter-tumor and intra-tumor, which obfuscates a universal therapeutic approach. The spatial multi-omics technologies that promise to demystify this heterogeneity are themselves in their infancy and are beset by issues related to sensitivity, resolution, and data integration. The analytical tools necessary to decipher the vast amounts of data generated by multi-omics are still under development, necessitating advancements in bioinformatics and computational biology.

The translation of bench-side discoveries to bedside applications is another significant challenge. The preclinical models that currently serve as the backbone of CRC research, while invaluable, are an imperfect mimic of the human condition. There is a pressing need for model systems that more accurately reflect the complexity of human CRC, including its microenvironment.

Moreover, resistance to targeted therapies remains a formidable foe. As we elucidate the signaling pathways involved in CRC, we uncover not only potential targets but also the resilience of cancer cells, which can adapt and develop resistance through alternative pathways or mutations. This calls for dynamic therapeutic regimens that can adapt to the evolving landscape of a patient’s cancer.

The economic burden of implementing precision oncology on a global scale, particularly in low- and middle-income countries, is another concern. The cost of high-throughput multi-omic analyses and subsequent personalized therapeutic interventions can be prohibitive, potentially exacerbating the disparities in healthcare outcomes.

Finally, ethical questions surrounding the management of patient data generated through AI and multi-omics must be addressed. Ensuring patient confidentiality and the ethical use of this information is paramount as we move into an era of data-driven medicine.

As we look to the future, we envision a multi-faceted approach to overcoming these challenges. Interdisciplinary collaboration will be crucial, combining the expertise of clinicians, scientists, bioinformaticians, and ethicists. Continued investment in research and development, along with a commitment to equitable healthcare delivery, will underpin the successful integration of these advanced technologies into routine clinical practice. Only through such concerted efforts can we hope to realize the full potential of targeted therapy for CRC, turning the tide of this global health challenge.

The road ahead is complex, but with each step, we move closer to a future where CRC treatment is not a one-size-fits-all proposition, but a tailored, dynamic, and patient-centric endeavor.

## Conclusion

In conclusion, our comprehensive review delineates the intricate web of signaling pathways integral to the pathogenesis and progression of CRC. The convergence of genetic alterations, epigenetic mechanisms, and environmental factors, including the influential roles of the immune system and gut microbiota, underscores the complexity of CRC. Moreover, the emergence of AI in oncology heralds a transformative era in the diagnosis, treatment, and management of CRC, offering new vistas for precision medicine.

AI’s integration into oncological research and clinical practice has demonstrated its potential to revolutionize the field by enabling the analysis of complex datasets, identifying novel biomarkers, predicting therapeutic responses, and personalizing treatment plans. The synergy between AI and targeted therapy is a testament to the ongoing evolution in our fight against CRC, empowering clinicians and researchers to anticipate the trajectory of the disease and to adapt therapeutic strategies accordingly.

Despite the promise of AI and targeted therapy, we recognize the challenges that lie ahead. These include the need for robust validation of AI algorithms, the ethical management of patient data, the development of resistance to targeted treatments, and the accessibility of such advanced interventions across diverse populations. Nonetheless, the strides made thus far provide a foundation for optimism.

Future research must focus on refining AI tools for better predictive accuracy, enhancing the precision of targeted therapies, and ensuring equitable access to these innovations. Moreover, the continued exploration of the molecular underpinnings of CRC will undoubtedly reveal new targets and pathways amenable to therapeutic intervention.

As we stand at the cusp of this new epoch, it is incumbent upon the scientific community to marshal our collective expertise towards the eradication of CRC. The interplay of targeted therapy and AI, underpinned by a deep understanding of CRC’s molecular landscape, will pave the way for more effective, personalized, and adaptive therapeutic regimens. Our concerted efforts will not only advance the frontiers of oncology but also offer hope to millions of individuals affected by CRC worldwide, propelling us towards a future where cancer is no longer a formidable adversary but a manageable condition.

## Data Availability

The data that support the findings of this study are available from the corresponding author upon reasonable request.
